# Discovery and characterization of a novel pathogen *Erwinia pyri* sp. nov. associated with pear dieback: taxonomic insights and genomic analysis

**DOI:** 10.3389/fmicb.2024.1365685

**Published:** 2024-05-09

**Authors:** Linxin He, Rong Huang, Haiyan Chen, Liang Zhao, Zhenfen Zhang

**Affiliations:** Key Laboratory of Grassland Ecosystem, Ministry of Education, Sino-U.S. Centers for Grazing Land Ecosystem Sustainability, Ministry of Science and Technology, Pratacultural College, Gansu Agricultural University, Lanzhou, China

**Keywords:** pear tree dieback, *Erwinia*, whole genome sequence analysis, pan-genome analysis, identification

## Abstract

In 2022, a novel disease similar to pear fire blight was found in a pear orchard in Zhangye City, Gansu Province, China. The disease mainly damages the branches, leaves, and fruits of the plant. To identify the pathogen, tissue isolation and pathogenicity testing (inoculating the potential pathogen on healthy plant tissues) were conducted. Furthermore, a comprehensive analysis encompassing the pathogen’s morphological, physiological, and biochemical characteristics and whole-genome sequencing was conducted. The results showed that among the eight isolates, the symptoms on the detached leaves and fruits inoculated with isolate DE2 were identical to those observed in the field. Verifying Koch’s postulates confirmed that DE2 was the pathogenic bacterium that causes the disease. Based on a 16S rRNA phylogenetic tree, isolate DE2 belongs to the genus *Erwinia*. Biolog and API 20E results also indicated that isolate DE2 is an undescribed species of *Erwinia*. Isolate DE2 was negative for oxidase. Subsequently, the complete genome sequence of isolate DE2 was determined and compared to the complete genome sequences of 29 other *Erwinia* species based on digital DNA–DNA hybridization (dDDH) and average nucleotide identity (ANI) analyses. The ANI and dDDH values between strain DE2 and *Erwinia* species were both below the species thresholds (ANI < 95–96%, dDDH<70%), suggesting that isolate DE2 is a new species of *Erwinia*. We will temporarily name strain DE2 as *Erwinia pyri* sp. nov. There were 548 predicted virulence factors in the genome of strain DE2, comprising 534 on the chromosome and 5 in the plasmids. The whole genome sequence of strain DE2 has been submitted to the NCBI database (ASM3075845v1) with accession number GCA_030758455.1. The strain DE2 has been preserved at the China Center for Type Culture Collection (CCTCC) under the deposit number CCTCC AB 2024080. This study represents the initial report of a potentially new bacterial species in the genus *Erwinia* that causes a novel pear dieback disease. The findings provide a valuable strain resource for the study of the genus *Erwinia* and establish a robust theoretical foundation for the prevention and control of emerging pear dieback diseases.

## 1 Introduction

Gansu Province is an important origin of pear (*Pyrus* L.) plants in China. The species and varieties of pears in Gansu Province rank first in the northwest region (Chang et al., 2017). There are more than 180 local varieties of pear and 250 types of cultivation, with a wide distribution across many areas (Xiao et al., 2022), in Gansu Province, China. China has a cultivation area of about half of the country and a yield >60%, Anhui, Hebei, Shandong, and Liaoning provinces are the concentrated pear production areas. In addition, Lanzhou City, Gansu Province, is famous for producing winter pears (Shi et al., 2023). In 2021, with the increasing convenience of forest fruit circulation across various regions of China, a pear tree disease known as pear fire blight of Xinjiang Korla fragrant pear (*Pyrus sinkiangensis* Yu) spread to Gansu Province (Sun et al., 2023). The diseased plant rate in some pear orchards is 100%, and the mortality rate of fragrant pear trees in areas affected by pear fire blight is as high as 90% (Ministry of Agriculture and Rural Affairs, PRC, 2021). Pear fire blight originated in New York state and the Hudson River Highlands in the United States in 1978. Fire blight is a bacterial disease that can cause disease and death in most plants of the Rosaceae family. *Erwinia amylovora* causes pear fire blight, while *Erwinia pyrifoliae* causes Asian pear fire blight. Both belong to the genus *Erwinia* in the family *Enterobacteriaceae* (Chen et al., 2023). Pear fire blight and Asian pear fire blight are currently the two main pear dieback diseases.

As reported by Piqué, after plants were infected with the pear fire blight pathogen *E. amylovora* (Piqué et al., 2015), the bark became ulcerated and the bark was reddish-brown under the ulcerated epidermis. Infected leaves expand and turn black (from the main leaf veins to the surrounding lateral veins), ultimately causing the entire leaf to wither, curl, and hang on the tree without shedding, resembling leaves that have passed through flames (Luminita et al., 2008). However, the bark of the Asian pear fire blight pathogen *E. pyrifoliae*, whose pathogenic characteristics and harmful symptoms are similar to those of *E. amylovora*, remains green under the black-brown spots on the epidermis (Kim et al., 2001). The spread of pear fire disease is very fast; it is now present in over 50 countries worldwide, including North America, Europe, North Africa, the Middle East, Oceania, and Asian countries (Drenova et al., 2013). For example, in 1998, the losses in the Northwest United States alone were estimated to exceed 68 million. In 1990, pear trees were uprooted from 328 hectares of pear orchards in Yugoslavia, resulting in economic losses exceeding 30 million US dollars (Panic and Arsenijevic, 1993). In Italy, approximately 500,000 fruit trees were destroyed due to this disease in 1997 (Calzolari et al., 1999).

The conventional approach to pathogen identification involves gathering diseased branches and leaves suspected to having the disease (such as pear fire blight) in order to isolate and purify the pathogen and assess its pathogenicity (EPPO Standards: Diagnostics, 2008). After conducting 16S rRNA sequencing analysis, conventional phenotypic identification and molecular identification are typically used to determine the taxonomy. However, this can lead to inaccuracies in the classification of species in the genus *Erwinia*, due to the significant resemblance and close association between the characteristics of *Erwinia* species and those in the genus *Pantoea* (John et al., 2012). Therefore, more accurate whole-genome sequencing methods and physiological and biochemical methods have become the first choice for identification (Gallegos et al., 2019). Combined with the API and Biolog methods are relatively commonly used for physiological and biochemical identification. API 20E assesses the utilization of sugar; the BIOLOG identification system (a carbon source-based microbial identification system) includes Biolog FF, Biolog YT, Biolog GP, Biolog AN, and Biolog GN (Piqué et al., 2015). The era of utilizing complete genome sequences for species classification has now commenced, due to the rapid progress in genome sequencing technology. In this context, the ANI and dDDH values, based on the complete genome sequence, have pivotal roles in classifying bacteria (Meier-Kolthoff et al., 2013). In short, whole-genome sequencing-based identification involving dDDH and ANI values has emerged as the new gold standard for accurately classifying bacterial species (Tambong, 2019).

This study isolated and purified the pathogenic bacteria from the infected tissues of pear leaves with a disease similar to pear fire blight, followed by morphological characterization, physiological and biochemical assays, and 16S rRNA-based identification of the obtained pathogenic bacterial strains. Subsequently, whole-genome sequencing was employed to precisely determine the taxonomic classification of the strain at the species level, thus identifying the bacteria that causes the disease and discovering it as a novel *Erwinia* species. This study is of great significance for targeted prevention and control of this disease and enriches the *Erwinia* species resources.

## 2 Materials and methods

### 2.1 Pathogen isolation

In July 2022, symptoms of a novel disease similar to pear fire blight were observed and recorded in a pear orchard in Shandan County, Zhangye City, Gansu Province (longitude 100° 41′ to 101° 42′ E; latitude 37° 50′ to 39° 03′ N), China. Ten sets of diseased leaves from three different pear trees were brought back to the laboratory for strains isolation. The diseased leaves were stored in kraft paper bags and stored at −4°C.

The pathogens were isolated following the method proposed by Han et al. (2022), briefly, to create bacteria suspensions, diseased leaves were cut into 1 cm^2^ pieces, surface-disinfected with 75% alcohol for 30 s and 1% sodium hypochlorite for 60 s, washed three times with 5 mL sterile water, placed in a sterilized mortar, ground with sterile water, and left to stand for 30 min. Three replicates were set for the pathogen isolation test. An inoculation loop was used to transfer a small amount of each pathogen suspension into NA (Nutrient Agar: Beef extract 3 g, Peptone 10 g, Glucose 2.5 g, Sodium chloride 5 g, Agar 15 g, Distilled water 1,000 mL, pH value 7.0 ± 0.1) culture medium. The NA culture medium was then placed in an incubator at 28°C with a 12/12 h light/dark cycle for 24–48 h. When colonies appeared on the NA culture medium, they were purified using the streak plate method. The represented bacterial isolates were designated DE1-DE8. All eight bacterial isolates underwent pathogenicity testing.

### 2.2 Pathogenicity testing

Pathogenicity testing of the eight bacterial isolates was conducted on healthy, young detached leaves and fruits (*Pyrus pyrifolia* var. *Sinensis* (Lindley)Y. Teng et K. Tanabe, *Pyrus ussuriensis* Maxim, *Pyrus brestschneideri* Rehd.) collected in Gansu Agricultural University that were surface-disinfected with 75% alcohol and rinsed three times with sterile water. Six inoculated leaves and fruits per isolate, and four replicate treats. An equal volume of sterile water was used as the control treatment.

Each of the eight bacterial isolates was added to tryptic soy broth (TSB) and cultured at 25°C for 24 h, centrifuged to obtain a pellet containing the bacteria, and adjusted with sterile water to an optical density at 600 nm (OD_600_) of 1.0 (to give a bacterial concentration of approximately 10^8^ CFU/mL). The abovementioned leaves and fruits were placed on the germination bed of a sterile growth bottle (Yao et al., 2021). A sterile inoculation needle was used to create three wounds per leaf, and a small piece (about 2 × 2 cm) of sterilized skimmed cotton was placed over the wounds. Next, 2 mL bacterial solution (10^8^ CFU/mL) was added to the cotton. A sterile scalpel was used to create a triangular wound on the fruit, measuring 2 cm in length, and the bacterial suspension was sprayed into the wound. A control experiment employed sterile water as a substitute for the bacterial suspension. Finally, 100 mL sterile water was added to the bottom of the bottle for moisturization. The bottle was placed at 23°C and 45% humidity in a group culture room with an 18/6 h light/dark cycle for incubation. Symptom onset on the inoculated leaves (spots similar to the disease symptoms in the field) was observed and recorded from 7 d. The morphology and 16S rRNA gene sequencing of the re-isolated and inoculated bacteria was verified to be the same, thereby verifying Koch’s postulates (Berraf-Tebbal et al., 2020).

### 2.3 DNA extraction and 16S rRNA gene sequencing of isolate DE2

After verification of Koch’s postulates, isolate DE2 was transferred to 100 mL NB (nutrient broth) medium for culture at 30°C and 160 rpm for 24 h. Thereafter, the OD_600_ was adjusted with sterile water to 1.0. The solution was then centrifuged (10,000 rpm for 1 min) to obtain the bacteria, which was washed with sterile water. A bacterial genomic DNA extraction kit (TIANGEN, Beijing, China) was used to extract the genomic DNA. Next, the 16S rRNA gene was PCR amplified (Schedule 1 provides the reaction mixture details) using universal primers 27F (5′-GAGTTTATCCTGGTCAG-3′) and 1492R (5′-AAGGGGTGGATCCAGCCGCA-3′) (Clarridge, 2004; Hassan, 2017) (Supplementary Table S1). The PCR products were separated by 1% agarose gel electrophoresis and then sequenced by Sanger method at Sangon Biotechnology Company (Shanghai, China).

### 2.4 Phylogenetic tree construction for isolate DE2

NCBI BLASTN^1^ and EZ BioCloud 16S-based ID^2^ tools were used for online comparison of isolate DE2 with known bacterial strains. The 16S rRNA sequences of 18 bacterial strains with high sequence similarity to isolate DE2 were selected to construct a phylogenetic tree using MEGA 11. Using the Neighbor-Joining method to construct the phylogenetic tree, with the Bootstrap value set to 1,000.

### 2.5 Determination of physiological and biochemical characteristics of isolate DE2

The physiological and biochemical characteristics of isolate DE2 were determined using an API 20E kit (BioMèrieux, France) and a GEN III Microplate (Biolog, United States) according to the manufacturers’ instructions.

### 2.6 Determination of fatty acid profile of isolate DE2

Fresh bacteria were scraped onto tryptic soy agar (TSA) medium and incubated for 24 h, transferred to a 15 mL Eppendorf tube, freeze-dried, and subjected to fatty acid analysis using the Sherlock Microbial Identification System (MIDI, United States) to analyze the fatty acid composition of isolate DE2.

### 2.7 Whole-genome sequencing, assembly, and prediction of isolate DE2

Isolate DE2 underwent whole-genome sequencing by Majorbio Biotech (Shanghai, China). First, to ensure that the DNA samples met the required standards for whole-genome sequencing, NanoDrop 2000 was employed to assess the DNA purity, and a Quantus Fluorometer (using PicoGreen reagent) was used to assess the DNA concentration.

Genomic DNA was sequenced using a combination of PacBio RS II Single Molecule Real Time (SMRT) and Illumina sequencing platforms. The Illumina data was used to evaluate the complexity of the genome.

For Illumina sequencing, at least 1 μg genomic DNA per strain was used for library construction. The DNA samples were sheared into 400–500 bp fragments using a Covaris M220 Focused Acoustic Shearer following the manufacturer’s protocol. Illumina sequencing libraries were prepared from the sheared fragments using a NEXTflex^™^ Rapid DNA-Seq Kit. Briefly, 5′ ends were first end-repaired and phosphorylated. Next, the 3′ ends were A-tailed and ligated to sequencing adapters. Thereafter, the adapter-ligated products were enriched by PCR. The prepared libraries were then used for paired-end Illumina sequencing (2 × 150 bp) on an Illumina HiSeq X Ten machine.

For PacBio sequencing, 15 μg genomic DNA was spun in a Covaris g-TUBE (Covaris, MA, United States) at 6,000 rpm for 60 s using an Eppendorf 5424 centrifuge (Eppendorf, NY, United States). The DNA fragments were then purified, end-repaired, and ligated with SMRTbell sequencing adapters (Pacific Biosciences, CA, United States) following the manufacturer’s recommendations. The resulting sequencing library was purified three times using 0.45× volumes of Agencourt AMPure XP beads (Beckman Coulter Genomics, MA, United States) following the manufacturer’s recommendations. Next, a ~ 10-kb insert library was prepared and sequenced on one SMRT cell using standard methods.

The PacBio and Illumina data were used for bioinformatics analysis using the Majorbio Cloud Platform^3^ (Shanghai Majorbio Bio-pharm Technology Co., Ltd.). The detailed procedures are as follows. The complete genome sequence was assembled using both the PacBio and Illumina reads. The original image data were transferred via base calling into sequence data, defined as raw data or raw reads and saved as FASTQ files. The FASTQ files (containing read sequences and quality information) are the original data provided for users. A statistic indicating quality information was used for quality trimming, during which low-quality data were removed to generate clean data. The reads were then assembled into a contig using a hierarchical genome assembly process (HGAP) and canu (Koren et al., 2017). The last circular step was checked and finished manually, generating a complete genome with seamless chromosomes and plasmids (Stothard and Wishart, 2005). Finally, error correction of the PacBio assembly was performed based on the Illumina reads using Pilon (Liu et al., 2020). Glimmer (Delcher et al., 2007) was used for coding sequence (CDS) prediction, tRNAscan-SE (Borodovsky and Mcininch, 1993) was used for tRNA prediction, and Barrnap was used for rRNA prediction. Using CGview website^4^ to draw a whole genome loop diagram of the assembled genome sequence.

K-mer analysis involves utilizing reads derived from second-generation sequencing, selecting high-quality sequencing regions, and estimating the genome size by analyzing individual K-mers at a base-by-base level. Genome coverage analysis employs two approaches: (1) K-mer-based approach: By leveraging the statistical patterns of K-mer distribution, the genome size is estimated, and this estimate is then juxtaposed with the actual genome assembly size to infer the genome’s coverage. This gives: Coverage = Assembled genome length / Estimated genome length based on K-mer analysis; (2) Reads-based approach: By constructing a library from the assembly results and performing soap analysis on the set of reads used for assembly, the length of the alignment is determined, and this length is then used to infer the genome’s coverage. This gives: Coverage = Sum of lengths of alignments/Length of assembly result.

### 2.8 GC–depth distribution of isolate DE2

As different genomes have different GC contents, the GC content can be used to determine whether there is any contamination from other species’ genomes in the samples. SOAP software was used to compare the raw paired-end library reads to the assembled sequence to obtain the base depth. A length (1, 3, 5, 8, 10 kb) was selected as a window, and the sequence was advanced base by base (or advanced without repeats), and the mean depth of each window was calculated along with the GC content to obtain a GC-depth distribution.

### 2.9 Phylogenomic comparison of isolate DE2

To further determine the classification of strain DE2, JSpeciesWS^5^ and the Genome-to-Genome Distance Calculator (GGDC)^6^ were used to calculate the average nucleotide identity (ANI) and digital DNA–DNA hybridization (dDDH) values between the complete genome of isolate DE2 and *Erwinia* strains with high similarity (from the NCBI genome database^7^). In generally, a dDDH value ≥70% or an ANI value ≥95–96% indicates that the strains are members of the same species. Conversely, ANI values <95–96% or dDDH values <70% indicate that the strain does not belong to any known species. A heatmap based on ANI values was generated using TBtools software (Yao et al., 2022).

### 2.10 Pan-genome analysis of strain DE2

To understand the direct genetic differences between strain DE2 and other *Erwinia* species, genome sequences of closely related *Erwinia* species were downloaded from the NCBI database. The complete genome sequences of strain DE2 and these other *Erwinia* species were then uploaded together to the Majorbio Cloud Platform.^8^ The pan-genomic analysis of strain DE2 is completed by this platform. The Pan-Genome Analysis Pipeline (PGAP) was used for pan-genome analysis (Ren et al., 2022).

### 2.11 Virulence factor annotation of strain DE2

The Virulence Factor Database (VFDB)^9^ was used to annotate the virulence factors encoded by strain DE2 (Chen et al., 2005).

## 3 Results

### 3.1 Field symptoms of a novel disease similar to pear fire blight

**Figure 1:**
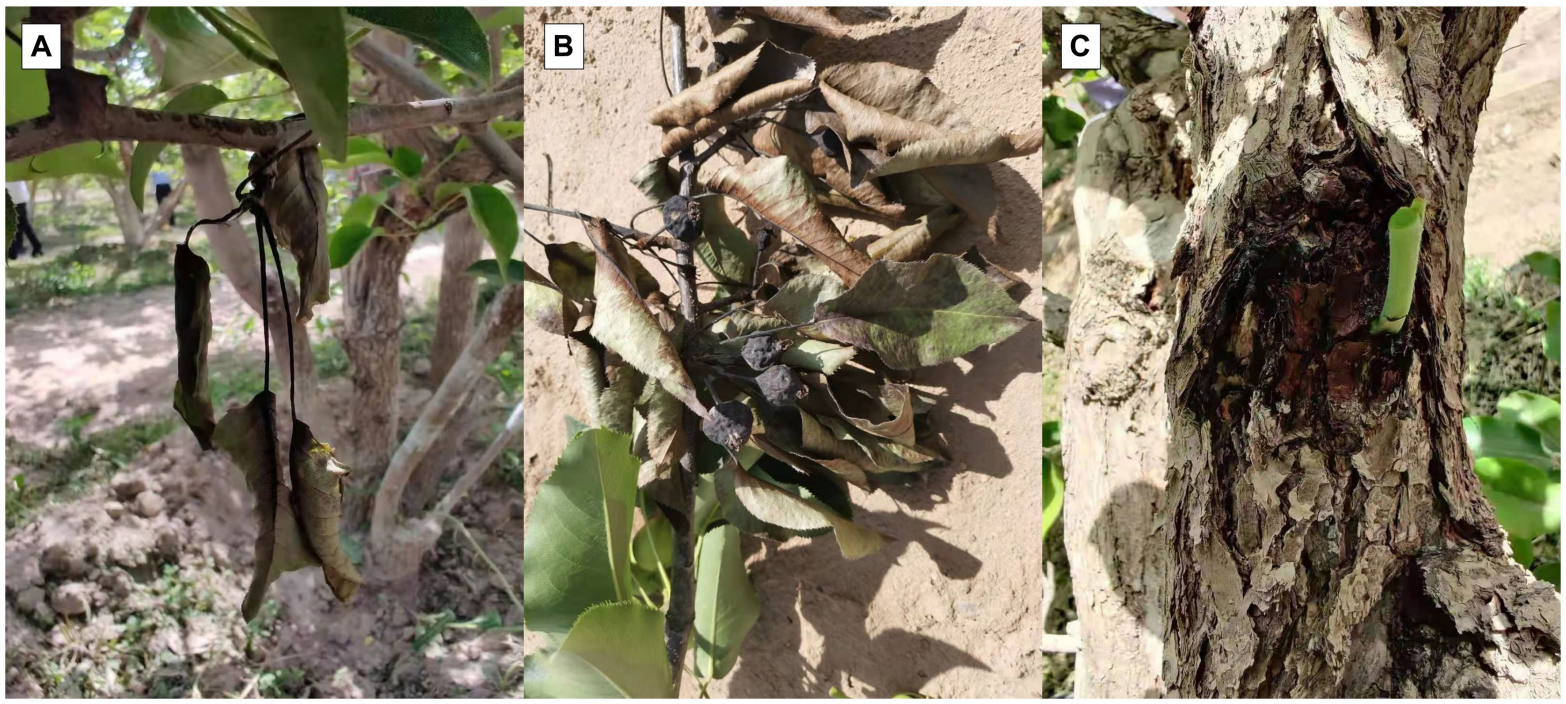
Field symptoms of novel disease similar to pear fire blight. **(A)** Disease spots on leaves. **(B)** Rotting black fruit. **(C)** Black spots on branches.

The main symptom of the disease was multiple disease spots on diseased leaves. The infected fruit turned black and completely rotted, withering and leading to a dead state. These caused the leaves to progressively change from yellow to a dark brown, withering but remaining attached to the branches. Black spots were produced in the phloem and xylem of infected branches, which expanded and produced bacterial pus (Figure 1). The symptoms were similar to those of pear fire blight.

### 3.2 Pathogenicity testing

**Figure 2:**
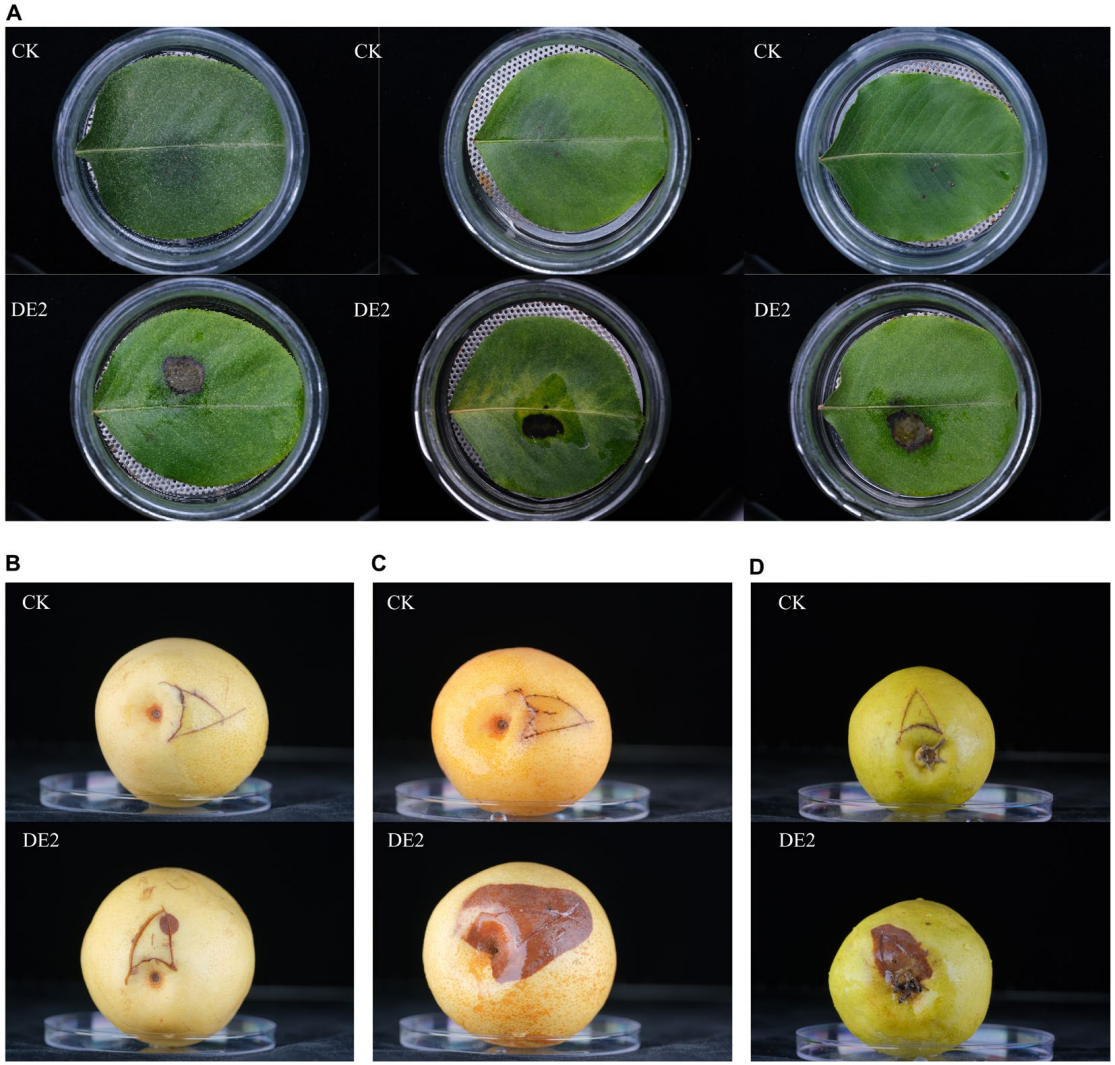
Pathogenicity testing of isolate DE2. **(A)** Leaves (*Pyrus betulaefolia* Bge.) inoculated with DE2, **(B)** fruits (*Pyrus pyrifolia* var. *Sinensis* (Lindley)Y. Teng et K.) inoculated with DE2, **(C)** fruits (*Pyrus ussuriensis* Maxim.) inoculated with DE2, **(D)** fruits (*Pyrus brestschneideri* Rehd.) inoculated with DE2. The symptoms of the leaves were observed on the 7th day after inoculation with DE2, and the symptoms of the fruits were observed on the 4th day. CK: Inoculate with sterile water instead of bacterial solution.

Based on the morphological characteristics (color, size, and shape) of the isolates from the diseased leaves, the isolates were classified into 8 types, coded DE1–DE8. It was then purified, cultured, and subjected to pathogenicity testing. The isolate of DE2 caused black lesions in detached leaves and fruits on day 7. It was noteworthy that DE2 showed different levels of pathogenicity after infecting pear fruits of different varieties. Among them, the symptoms of fruits (*Pyrus pyrifolia* var. *Sinensis* (Lindley)Y. Teng et K.) were the most severe, with the fastest infection rate and the largest area. However, the symptoms of fruits (*Pyrus ussuriensis* Maxim.) were the mildest. The symptoms of fruits (*Pyrus brestschneideri* Rehd.) were intermediate between the two, accompanied by the production of bacterial beads. This was consistent with the symptoms observed in the field. No disease symptoms were observed in the control group (Figure 2) and other seven isolates. The pathogenic bacteria were re-isolated from the diseased site of the detached leaves and fruits that had been inoculated with DE2, and the purified isolate was found to be identical to DE2. Thus, Koch’s postulates were verified, demonstrating that isolate DE2 was the disease pathogen.

### 3.3 Morphological characteristics of isolate DE2

**Figure 3:**
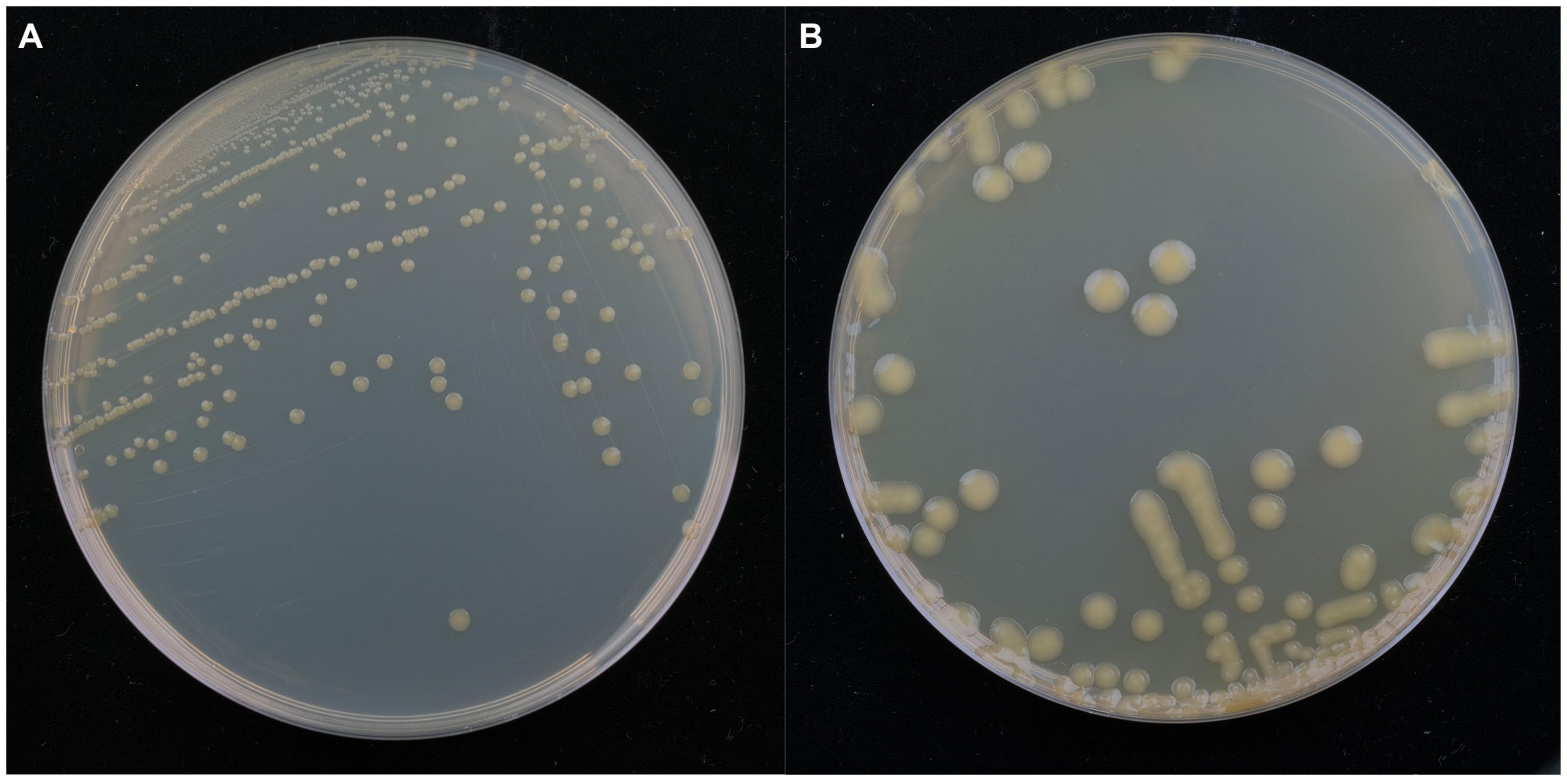
Morphological characteristics of isolate DE2 on NA medium **(A)** and TSA medium **(B)** after 48 h.

Isolate DE2 colonies on NA medium were initially milky white, while the later colonies were light yellow, sticky, dome-shaped, with a smooth surface and neat edges (Figure 3A). Isolate DE2 colonies on TSA medium were yellow (light yellow at the center, with a light yellow transparent ring at the periphery), with circular protrusions, a smooth, glossy surface, and neat edges (Figure 3B).

### 3.4 16S rRNA identification of isolate DE2

**Figure 4:**
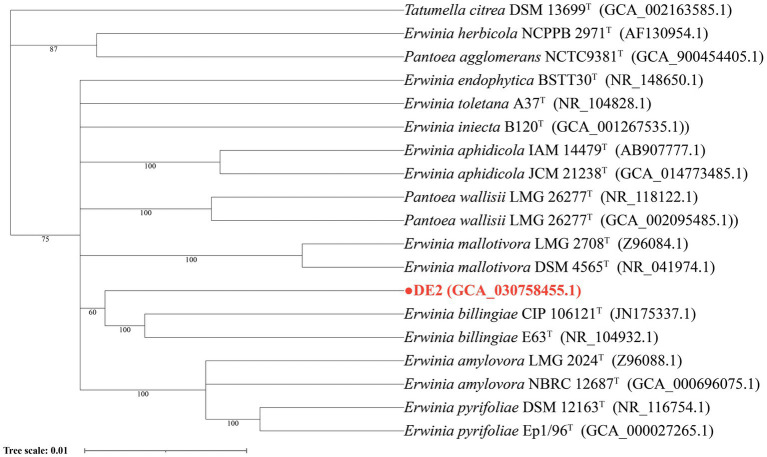
Phylogenetic tree of isolate DE2 based on the 16S rDNA sequence.

The phylogenetic tree based on 16S rRNA sequences showed that isolate DE2 was clustered with species in the genus *Erwinia* (Figure 4), indicating that the classification of isolate DE2 at the genus level is a species belonging to the genus *Erwinia*. The NCBI comparison results showed that the sequence similarity between isolate DE2 and strain *Erwinia billingiae* Eb661 was the highest, reaching 98.70% (Supplementary Table S2). The EZ BioCloud comparison results showed that the sequence similarity between isolate DE2 and strain *Erwinia billingiae* CIP 106121 was the highest, reaching 98.77% (Supplementary Table S3). Based on 16S rRNA analysis, the other isolates DE1, DE3-DE8 were all identified as *Bacillus* species (Supplementary Figure S1).

### 3.5 Physiological and biochemical characteristics of isolate DE2

According to the API 20E and GEN III results, isolate DE2 was negative for oxidase, and did not produce indole, urease, gelatinase, tryptophan dehydrogenase, arginine hydrolase, lysine decarboxylase, guanate decarboxylase, or β-galactosidase. Citric acid utilization and the Voges–Prokauer (VP) reaction were negative. Acid production could be achieved by utilizing glucose, mannitol, inositol, sorbitol, rhamnose, amygdalin, and arabinose. The carbon sources that could be utilized were α-D-glucose, D-sorbitol, D-mannose, D-mannitol, aminoacetyl-L-proline, D-galacturonic acid, methyl pyruvate, D-maltose, melibiose, D-fructose, D-arabitol, L-alanine, L-galacturonolactone, D-trehalose, β-formyl-D-glucoside, D-galactose, inositol, D-gluconic acid, L-lactic acid, D-cellobiose, D-salicylic acid, glycerol, L-aspartic acid, D-glucuronic acid, citric acid, N-acetyl-D-glucosamine, D-glucose-6-phosphate, L-glutamic acid, glucuronamide amide, α-Ketone glutaric acid, acetoacetic acid, N-acetyl-β-D-mannosamine, D-fructose-6-phosphate, L-histamine, mucic acid, L-rhamnose, quinic acid, L-malic acid, acetic acid, inosine, L-serine, gluconic acid, bromosuccinic acid, and formic acid. The carbon sources that could not be utilized were D-pine disaccharide, stachyose, trisaccharide α-D-lactose, L-arginine γ-aminobutyric acid, L-butyric acid, α-ketobutyric acid, and propionic acid. Isolate DE2 could be distinguished by 15 physiological indicators from its relatives, *Erwinia billingiae* Eb661, *Erwinia toletana* WS4403, *Pantoea wallisii* LMG 26277, and *Erwinia persicina* NBRC 102418 (Supplementary Table S4).

### 3.6 Fatty acid profile of isolate DE2

The main features of the fatty acid profile of isolate DE2 (Supplementary Figure S2) were as follows: C16:0 accounted for 56.01%, C18:1n9c for 10.86%, C22:1n9 for 10.26%, C16:1 for 6.26%, C18:2n6c for 6.21%, and C14:0 for 5.11% (Supplementary Table S5). Isolate DE2 could be distinguished from related species based on the cellular fatty acid profile (Supplementary Table S6).

### 3.7 Basic genomic characteristics of isolate DE2

**Figure 5:**
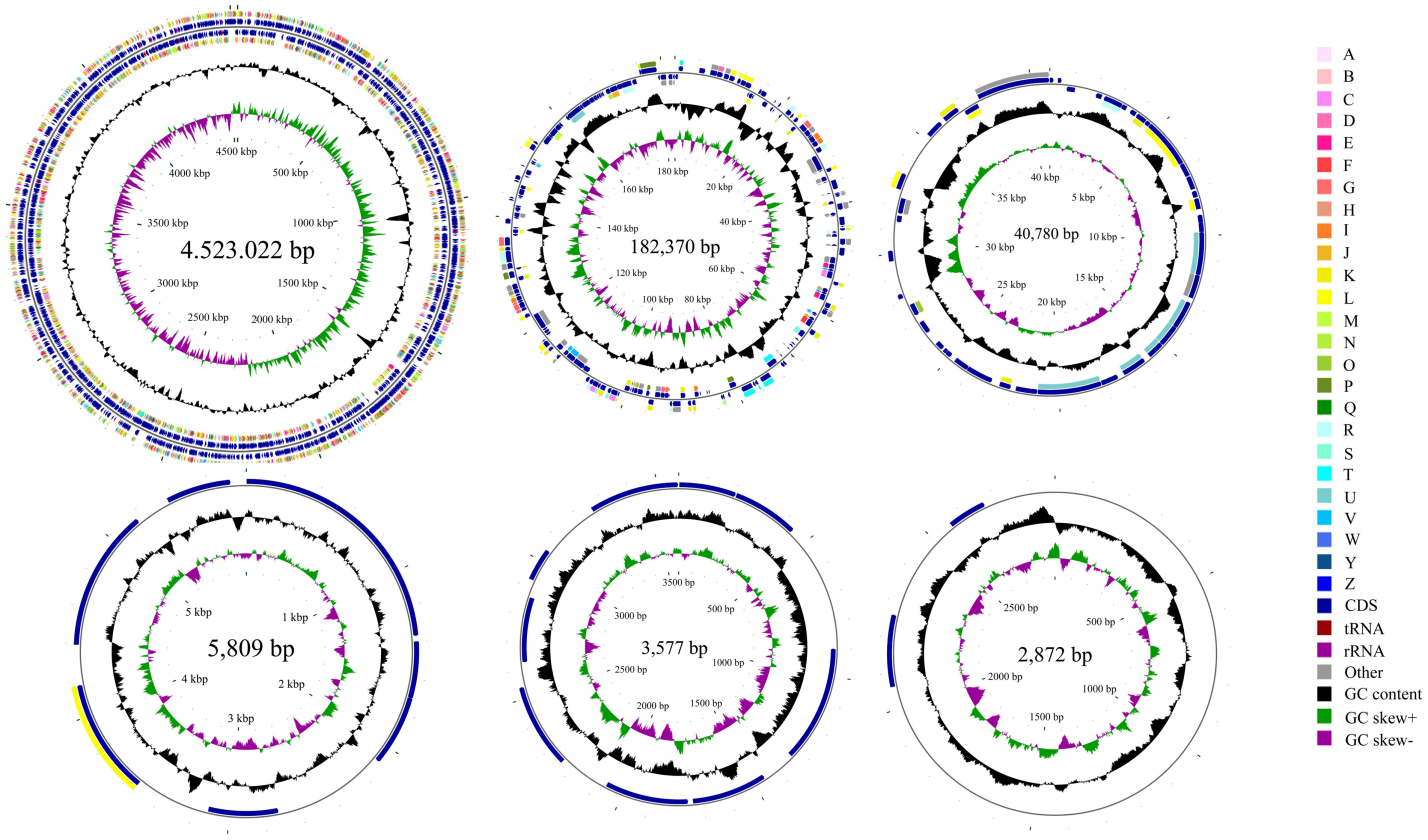
Circular map of the one chromosome and five plasmids in isolate DE2. The circles from the inside to outside represent. (1) GC skew. (2) GC content; (3) CDS on the reverse strand annotated using the Clusters of Orthologous Genes (COG) database; (4) CDS, rRNA, and tRNA on the reverse strand; (5) CDS, rRNA, and tRNA on the forward strand; (6) CDS on the forward strand; and (7) scale of genome size. The COG categories are A: RNA processing and modification; B: Chromatin structure and dynamics; C: Energy production and conversion; D: Cell cycle control, mitosis, and meiosis; E: Amino acid transport and metabolism; F: Nucleotide transport and metabolism; G: Carbohydrate transport and metabolism; H: Coenzyme transport and metabolism; I: Lipid transport and metabolism; J: Translation, ribosomal structure, and biogenesis; K: Transcription; L: Replication, recombination, and repair; M: Cell wall/membrane/envelope biogenesis; N: Cell motility; O: Post-translational modification, protein turnover and chaperones; P: Inorganic ion transport and metabolism; Q: Secondary metabolites biosynthesis, transport and catabolism; R: General function prediction only; S: Unknown function; T: Signal transduction mechanisms; U: Intracellular trafficking, secretion, and vesicular transport; V: Defense mechanisms; W: Extracellular structures; Y: Nuclear structure; Z: Cytoskeleton.

Whole-genome sequencing revealed that isolate DE2 contains 1 circular chromosome and 5 plasmids (Figure 5). The circular chromosome was 4,523,022 bp, with a GC content of 54.54%. Plasmid 1 was 182,370 bp, with a GC content of 50.02%. Plasmid 2 was 40,780 bp, with a GC content of 44.00%. Plasmid 3 was 5,809 bp, with a GC content of 54.30%. Plasmid 4 was 3,577 bp, with a GC content of 50.55%. Plasmid 5 was 2,872 bp, with a GC content of 46.20%. The genome coverage statistics for strain DE2 based on K-mer analysis stand at 92.51%. The coverage statistics derived from second-generation sequencing data aligning reads reach 99.30%, and those from the third-generation sequencing data aligning reads are 99.53%.

The circular chromosome contained 4,155 CDS, 77 tRNA, and 22 rRNA genes. Plasmid 1 contained 199 CDS. Plasmid 2 contained 43 CDS. Plasmid 3 contained 8 CDS. Plasmid 4 contained 9 CDS. Plasmid 5 contained 2 CDS (Supplementary Table S7).

As shown in the GC-depth distribution (Supplementary Figure S3), the upper and right histograms represent the sliding window frequency distributions of GC content and depth, respectively, showing that there was no obvious bias in the GC content of the sequencing sample; the multiple points in the middle were concentrated in a relatively small range, which indicated that there was no contamination of the DNA of isolate DE2.

### 3.8 Taxonomic classification of isolate DE2

**Figure 6:**
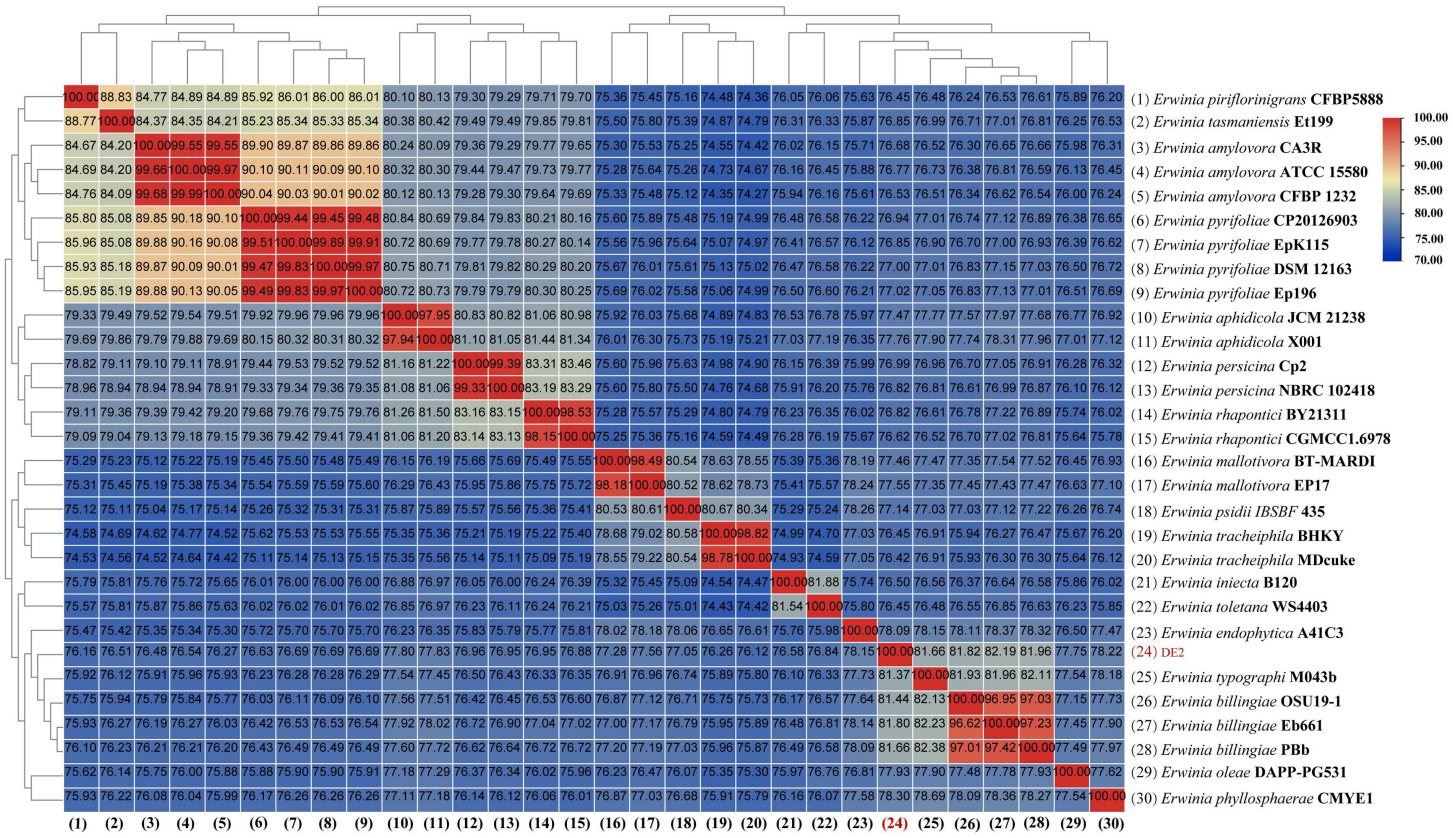
Heatmap of average nucleotide identification (ANI) based on the complete genome sequence of isolate DE2 and 29 strains in the genus *Erwinia*.

**Figure 7:**
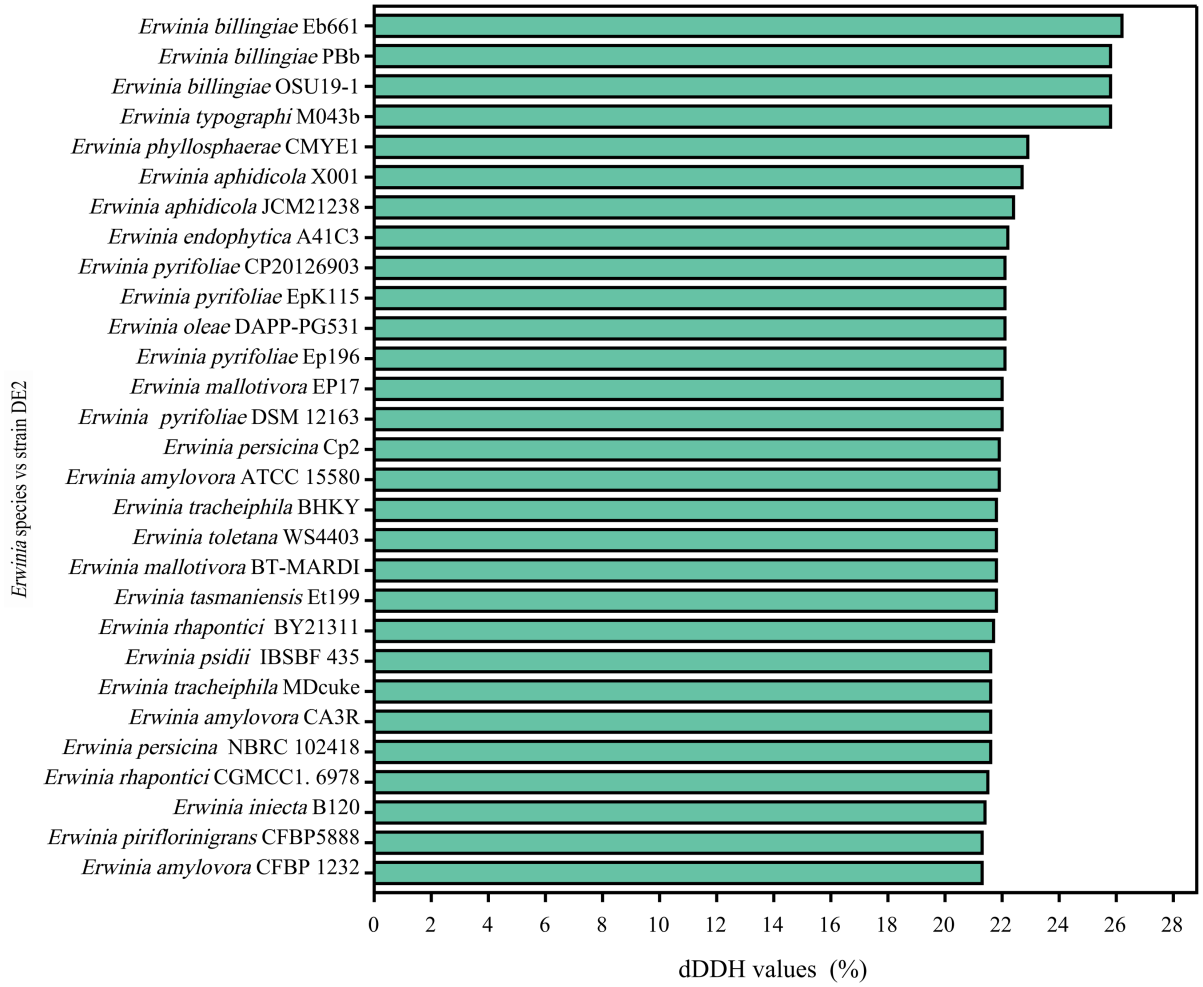
dDDH values between isolate DE2 and 29 *Erwinia* species.

dDDH and ANI are the widely accepted gold standards for identifying a bacterial isolate species. Through 16S rRNA gene sequencing, isolate DE2 was found to belong to the genus *Erwinia*. To clarify the taxonomic classification of isolate DE2, its complete genome was compared to the complete genomes of 29 reference strains of the genus *Erwinia* based on dDDH and ANI. The ANI values between isolate DE2 and the 29 *Erwinia* species were 76.42–81.80% (Figure 6). Similarly, the dDDH values were 21.3–26.2% (Figure 7). The ANI and dDDH thresholds for determining whether two strains are the same species are ≥95–96% and ≥70%, respectively. As the ANI and dDDH values between isolate DE2 and species of the genus *Erwinia* were lower than the threshold values, isolate DE2 is a potentially new species in the genus *Erwinia*.

### 3.9 Pan-genome analysis of strain DE2

**Figure 8:**
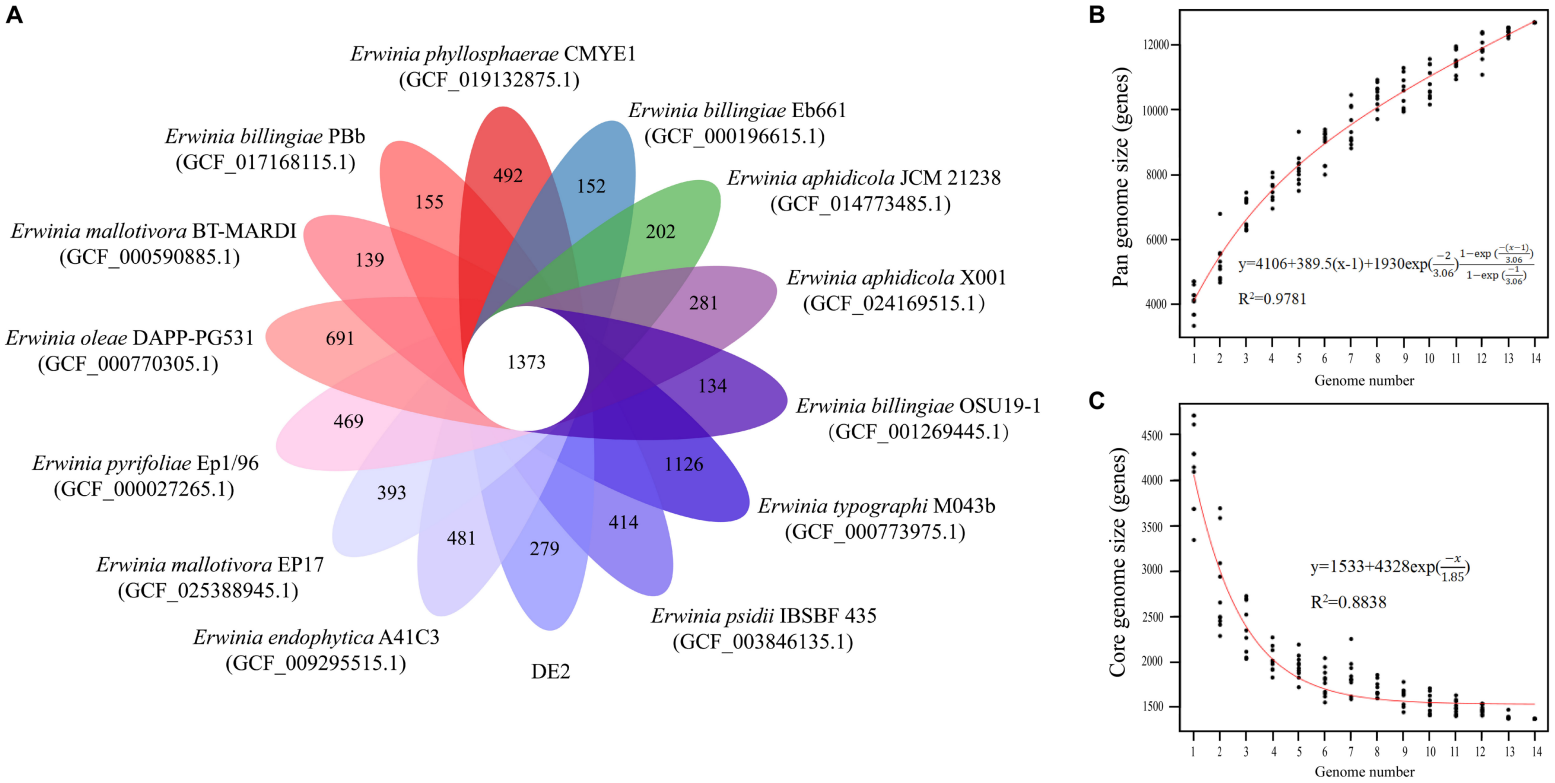
**(A)** Petal map of homologous genes among various species of the genus *Erwinia*. The core genes of 14 strains of *Erwinia* are located in the center, and there are strain specific genes in the petals. **(B)** Pangenomic maps between various species of the genus *Erwinia*. This curve is fitted by a power law regression model, representing the relationship between genome quantity and pan genome size. **(C)** The core genome map between various species of the genus *Erwinia*. Fit the exponential curve fitting model to represent the number of core genomes as a function of the number of sequentially added genomes.

The PGAP analysis showed that of the 6,781 genes in 10 *Erwinia* strains, 1,373 (20.25% of the pan-genome) were core genes shared by all 10. Each of the 10 strains had unique genes (134–1,126), and strain DE2 had 279 unique genes (Figure 8A). The equation for the relationship between the pan-genome size (y) and the number of genomes (x) is $y=4,106+389.5\;\left(x-1\right)+1,930\exp\left(\frac{-2}{3.06}\right)\frac{1-\exp\left(\frac{-\left(x-1\right)}{3.06}\right)}{1-\exp\left(\frac{-1}{3.06}\right)}$ (Figure 8B). The equation for the relationship between the number of core genes (y) and the number of genomes (x) is $y=1,533+4,328\exp\left(\frac{-x}{1.85}\right)$ (Figure 8C).

### 3.10 Potential virulence genes in strain DE2

**Figure 9:**
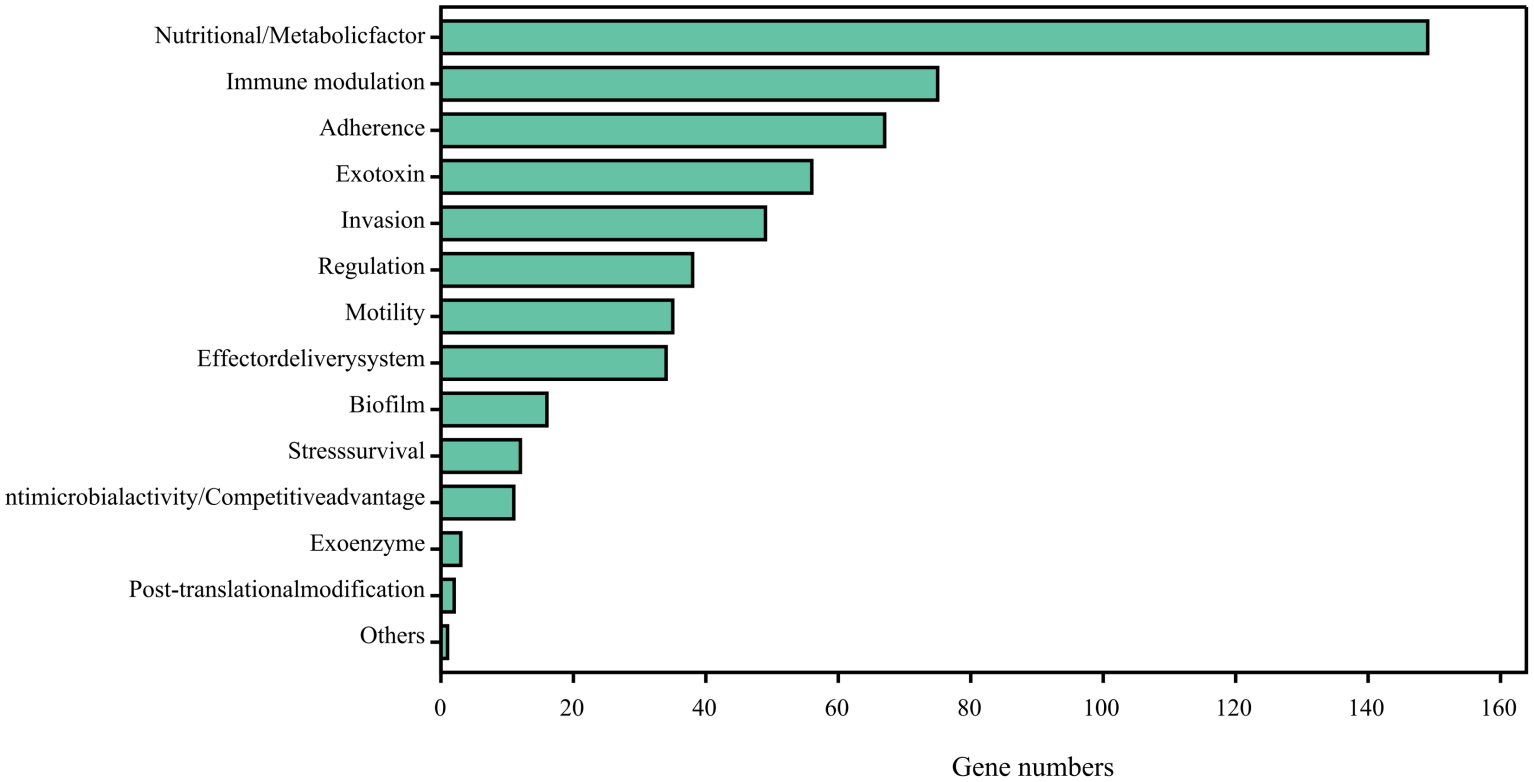
Potential virulence factors in strain DE2 genome predicted by VFDB database.

Comparing the complete genome sequence of strain DE2 with the VFDB database showed that strain DE2 has 548 putative virulence genes, comprising 534 on the chromosome and 14 in plasmids (Figure 9). These virulence genes can be divided into 14 categories, comprising Nutritional/metabolic factors (149 genes), Immune regulation (75 genes), Adhesion (67 genes), Exotoxins (56 genes), Invasion (49 genes), Regulation (39 genes), Mobility (35 genes), Effect delivery systems (34 genes), Biofilm (16 genes), Stress survival (12 genes), Antibiotic activity/competitive advantage (11 genes), Exoenzymes (3 genes), Post-translational modification (2 genes), and Others (1 gene) (Supplementary Table S8). Additionally, 1,064 genes were annotated using the Pathogen–Host Interactions (PHI)-base (Supplementary Table S9). Among them, plasmid 1 has 8 virulence factors, plasmid 2 has 6 virulence factors, and the remaining plasmids have no virulence factors.

### 3.11 Description of *Erwinia pyri* sp. nov.

*Erwinia pyri* (*pyri*. L. n. *pyrus betulaefolia* pear tree; L. n. *pyrus betulaefolia* leaves; L. part. Adj. blackened, darkened; N. L. part. Adj. *Pyri* withered pear leaves, from which the strain was isolated).

The strain is a Gram-negative, facultative aerobe that does not form spores. Oxidase reaction, citric acid utilization, and VP reaction are negative. Colonies grow on TSA medium after 48 h at 28°C; they are non-fluorescent, round, light yellow, slightly raised mucous colonies, 2–4 mm in diameter, with regular edges. Colonies grow on NA medium. Colonies grow in TSB nutrient solution at 28°C and 30°C but not at ≥32°C. Based on the API 20E system, indole, urease, gelatinase, tryptophan dehydrogenase, arginine hydrolase, lysine decarboxylase, β-galactosidase are negative, but acid production can be achieved by utilizing glucose, mannitol, inositol, sorbitol, rhamnose, melibiose, amygdalin, and arabinose. The main fatty acids were (in descending order): C16:0, C18:1n9c, C22:1n9, C16:1, C18:2n6c, C14:0, C18:0, C12:0, C20:3n3, C22:0, C18:3n3, C17:0, C24:1, C23:0, C24:0, C22:2, C20:0, C20:5n3, C20:1, C15:0, C13:0, and C22:6n3. The strain has a circular chromosome and five plasmids. The DNA G + C content is 54.54 mol%.

## 4 Discussion

Morphological analysis of strain DE2 colonies on NA medium showed that they were light yellow (with a deep yellow center and a light yellow transparent ring), round, smooth, and shiny. The morphological characteristics were similar to those of species in the genus *Panton* (Cruz et al., 2007). In contrast, the pathogenic bacteria *Erwinia amylovora*, responsible for pear fire blight, and *Erwinia pyrifoliae*, responsible for Asian pear fire blight, are both milky white on NA medium (Oh and Beer, 2005). It was noteworthy that only one yellow *Erwinia* strain, DE2, was identified in the isolates of this disease, and no white or other colored *Erwinia* strains were found. Therefore, it was ruled out that the disease was caused by *E. amylovora* and *E. pyrifoliae*. Based on our morphological results, the species of strain DE2 could not be determined. The 16S rRNA gene sequence analysis revealed that strain DE2 exhibited 97% similarity with various model bacteria in the genus *Erwinia*, which is slightly lower than the 16S rRNA similarity threshold of 98.65% for classification as a new species (Qian et al., 2019). Nonetheless, this threshold is only a reference for identifying potentially new bacterial species. The 16S rRNA gene sequence alone could not precisely classify strain DE2. Nevertheless, the phylogenetic tree based on 16S rRNA sequence alignment showed that strain DE2 could be classified at the genus level within the genus *Erwinia*. Furthermore, a comprehensive comparison of strain DE2’s complete genome sequence with species in the *Erwinia* genus revealed that the ANI values between strain DE2 and 29 *Erwinia* species were <95% and the dDDH values were <70%. Consequently, based on molecular analysis, whole genome sequencing, and phenotypic analysis, we propose that strain DE2 should be recognized as a new species in the genus *Erwinia*. The naming of new bacterial species adhered to the Linnaeus double naming system stipulated in the International Principles of Prokaryotic Nomenclature (Parker et al., 2015), which was “genus name+species name.” In this study, we suggested that strain DE2 be named “*Erwinia pyri* sp. nov.” The complete genome sequence of strain DE2 has been submitted to the NCBI database (ASM3075845v1) under accession number (GCA_030758455.1). The NCBI accession numbers of the whole genome of strain DE2, consisting of one chromosome and five plasmids, are NZ_CP132353.1, NZ_CP132354.1, NZ_CP132355.1, NZ_CP132356.1, NZ_CP132357.1, and NZ_CP132358.1. The strain DE2 was sent to the China Center for Type Culture Collection (CCTCC), and the obtained deposit number was CCTCC AB 2024080.

The pan-genome consists of the core and dispensable genomes. The core genome comprises genes shared by all individuals within a species/genus. It may not necessarily represent the minimal set of genes for survival in nature; rather, it serves as the foundational structure of the pan-genome (Koonin, 2003). In contrast, the dispensable genome comprises genes present in specific individuals. These genes are often associated with complementary biochemical pathways and functions that offer selective advantages, such as ecological adaptation, virulence mechanisms, antibiotic resistance, or colonization of new hosts. The concept of “pan-genome analysis” introduces a novel approach to species/genus definition. The first bacterial pan-genome was created based on *Streptococcus agalactiae*. According to Tettelin et al., approximately 30 new genes were discovered for each newly sequenced strain of *S. agalactiae*, leading to an expansion of the pan-genome (Tettelin et al., 2005). Consequently, *S. agalactiae* was classified as having an open pan-genome. With the continuous sequencing and assembly of individual genomes, the size of the pan-genome initially grew before gradually stabilizing. If no new genes are discovered, or if the number of genes decreases, a species/genus is categorized as having a closed pan-genome (Kumari et al., 2023). Based on the equations derived from pan-genome analysis of *Erwinia* species involving strain DE2, the pan-genome increased while the core genome decreased with increased sequencing of *Erwinia* species. Therefore, the pan-genome of *Erwinia* is currently open.

Bacteria not only pass their genome vertically to their offspring but can also acquire genetic material from the environment via horizontal transfer (Soucy et al., 2015). During gene acquisition, the bacterial genome typically undergoes replication or loss, in order to maintain its small and compact structural features (Tristan and Stanhope, 2007). A phylogenetic analysis of bacterial genomes is intricate due to the combined impacts of vertical and horizontal transmission (Puigbò et al., 2014). Notably, significant individual genome-level differences may occur within the same bacterial species. Core genes in the *Escherichia coli* pan-genome account for >10%. Even at the transcription factor level, notable distinctions can be observed among *E. coli* genomes (Cook and Ussery, 2013). Due to the substantial genetic variation and the need to reconstruct bacterial phylogenetic and population histories, pan-genome analysis is key and provides a strong basis for bacterial classification (Callaghan et al., 2015). An open pan-genome enables pathogenic bacteria to continuously acquire new genes related to resistance, virulence, or metabolism from the environment or bacterial population, thereby increasing their survival in the host. A pathogen’s genetic diversity poses substantial challenges to immunity and treatment (Freschi et al., 2019).

The pathogenicity of strain DE2 was evaluated by inoculation experiments on detached pear leaves. The disease symptoms induced by strain DE2 on the leaves were akin to those caused by *E. amylovora*, which is responsible for pear fire blight. However, the underlying mechanism was unclear. Bioinformatics analysis was used to predict the virulence factors of strain DE2. Strain DE2 had 45 virulence factor genes associated with the synthesis and motility of flagella (which was the most virulence gene category for strain DE2). These virulence factors contribute to biofilm formation and adaptation to host environments (Feldman et al., 1998; Dasgupta et al., 2010). There were few genes (compared to in other bacteria) dedicated to the virulence factor pyoverdine, which allows bacterial iron acquisition. It has been reported to be key for the aggregation of iron ions and virulence in bacteria (Cornelis, 2003; Xiao and Kisaalita, 2019). There were many more genes encoding the virulence factors FbpABC and HitABC (28 and 17, respectively). FbpABC and HitABC iron transport systems facilitate free iron transport into the cytoplasm of the pathogenic bacteria *Neisseria gonorrhoeae* and *Haemophilus influenzae*, making iron available in cells (Adhikari et al., 1995, 1996). There were 29 virulence factor genes related to lipooligosaccharide (LOS), which can mutate at high frequencies. Molecular simulations of LOS structures have provided clear evidence of the role of LOS in evading host defenses (Godschalk et al., 2004; Heikema et al., 2010). There were 10 virulence factor genes related to BfmRS, which controls biofilm formation and cell morphology and is related to cell adhesion, and antibiotic sensitivity (Tomaras et al., 2008; Liou et al., 2014). Other virulence factors found in strain DE2 included functions related to pathogenicity (Truswell, 1978; Lee et al., 2010). such as lysing macrophages, mediating chronic bacterial infections, penetrating the host defense system, and injecting toxins into the host cytoplasm (Lyczak et al., 2000; Skerker and Berg, 2001; Suarez et al., 2008).

## 5 Conclusion

A light-yellow bacterial strain, designated DE2, was isolated from pear leaves affected by a novel disease similar to pear fire blight. Validation of Koch’s postulates confirmed it as the pathogen that causes the disease. Morphological methods, 16S rRNA sequence alignment, and ANI and dDDH analyses indicated that strain DE2 belonged to a potentially new species in the genus *Erwinia*. Based on the international naming convention, we will temporarily name strain DE2 as *Erwinia pyri* sp. nov. Examination of the complete genome of strain DE2 unveiled the existence of 548 virulence factors, grouped into 14 categories. Thus, this study revealed a potentially new species of pathogenic bacteria in the genus *Erwinia* that is responsible for the occurrence of a bacterial dieback disease in pear trees, a condition with similarities to pear fire blight. This study enriches the *Erwinia* strain resources, providing a theoretical basis for future research, effective prevention, and control of the disease.

## Data availability statement

The datasets presented in this study can be found in online repositories. The names of the repository/repositories and accession number(s) can be found in the article/Supplementary material.

## Author contributions

LH: Conceptualization, Data curation, Formal analysis, Investigation, Methodology, Resources, Software, Supervision, Validation, Visualization, Writing – original draft, Writing – review & editing. RH: Conceptualization, Investigation, Software, Writing – review & editing. HC: Data curation, Methodology, Supervision, Writing – review & editing. LZ: Formal analysis, Validation, Visualization, Writing – review & editing. ZZ: Methodology, Project administration, Resources, Writing – review & editing.

## Footnotes

^1^ https://blast.ncbi.nlm.nih.gov/Blast.cgi

^2^ https://help.ezbiocloud.net/

^3^ http://cloud.majorbio.com

^4^ https://cgview.ca/

^5^ http://jspecies.ribohost.com/jspeciesws/analyse

^6^ https://ggdc.dsmz.de/ggdc.php

^7^ https://www.ncbi.nlm.nih.gov/datasets/taxonomy/551/

^8^ https://cloud.majorbio.com/page/tools/tools.html?cmd_id=1khv95pqclo8ju9lc7htuhnf1c&v=2

^9^ http://www.mgc.ac.cn/VFs/

## Supplementary material

Supplementary Figure S1 Phylogenetic tree of isolate DE1, DE3-DE8 based on the 16S rDNA sequence.

Supplementary Figure S2 Fatty acid profile of strain DE2.

Supplementary Figure S3 GC-depth distribution of strain DE2. GC content and mean depth with a **(A)** 1-, **(B)** 3-, **(C)** 5-, **(D)** 8-, and **(E)** 10-kb windows.
