# Supplementary material for: Discovery and characterization of a novel pathogen Erwinia pyri sp. nov. associated with pear dieback: taxonomic insights and genomic analysis
Source: Front Microbiol. 2024 May 9;15:1365685. doi: 10.3389/fmicb.2024.1365685 (PMC11111954; doi:10.3389/fmicb.2024.1365685)
Supplement: Supplementary file 1 [file Table_1.DOCX]

| **TABLE S1 \|** Amplification of 16S rRNA gene | |
| --- | --- |
| Reagent | Volume（μL) |
| 2×Tap PCR Mix | 25.0 |
| DNA template | 2.0 |
| Primer 27F | 2.0 |
| Primer 1492R | 2.0 |
| ddH2O2 | 19.0 |
