# Supplementary material for: Discovery and characterization of a novel pathogen Erwinia pyri sp. nov. associated with pear dieback: taxonomic insights and genomic analysis
Source: Front Microbiol. 2024 May 9;15:1365685. doi: 10.3389/fmicb.2024.1365685 (PMC11111954; doi:10.3389/fmicb.2024.1365685)
Supplement: Supplementary file 2 [file Table_2.DOCX]

| **TABLE S2** \| Classification and identification results of strain DE2 based on 16S rRNA homology analysis in NCBI | | |
| --- | --- | --- |
| Closest Type Strain | GenBank accession No. | Similarity (%) |
| *Erwinia billingiae* Eb661 | GCF_000196615.1 | 98.700 |
| *Erwinia* sp. E602 | GCF_018141005.1 | 98.376 |
| *Erwinia toletana* WS4403 | GCF_017875465.1 | 98.246 |
| *Pantoea wallisii* LMG 26277 | GCF_002095485.1 | 98.247 |
| *Erwinia persicina* NBRC 102418 | GCF_001571305.1 | 98.182 |
| *Erwinia aphidicola* JCM 21238 | GCF_014773485.1 | 98.052 |
| *Erwinia* sp. IMH | GCF_000599885.1 | 97.726 |
| *Phytobacter ursingii* ATCC27989 | GCA_901456055.1 | 97.531 |
| *Pantoea eucrina* LMG 5346 | GCF_002095385.1 | 97.403 |
| *Pantoea rwandensis* LMG 26275 | GCF_002095475.1 | 97.271 |
| *Erwinia* sp. J780 | GCF_009738185.1 | 97.271 |
| *Enterobacterales bacterium* CwR94 | GCA_002837195.1 | 97.206 |
| *Citrobacter* sp. R56 | GCF_016811995.1 | 97.076 |
| *Enterobacter asburiae* E. a101 | GCF_011754535.1 | 97.076 |
| *Leclercia adecarboxylata* Z96-1 | GCF_006171285.1 | 97.011 |
| *Erwinia beijingensis* LMG 27579 | GCF_004022165.1 | 96.948 |
| *Pantoea cypripedii* LMG 2657 | GCF_002095535.1 | 96.946 |
| *Klebsiella africana* SB5857 | GCF_900978845.1 | 96.946 |
| *Klebsiella pneumoniae* ATCC 13883 | GCF_000742135.1 | 96.948 |
