# Supplementary material for: Discovery and characterization of a novel pathogen Erwinia pyri sp. nov. associated with pear dieback: taxonomic insights and genomic analysis
Source: Front Microbiol. 2024 May 9;15:1365685. doi: 10.3389/fmicb.2024.1365685 (PMC11111954; doi:10.3389/fmicb.2024.1365685)
Supplement: Supplementary file 3 [file Table_3.DOCX]

| **TABLE S3** \| Classification and identification results of strain DE2 based on 16S rRNA homology analysis in EZ BioCloud | | |
| --- | --- | --- |
| **Closest Type Strain** | **Accession No.** | **Similarity (%)** |
| *Erwinia billingiae* CIP 106121 | JN175337 | 98.77 |
| *Erwinia endophytica* BSTT30 | LN624761 | 98.41 |
| *Erwinia persicina* NBRC 102418 | BCTN01000053 | 98.29 |
| *Erwinia toletana* CECT 5263 | FR870447 | 98.27 |
| *Pantoea wallisii* LMG 26277 | MLFS01000124 | 98.22 |
| *Erwinia aphidicola* JCM 21238 | JACXBP010000023 | 98.15 |
| *Winslowiella arboricola* BAC 15a-03b | OP422451 | 97.99 |
| *Erwinia tasmaniensis* Et 1/99 | CU468135 | 97.95 |
| *Erwinia rhapontici* ATCC 29283 | U80206 | 97.85 |
| *Pantoea dispersa* LMG 2603 | DQ504305 | 97.84 |
| *Pantoea coffeiphila* Ca04 | KJ427829 | 97.78 |
