# Supplementary material for: Discovery and characterization of a novel pathogen Erwinia pyri sp. nov. associated with pear dieback: taxonomic insights and genomic analysis
Source: Front Microbiol. 2024 May 9;15:1365685. doi: 10.3389/fmicb.2024.1365685 (PMC11111954; doi:10.3389/fmicb.2024.1365685)
Supplement: Supplementary file 4 [file Table_4.DOCX]

| **TABLE S4** \| Characteristics that distinguish the strain DE2 from closest *Erwinia* species | | | | | |
| --- | --- | --- | --- | --- | --- |
| Characteristic | DE2 | *Erwinia billingiae* Eb661 | *Erwinia_toletana* WS4403 | *Pantoea_wallisii* LMG 26277 | *Erwinia_persicina* NBRC 102418 |
| Acetone production | – | + | – | NO | + |
| Citrate utilization | – | – | – | + | NO |
| Nitrate reduced to nitrite | – | + | – | NO | NO |
| Acid production from: |  |  |  |  |  |
| Cellobiose | + | – | NO | + | + |
| Lactose | + | – | + | – | + |
| Melibiose | + | – | + | + | + |
| D-Turanose | – | – | – | NO | + |
| Gentiobiose | – | + | – | + | + |
| Raffinose | – | – | – | – | + |
| D- Ara bi to1 | + | + | + | + | – |
| Amygdalin | + | – | + | NO | + |
| Arbutin | – | + | – | NO | + |
| D-Gluconate | + | + | – | – | NO |
| 5-Keto-D-gluconate | – | + | – | – | NO |
| N-Acetylglucosamine | + | + | + | NO | + |

Note:+, positive; -, Negative; NO, unknown.
