# Supplementary material for: Discovery and characterization of a novel pathogen Erwinia pyri sp. nov. associated with pear dieback: taxonomic insights and genomic analysis
Source: Front Microbiol. 2024 May 9;15:1365685. doi: 10.3389/fmicb.2024.1365685 (PMC11111954; doi:10.3389/fmicb.2024.1365685)
Supplement: Supplementary file 5 [file Table_5.DOCX]

| **TABLE S5** \| Fatty acid Content of the strain DE2 | |
| --- | --- |
| **Fatty acid** | **Content（g/100g）** |
| C4:0 | - |
| C6:0 | - |
| C8:0 | - |
| C10:0 | - |
| C11:0 | - |
| C12:0 | 0.0155 |
| C13:0 | 0.0001 |
| C14:0 | 0.0511 |
| C14:1 | - |
| C15:0 | 0.0006 |
| C15:1 | - |
| C16:0 | 0.5601 |
| C16:1 | 0.0626 |
| C17:0 | 0.0018 |
| C17:1 | - |
| C18:0 | 0.0214 |
| C18:1n9 | - |
| C18:1n9c | 0.1086 |
| C18:2n6t | - |
| C18:2n6c | 0.0621 |
| C20:0 | 0.0009 |
| C18:3n6 | - |
| C18:3n3 | 0.0025 |
| C20:1 | 0.0008 |
| C21:0 | - |
| C20:2 | - |
| C22:0 | 0.0034 |
| C20:3n6 | - |
| C20:3n3 | 0.0063 |
| C22:1n9 | 0.1026 |
| C20:4n6 | - |
| C23:0 | 0.0013 |
| C22:2 | 0.0011 |
| C20:5n3 | 0.0009 |
| C24:0 | 0.0012 |
| C24:1 | 0.0016 |
| C22:6n3 | 0.0001 |
