# Supplementary material for: Discovery and characterization of a novel pathogen Erwinia pyri sp. nov. associated with pear dieback: taxonomic insights and genomic analysis
Source: Front Microbiol. 2024 May 9;15:1365685. doi: 10.3389/fmicb.2024.1365685 (PMC11111954; doi:10.3389/fmicb.2024.1365685)
Supplement: Supplementary file 6 [file Table_6.DOCX]

| **TABLE S6** \| Cellular fatty acid profile of strain DE2 and related species | | | |
| --- | --- | --- | --- |
| **Fatty acid** | **DE2** | ***Erwinia billingiae* Eb661** | ***Pantoea_wallisii* LMG 26277** |
| C12:0 | 1.55% | 3.68% | 4.2% |
| C14:0 | 5.11% | 5.81% | 6.9% |
| C15:0 | 0.6% | - | - |
| C16:0 | 56.01% | 31.26% | 26.1% |
| C16:1 | 6.26% | 22.15% | - |
| C17:0 | 1.8% | 3.18% | 7.1% |
| C18:0 | 2.14% | 1.52% | - |

Note: The measurement unit of fatty acid profile was g/100g.
