# Supplementary material for: Discovery and characterization of a novel pathogen Erwinia pyri sp. nov. associated with pear dieback: taxonomic insights and genomic analysis
Source: Front Microbiol. 2024 May 9;15:1365685. doi: 10.3389/fmicb.2024.1365685 (PMC11111954; doi:10.3389/fmicb.2024.1365685)
Supplement: Supplementary file 7 [file Table_7.DOCX]

| **TABLE S7** \| General genome features of the strain DE2 | | | | | | |
| --- | --- | --- | --- | --- | --- | --- |
| **Feature** | **Size (bp)** | **GC content (%)** | **CDS** | **tRNAs** | **rRNAs** | **sRNAs** |
| Chromosome | 4523022 | 54.54 | 4155 | 77 | 22 | 94 |
| Plasmid 1 | 182370 | 50.02 | 199 | 0 | 0 | 0 |
| Plasmid 2 | 40780 | 44.00 | 43 | 0 | 0 | 0 |
| Plasmid 3 | 5809 | 54.30 | 8 | 0 | 0 | 0 |
| Plasmid 4 | 3577 | 50.55 | 9 | 0 | 0 | 0 |
| Plasmid 5 | 2872 | 46.20 | 2 | 0 | 0 | 0 |
