# Supplementary material for: Discovery and characterization of a novel pathogen Erwinia pyri sp. nov. associated with pear dieback: taxonomic insights and genomic analysis
Source: Front Microbiol. 2024 May 9;15:1365685. doi: 10.3389/fmicb.2024.1365685 (PMC11111954; doi:10.3389/fmicb.2024.1365685)
Supplement: Supplementary file 8 [file Table_8.DOCX]

| **TABLE S8** \| Prediction of virulence genes of the strain DE2 in VFDB | | | | | |
| --- | --- | --- | --- | --- | --- |
| **virulence factors category** | **Specific virulence factors** | **numbers** | | | |
| Adherence | Type IV pili | 13 | | | |
|  | Trw type IV secretion system | 1 | | | |
|  | TCP | 1 | | | |
|  | Tap type IV pili | 2 | | | |
|  | S fimbriae | 1 | | | |
|  | Polar flagella | 25 | | | |
|  | P5 protein | 1 | | | |
|  | P fimbriae | 1 | | | |
|  | MSHA pili | 1 | | | |
|  | MAM7 | 1 | | | |
|  | LPS | 1 | | | |
|  | LOS | 1 | | | |
|  | Lap | 4 | | | |
|  | IlpA | 1 | | | |
|  | Hsp60 | 1 | | | |
|  | Flagella | 3 | | | |
|  | FHA | 1 | | | |
|  | EF-Tu | 2 | | | |
|  | Capsule | 4 | | | |
|  | CadF | 2 | | | |
| Antimicrobial activity/Competitive advantage | AcrAB | 5 | | | |
|  | FarAB | 6 | | | |
| Biofilm | BopD | 5 | | | |
|  | Csu fimbriae | 2 | | | |
|  | AdeFGH efflux pump | 8 | | | |
|  | Quorum-sensing | 1 | | | |
| Effector delivery system | RicA | 1 | | | |
|  | T4SS effectors | 4 | | | |
|  | T6SS | 8 | | | |
|  | Dot/Icm | 2 | | | |
|  | HSI-I | 4 | | | |
|  | TTSS | 6 | | | |
|  | T3SS1 | 3 | | | |
|  | Exe T2SS | 1 | | | |
|  | T3SS | 1 | | | |
|  | Rvh T4SS | 1 | | | |
|  | VirB type IV secretion system | 3 | | | |
| Exoenzyme | TlyC | 2 | | | |
|  | Aureolysin | 1 | | | |
| Exotoxin | The repeat in toxin | | 8 | | |
|  | BopD | | 7 | | |
|  | Phospholipase D | | 1 | | |
|  | alpha-Hemolysin | | 5 | | |
|  | Beta-hemolysin/cytolysin | | 14 | | |
|  | Hemolysin III | | 1 | | |
|  | Colibactin | | 7 | | |
|  | Exolysin | | 2 | | |
|  | Cya | | 3 | | |
|  | Hemolysin | | 3 | | |
|  | RTX toxin | | 2 | | |
|  | Cytolysin | | 1 | | |
|  | Hemolysin, HlyA | | 1 | | |
|  | Ptx | | 1 | | |
| Immune modulation | Alginate regulation | | 4 | | |
|  | Capsule | | 22 | | |
|  | Capsule I | | 2 | | |
|  | Alginate | | 7 | | |
|  | LOS | | 29 | | |
|  | OatA | | 1 | | |
|  | O-antigen | | 1 | | |
|  | LPS | | 8 | | |
|  | Rck | | 1 | | |
| Invasion | Invasin B/Ifp | | 1 | | |
|  | Flagella | | 45 | | |
|  | Bsa T3SS | | 1 | | |
|  | TTSS | | 1 | | |
|  | OmpA | | 1 | | |
| Motility | peritrichous flagella | | 24 | | |
|  | polar flagella | | 4 | | |
|  | Pse5Ac7Ac | | 1 | | |
|  | Pse5Ac7Ac, Pse5Ac7Am, Pse8OAc, Pse5Am7AcGlnAc | | 3 | | |
|  | Flagella | | 2 | | |
|  | Lateral flagella | | 1 | | |
| Nutritional/Metabolic factor | Acinetobactin | | 7 | | |
|  | Allantio+E348:E496n utilization | | 1 | | |
|  | Allantion utilization | | 7 | | |
|  | amonabactin | | 1 | | |
|  | Biotin synthesis | | | 3 | |
|  | CcmC | | | 1 | |
|  | Chu | | | 3 | |
|  | Cytochrome c maturation locus | | | 5 | |
|  | Ent | | | 5 | |
|  | Enterobactin | | | 3 | |
|  | FbpABC | | | 28 | |
|  | FeoAB | | | 1 | |
|  | FupA | | | 1 | |
|  | GGT | | | 2 | |
|  | HitABC | | | 17 | |
|  | Legiobactin | | | 2 | |
|  | MgtBC | | | 3 | |
|  | Mycobactin | | | 2 | |
|  | Phenazines biosynthesis | | | 3 | |
|  | Purine biosynthesis | | | 1 | |
|  | Pyochelin | | | 4 | |
|  | pyoverdine | | | 33 | |
|  | Pyrimidine biosynthesis | | | 4 | |
|  | Rhizoferrin | | | 1 | |
|  | Sal | | | 3 | |
|  | Salmochelin siderophore | | | 3 | |
|  | Shu | | | 3 | |
|  | Ybt | | | 2 | |
| Post-translational modification | Mip | | | 1 | |
|  | PrsA2 | | | 1 | |
| Regulation | CdpA | | | 11 | |
|  | BfmRS | | | 10 | |
|  | PhoP/R | | | 6 | |
|  | PhoP | | | 4 | |
|  | RelA | | | 2 | |
|  | AI-2 | | | 1 | |
|  | RcsAB | | | 3 | |
|  | Quorom sensing | | | 1 | |
| Stress survival | SodB | | | 1 | |
|  | KatAB | | | 1 | |
|  | ClpC | | | 2 | |
|  | RecN | | | 1 | |
|  | KatA | | | 1 | |
|  | SodCI | | | | 1 |
|  | MsrAB | | | | 1 |
|  | MntABC | | | | 3 |
|  | ClpP | | | | 1 |
| Others | Isocitrate lyase | | | | 1 |
