# Supplementary material for: Discovery and characterization of a novel pathogen Erwinia pyri sp. nov. associated with pear dieback: taxonomic insights and genomic analysis
Source: Front Microbiol. 2024 May 9;15:1365685. doi: 10.3389/fmicb.2024.1365685 (PMC11111954; doi:10.3389/fmicb.2024.1365685)
Supplement: Supplementary file 9 [file Table_9.DOCX]

| **TABLE S9** \| Identification of pathogenicity genes through interrogation of the strain DE2 genome with the PHI-base | | | | | |
| --- | --- | --- | --- | --- | --- |
| **Gene ID** | **Location** | **Hit Gene Name** | **PHI ID** | **Pathogen Species** | **Mutant Phenotype** |
| gene0015 | Chromosome | ibpA | PHI:4102 | Brucella suis | unaffected pathogenicity |
| gene0022 | Chromosome | MopB | PHI:7287 | Xylella fastidiosa | reduced virulence |
| gene0031 | Chromosome | argD | PHI:3126 | Erwinia amylovora | unaffected pathogenicity |
| gene0032 | Chromosome | SOD2 | PHI:420 | Cryptococcus gattii vgiii | loss of pathogenicity |
| gene0035 | Chromosome | potA | PHI:6319 | Streptococcus pneumoniae | reduced virulence |
| gene0036 | Chromosome | VK055_1939 | PHI:7533 | Klebsiella pneumoniae | unaffected pathogenicity |
| gene0040 | Chromosome | Mpd1 | PHI:2249 | Parastagonospora nodorum | unaffected pathogenicity |
| gene0047 | Chromosome | App | PHI:4612 | Clostridioides difficile | increased virulence (hypervirulence) |
| gene0049 | Chromosome | oppF (bb0335) | PHI:7821 | Borreliella burgdorferi | reduced virulence |
| gene0050 | Chromosome | oppD (bb0334) | PHI:7820 | Borreliella burgdorferi | reduced virulence |
| gene0051 | Chromosome | pdhR | PHI:3135 | Yersinia pseudotuberculosis | reduced virulence |
| gene0061 | Chromosome | cab (NMB0789) | PHI:8623 | Neisseria meningitidis | unaffected pathogenicity |
| gene0070 | Chromosome | vraR | PHI:7986 | Streptococcus suis | reduced virulence |
| gene0071 | Chromosome | Gph1 | PHI:2062 | Magnaporthe oryzae | unaffected pathogenicity |
| gene0079 | Chromosome | potA | PHI:6319 | Streptococcus pneumoniae | reduced virulence |
| gene0090 | Chromosome | ERG10 | PHI:2529 | Aspergillus fumigatus | lethal |
| gene0095 | Chromosome | XC_2801 | PHI:3260 | Xanthomonas campestris | reduced virulence |
| gene0098 | Chromosome | leuC | PHI:9329 | Erwinia amylovora | reduced virulence |
| gene0099 | Chromosome | Fgleu1 (FGSG_09589) | PHI:9357 | Fusarium graminearum | loss of pathogenicity |
| gene0112 | Chromosome | PhzF1 | PHI:7297 | Pseudomonas aeruginosa | reduced virulence |
| gene0119 | Chromosome | XC_2659 | PHI:9170 | Xanthomonas campestris | unaffected pathogenicity |
| gene0123 | Chromosome | murQ | PHI:7350 | Staphylococcus aureus | unaffected pathogenicity |
| gene0127 | Chromosome | ohrR | PHI:5549 | Vibrio cholerae | reduced virulence |
| gene0134 | Chromosome | App | PHI:4612 | Clostridioides difficile | increased virulence (hypervirulence) |
| gene0136 | Chromosome | oppD (bb0334) | PHI:7820 | Borreliella burgdorferi | reduced virulence |
| gene0137 | Chromosome | oppF (bb0335) | PHI:7821 | Borreliella burgdorferi | reduced virulence |
| gene0140 | Chromosome | bcsA | PHI:4694 | Salmonella enterica | increased virulence (hypervirulence) |
| gene0141 | Chromosome | bcsA | PHI:4694 | Salmonella enterica | increased virulence (hypervirulence) |
| gene0151 | Chromosome | bcsA | PHI:4694 | Salmonella enterica | increased virulence (hypervirulence) |
| gene0154 | Chromosome | pdeC | PHI:8644 | Erwinia amylovora | unaffected pathogenicity |
| gene0156 | Chromosome | ypo3991 | PHI:4189 | Yersinia pestis | reduced virulence |
| gene0165 | Chromosome | yebN | PHI:7899 | Xanthomonas oryzae | reduced virulence |
| gene0166 | Chromosome | THR1 | PHI:7696 | Sclerotinia sclerotiorum | unaffected pathogenicity |
| gene0167 | Chromosome | dnaJ | PHI:4733 | Francisella tularensis | unaffected pathogenicity |
| gene0173 | Chromosome | fabG1 | PHI:5271 | Ralstonia solanacearum | lethal |
| gene0189 | Chromosome | oppF (bb0335) | PHI:7821 | Borreliella burgdorferi | reduced virulence |
| gene0191 | Chromosome | App | PHI:4612 | Clostridioides difficile | increased virulence (hypervirulence) |
| gene0198 | Chromosome | oppF (bb0335) | PHI:7821 | Borreliella burgdorferi | reduced virulence |
| gene0199 | Chromosome | oppD (bb0334) | PHI:7820 | Borreliella burgdorferi | reduced virulence |
| gene0201 | Chromosome | App | PHI:4612 | Clostridioides difficile | increased virulence (hypervirulence) |
| gene0204 | Chromosome | metR | PHI:9326 | Erwinia amylovora | unaffected pathogenicity |
| gene0208 | Chromosome | Xdh1 | PHI:2256 | Parastagonospora nodorum | unaffected pathogenicity |
| gene0211 | Chromosome | ovrB (Z0346) | PHI:9508 | Escherichia coli | reduced virulence |
| gene0221 | Chromosome | BfiS | PHI:6998 | Pseudomonas aeruginosa | increased virulence (hypervirulence) |
| gene0222 | Chromosome | PXO_RS10155 | PHI:8980 | Xanthomonas oryzae | unaffected pathogenicity |
| gene0224 | Chromosome | kdpB | PHI:4712 | Pseudomonas aeruginosa | reduced virulence |
| gene0226 | Chromosome | kdpD | PHI:6313 | Pseudomonas syringae | reduced virulence |
| gene0229 | Chromosome | potA | PHI:6319 | Streptococcus pneumoniae | reduced virulence |
| gene0236 | Chromosome | LmABCB3 | PHI:5554 | Leishmania major | reduced virulence |
| gene0240 | Chromosome | MarA | PHI:5369 | Salmonella enterica | reduced virulence |
| gene0244 | Chromosome | fecE | PHI:8010 | Escherichia coli | reduced virulence |
| gene0248 | Chromosome | tsr | PHI:6981 | Salmonella enterica | reduced virulence |
| gene0249 | Chromosome | Glr1 | PHI:4904 | Candida albicans | reduced virulence |
| gene0257 | Chromosome | PitA | PHI:9451 | Staphylococcus aureus | unaffected pathogenicity |
| gene0260 | Chromosome | potA | PHI:6319 | Streptococcus pneumoniae | reduced virulence |
| gene0266 | Chromosome | pdeB | PHI:8643 | Erwinia amylovora | unaffected pathogenicity |
| gene0267 | Chromosome | iolG | PHI:6382 | Legionella pneumophila | reduced virulence |
| gene0268 | Chromosome | CcpA | PHI:4564 | Enterococcus faecium | reduced virulence |
| gene0274 | Chromosome | potA | PHI:6319 | Streptococcus pneumoniae | reduced virulence |
| gene0277 | Chromosome | VK055_1952 | PHI:7520 | Klebsiella pneumoniae | unaffected pathogenicity |
| gene0289 | Chromosome | CadA | PHI:7386 | Listeria monocytogenes | unaffected pathogenicity |
| gene0295 | Chromosome | MacB | PHI:3928 | Salmonella enterica | reduced virulence |
| gene0297 | Chromosome | rpoS | PHI:3336 | Erwinia amylovora | unaffected pathogenicity |
| gene0303 | Chromosome | potA | PHI:6319 | Streptococcus pneumoniae | reduced virulence |
| gene0304 | Chromosome | potA | PHI:6319 | Streptococcus pneumoniae | reduced virulence |
| gene0308 | Chromosome | potA | PHI:6319 | Streptococcus pneumoniae | reduced virulence |
| gene0311 | Chromosome | gGT | PHI:7689 | Helicobacter pylori | reduced virulence |
| gene0315 | Chromosome | gntK | PHI:6701 | Vibrio cholerae | reduced virulence |
| gene0319 | Chromosome | gbe1 | PHI:4725 | Fusarium oxysporum | unaffected pathogenicity |
| gene0321 | Chromosome | glgC | PHI:6731 | Mycobacterium tuberculosis | reduced virulence |
| gene0323 | Chromosome | Gph1 | PHI:2062 | Magnaporthe oryzae | unaffected pathogenicity |
| gene0324 | Chromosome | glpD | PHI:3272 | Yersinia pestis | unaffected pathogenicity |
| gene0327 | Chromosome | yihW | PHI:6545 | Salmonella enterica | unaffected pathogenicity |
| gene0328 | Chromosome | Gph1 | PHI:2062 | Magnaporthe oryzae | unaffected pathogenicity |
| gene0335 | Chromosome | feoB | PHI:6941 | Pseudomonas aeruginosa | increased virulence (hypervirulence) |
| gene0337 | Chromosome | pnp | PHI:3878 | Salmonella enterica | reduced virulence |
| gene0338 | Chromosome | greB | PHI:7131 | Salmonella enterica | loss of pathogenicity |
| gene0339 | Chromosome | ompR | PHI:2685 | Salmonella enterica | reduced virulence |
| gene0340 | Chromosome | envZ | PHI:2686 | Salmonella enterica | reduced virulence |
| gene0341 | Chromosome | PCK1 | PHI:424 | Cryptococcus neoformans | reduced virulence |
| gene0351 | Chromosome | PilQ | PHI:3827 | Neisseria meningitidis | reduced virulence |
| gene0353 | Chromosome | aroB | PHI:4250 | Burkholderia glumae | reduced virulence |
| gene0354 | Chromosome | damX | PHI:6729 | Escherichia coli | reduced virulence |
| gene0364 | Chromosome | ppiB | PHI:7633 | Pseudomonas aeruginosa | reduced virulence |
| gene0366 | Chromosome | XC_0481 | PHI:3941 | Xanthomonas campestris | reduced virulence |
| gene0367 | Chromosome | argD | PHI:3126 | Erwinia amylovora | unaffected pathogenicity |
| gene0369 | Chromosome | Crp | PHI:4063 | Yersinia pestis | reduced virulence |
| gene0376 | Chromosome | proV | PHI:8968 | Klebsiella pneumoniae | reduced virulence |
| gene0379 | Chromosome | cpxA | PHI:8050 | Actinobacillus pleuropneumoniae | reduced virulence |
| gene0385 | Chromosome | FkpA | PHI:4584 | Cronobacter universalis | reduced virulence |
| gene0390 | Chromosome | GzOB039 | PHI:1599 | Fusarium graminearum | unaffected pathogenicity |
| gene0400 | Chromosome | GzOB009 | PHI:1569 | Fusarium graminearum | lethal |
| gene0417 | Chromosome | secY | PHI:5257 | Listeria monocytogenes | increased virulence (hypervirulence) |
| gene0427 | Chromosome | VK055_1939 | PHI:7533 | Klebsiella pneumoniae | unaffected pathogenicity |
| gene0428 | Chromosome | iroC | PHI:9461 | Salmonella enterica | reduced virulence |
| gene0434 | Chromosome | rsmB | PHI:2694 | Pectobacterium atrosepticum | reduced virulence |
| gene0435 | Chromosome | FMT | PHI:3999 | Staphylococcus aureus | reduced virulence |
| gene0441 | Chromosome | AroE (XOC_4333) | PHI:7791 | Xanthomonas oryzae | reduced virulence |
| gene0448 | Chromosome | cab (NMB0789) | PHI:8623 | Neisseria meningitidis | unaffected pathogenicity |
| gene0452 | Chromosome | lpxL1 (BN49_2155) | PHI:7946 | Klebsiella pneumoniae | unaffected pathogenicity |
| gene0453 | Chromosome | XC_0520 (flp) | PHI:9172 | Xanthomonas campestris | reduced virulence |
| gene0459 | Chromosome | GzC2H048 | PHI:1385 | Fusarium graminearum | unaffected pathogenicity |
| gene0470 | Chromosome | RNase E | PHI:3723 | Salmonella enterica | reduced virulence |
| gene0472 | Chromosome | XC_1510 | PHI:3952 | Xanthomonas campestris | reduced virulence |
| gene0473 | Chromosome | ovrB (Z0346) | PHI:9508 | Escherichia coli | reduced virulence |
| gene0477 | Chromosome | Sdh1 | PHI:3914 | Parastagonospora nodorum | reduced virulence |
| gene0482 | Chromosome | mdh | PHI:2959 | Edwardsiella ictaluri | reduced virulence |
| gene0483 | Chromosome | degQ (Dda3937_00697) | PHI:8575 | Dickeya dadantii | reduced virulence |
| gene0484 | Chromosome | degQ (Dda3937_00697) | PHI:8575 | Dickeya dadantii | reduced virulence |
| gene0491 | Chromosome | cysJ (Dda3937_00209) | PHI:8574 | Dickeya dadantii | reduced virulence |
| gene0494 | Chromosome | PXO_RS14595 | PHI:8994 | Xanthomonas oryzae | unaffected pathogenicity |
| gene0497 | Chromosome | HpaP (XC_1307) | PHI:7824 | Xanthomonas campestris | reduced virulence |
| gene0498 | Chromosome | ptsN | PHI:3133 | Yersinia pseudotuberculosis | reduced virulence |
| gene0499 | Chromosome | yhbH | PHI:3674 | Erwinia amylovora | loss of pathogenicity |
| gene0500 | Chromosome | rpoN | PHI:2906 | Erwinia amylovora | loss of pathogenicity |
| gene0501 | Chromosome | StpC | PHI:6324 | Staphylococcus aureus | unaffected pathogenicity |
| gene0505 | Chromosome | KdsD | PHI:7067 | Francisella tularensis | reduced virulence |
| gene0507 | Chromosome | proV | PHI:8968 | Klebsiella pneumoniae | reduced virulence |
| gene0513 | Chromosome | murA | PHI:7630 | Pseudomonas aeruginosa | unaffected pathogenicity |
| gene0519 | Chromosome | qseC | PHI:3709 | Pectobacterium carotovorum | reduced virulence |
| gene0520 | Chromosome | PmrA | PHI:4497 | Salmonella enterica | increased virulence (hypervirulence) |
| gene0522 | Chromosome | greA | PHI:7130 | Salmonella enterica | loss of pathogenicity |
| gene0525 | Chromosome | GzOB006 | PHI:1566 | Fusarium graminearum | lethal |
| gene0533 | Chromosome | PspK | PHI:6281 | Streptococcus pneumoniae | reduced virulence |
| gene0537 | Chromosome | pnp | PHI:3878 | Salmonella enterica | reduced virulence |
| gene0538 | Chromosome | nlpI | PHI:2459 | Erwinia amylovora | reduced virulence |
| gene0539 | Chromosome | Lmo0866 | PHI:6510 | Listeria monocytogenes | unaffected pathogenicity |
| gene0551 | Chromosome | GzMyb016 | PHI:1552 | Fusarium graminearum | unaffected pathogenicity |
| gene0552 | Chromosome | treS | PHI:6732 | Mycobacterium tuberculosis | unaffected pathogenicity |
| gene0553 | Chromosome | HEX1 | PHI:4987 | Candida albicans | reduced virulence |
| gene0558 | Chromosome | argF | PHI:2634 | Staphylococcus aureus | unaffected pathogenicity |
| gene0595 | Chromosome | fleQ | PHI:3219 | Xanthomonas oryzae | unaffected pathogenicity |
| gene0607 | Chromosome | cadF | PHI:9291 | Campylobacter jejuni | unaffected pathogenicity |
| gene0610 | Chromosome | aroD | PHI:7062 | Salmonella enterica | unaffected pathogenicity |
| gene0614 | Chromosome | cab (NMB0789) | PHI:8623 | Neisseria meningitidis | unaffected pathogenicity |
| gene0622 | Chromosome | cab (NMB0789) | PHI:8623 | Neisseria meningitidis | unaffected pathogenicity |
| gene0625 | Chromosome | fruR | PHI:2672 | Salmonella enterica | reduced virulence |
| gene0628 | Chromosome | pbpX | PHI:4692 | Listeria monocytogenes | reduced virulence |
| gene0629 | Chromosome | cheM | PHI:6983 | Salmonella enterica | reduced virulence |
| gene0634 | Chromosome | ovrB (Z0346) | PHI:9508 | Escherichia coli | reduced virulence |
| gene0636 | Chromosome | rpoS | PHI:3336 | Erwinia amylovora | unaffected pathogenicity |
| gene0648 | Chromosome | rfaE | PHI:3147 | Glaesserella parasuis | reduced virulence |
| gene0649 | Chromosome | yqiC | PHI:6697 | Salmonella enterica | reduced virulence |
| gene0655 | Chromosome | MdsABC | PHI:6553 | Salmonella enterica | reduced virulence |
| gene0662 | Chromosome | SsFdh1 (SS1G_10135) | PHI:9519 | Sclerotinia sclerotiorum | reduced virulence |
| gene0663 | Chromosome | SP_0676 | PHI:3683 | Streptococcus pneumoniae | reduced virulence |
| gene0672 | Chromosome | cab (NMB0789) | PHI:8623 | Neisseria meningitidis | unaffected pathogenicity |
| gene0673 | Chromosome | OxyR | PHI:5305 | Pseudomonas syringae | reduced virulence |
| gene0678 | Chromosome | GyrA | PHI:824 | Burkholderia glumae | loss of pathogenicity |
| gene0679 | Chromosome | mrLPAAT1 | PHI:6380 | Metarhizium robertsii | reduced virulence |
| gene0683 | Chromosome | Bbakr1 (XP_008600433.1) | PHI:9444 | Beauveria bassiana | unaffected pathogenicity |
| gene0686 | Chromosome | PG0343 | PHI:7938 | Porphyromonas gingivalis | reduced virulence |
| gene0695 | Chromosome | XOC_2335 | PHI:6224 | Xanthomonas oryzae | reduced virulence |
| gene0696 | Chromosome | ydcR | PHI:7225 | Salmonella enterica | reduced virulence |
| gene0714 | Chromosome | potA | PHI:6319 | Streptococcus pneumoniae | reduced virulence |
| gene0722 | Chromosome | ccpE | PHI:5242 | Staphylococcus aureus | increased virulence (hypervirulence) |
| gene0727 | Chromosome | AphB | PHI:5548 | Vibrio cholerae | reduced virulence |
| gene0728 | Chromosome | tcp | PHI:6980 | Salmonella enterica | reduced virulence |
| gene0730 | Chromosome | rv3109 | PHI:7746 | Mycobacterium tuberculosis | unaffected pathogenicity |
| gene0741 | Chromosome | MorA | PHI:4684 | Pseudomonas aeruginosa | increased virulence (hypervirulence) |
| gene0743 | Chromosome | NWMN_0826 | PHI:7917 | Staphylococcus aureus | reduced virulence |
| gene0747 | Chromosome | leuB | PHI:9319 | Erwinia amylovora | reduced virulence |
| gene0752 | Chromosome | tssG-2 | PHI:7085 | Erwinia amylovora | unaffected pathogenicity |
| gene0753 | Chromosome | tssJ-2 | PHI:7086 | Erwinia amylovora | unaffected pathogenicity |
| gene0754 | Chromosome | EAMY _3202 | PHI:7082 | Erwinia amylovora | unaffected pathogenicity |
| gene0755 | Chromosome | PafR | PHI:4630 | Escherichia coli | reduced virulence |
| gene0756 | Chromosome | glcA | PHI:6308 | Staphylococcus aureus | reduced virulence |
| gene0757 | Chromosome | BglA3 | PHI:6501 | Streptococcus pneumoniae | reduced virulence |
| gene0759 | Chromosome | katN | PHI:7042 | Escherichia coli | effector (plant avirulence determinant) |
| gene0761 | Chromosome | KatG | PHI:7254 | Erwinia amylovora | reduced virulence |
| gene0764 | Chromosome | cab (NMB0789) | PHI:8623 | Neisseria meningitidis | unaffected pathogenicity |
| gene0765 | Chromosome | ctp (NMB0788) | PHI:8622 | Neisseria meningitidis | unaffected pathogenicity |
| gene0771 | Chromosome | speC | PHI:9151 | Escherichia coli | unaffected pathogenicity |
| gene0785 | Chromosome | pilT | PHI:6363 | Pantoea ananatis | reduced virulence |
| gene0788 | Chromosome | gshB | PHI:9281 | Pseudomonas aeruginosa | reduced virulence |
| gene0792 | Chromosome | CBU_0347 | PHI:9510 | Coxiella burnetii | unaffected pathogenicity |
| gene0793 | Chromosome | MGG_00383 | PHI:877 | Magnaporthe oryzae | reduced virulence |
| gene0795 | Chromosome | speB | PHI:9150 | Escherichia coli | unaffected pathogenicity |
| gene0797 | Chromosome | Tkt | PHI:8028 | Leishmania mexicana | loss of pathogenicity |
| gene0798 | Chromosome | GAPDH | PHI:6904 | Staphylococcus aureus | reduced virulence |
| gene0800 | Chromosome | FbaA | PHI:6903 | Staphylococcus aureus | reduced virulence |
| gene0811 | Chromosome | ubiI | PHI:7028 | Escherichia coli | reduced virulence |
| gene0812 | Chromosome | ubiI | PHI:7028 | Escherichia coli | reduced virulence |
| gene0813 | Chromosome | hopL | PHI:994 | Pseudomonas syringae | effector (plant avirulence determinant) |
| gene0816 | Chromosome | gcvP | PHI:2961 | Edwardsiella ictaluri | reduced virulence |
| gene0817 | Chromosome | fabG1 | PHI:5271 | Ralstonia solanacearum | lethal |
| gene0848 | Chromosome | MntE | PHI:4652 | Streptococcus pyogenes | unaffected pathogenicity |
| gene0849 | Chromosome | Krs (BBA_03037) | PHI:9409 | Beauveria bassiana | reduced virulence |
| gene0850 | Chromosome | expI | PHI:5535 | Pectobacterium carotovorum | reduced virulence |
| gene0851 | Chromosome | ExpR | PHI:4178 | Dickeya solani | unaffected pathogenicity |
| gene0855 | Chromosome | PrhO (Rsc1880) | PHI:7882 | Ralstonia solanacearum | reduced virulence |
| gene0856 | Chromosome | lysA (Dda3937_02577) | PHI:8581 | Dickeya dadantii | reduced virulence |
| gene0858 | Chromosome | chbC | PHI:6529 | Borreliella burgdorferi | unaffected pathogenicity |
| gene0859 | Chromosome | BglA3 | PHI:6501 | Streptococcus pneumoniae | reduced virulence |
| gene0866 | Chromosome | oqxA | PHI:6448 | Klebsiella pneumoniae | reduced virulence |
| gene0867 | Chromosome | yegN | PHI:9198 | Escherichia coli | reduced virulence |
| gene0868 | Chromosome | yegN | PHI:9198 | Escherichia coli | reduced virulence |
| gene0869 | Chromosome | adeK | PHI:6388 | Acinetobacter baumannii | unaffected pathogenicity |
| gene0870 | Chromosome | fadD | PHI:4108 | Vibrio cholerae | unaffected pathogenicity |
| gene0872 | Chromosome | NorA (AflE) | PHI:7768 | Aspergillus flavus | reduced virulence |
| gene0874 | Chromosome | PSPTO_2691 | PHI:3118 | Pseudomonas syringae | unaffected pathogenicity |
| gene0876 | Chromosome | PA0336 (RppH) | PHI:7474 | Pseudomonas aeruginosa | increased virulence (hypervirulence) |
| gene0877 | Chromosome | ptsI | PHI:9312 | Erwinia amylovora | reduced virulence |
| gene0889 | Chromosome | amiC | PHI:9372 | Yersinia pestis | unaffected pathogenicity |
| gene0896 | Chromosome | nifS | PHI:4210 | Mycoplasma agalactiae | loss of pathogenicity |
| gene0898 | Chromosome | ShvR (BCAS0225) | PHI:8616 | Burkholderia cenocepacia | reduced virulence |
| gene0915 | Chromosome | barA | PHI:562 | Salmonella enterica | unaffected pathogenicity |
| gene0917 | Chromosome | RelA | PHI:9161 | Salmonella enterica | reduced virulence |
| gene0926 | Chromosome | cysJ (Dda3937_00209) | PHI:8574 | Dickeya dadantii | reduced virulence |
| gene0927 | Chromosome | cysI3 | PHI:9323 | Erwinia amylovora | unaffected pathogenicity |
| gene0931 | Chromosome | met8 (AFUA_7G05680) | PHI:8590 | Aspergillus fumigatus | unaffected pathogenicity |
| gene0932 | Chromosome | raxP (cysD) | PHI:1138 | Xanthomonas oryzae | increased virulence (hypervirulence) |
| gene0933 | Chromosome | raxQ | PHI:1139 | Xanthomonas oryzae | increased virulence (hypervirulence) |
| gene0934 | Chromosome | raxQ | PHI:1139 | Xanthomonas oryzae | increased virulence (hypervirulence) |
| gene0938 | Chromosome | XAC1722 | PHI:4145 | Xanthomonas citri | reduced virulence |
| gene0941 | Chromosome | pimt | PHI:6187 | Salmonella enterica | reduced virulence |
| gene0942 | Chromosome | nlpD | PHI:9369 | Yersinia pestis | reduced virulence |
| gene0943 | Chromosome | rpoS | PHI:3336 | Erwinia amylovora | unaffected pathogenicity |
| gene0947 | Chromosome | iaaH-1 | PHI:4171 | Pseudomonas savastanoi | reduced virulence |
| gene0948 | Chromosome | msh2 | PHI:7207 | Cryptococcus neoformans | unaffected pathogenicity |
| gene0960 | Chromosome | CBU_0347 | PHI:9510 | Coxiella burnetii | unaffected pathogenicity |
| gene0972 | Chromosome | pecS | PHI:4176 | Dickeya solani | increased virulence (hypervirulence) |
| gene0973 | Chromosome | cifA | PHI:2934 | Pseudomonas cichorii | reduced virulence |
| gene0974 | Chromosome | CBU_0347 | PHI:9510 | Coxiella burnetii | unaffected pathogenicity |
| gene0976 | Chromosome | VK055_1980 | PHI:7492 | Klebsiella pneumoniae | unaffected pathogenicity |
| gene0978 | Chromosome | degU | PHI:4690 | Listeria monocytogenes | reduced virulence |
| gene0985 | Chromosome | EntA (VK055_1922) | PHI:7485 | Klebsiella pneumoniae | unaffected pathogenicity |
| gene0986 | Chromosome | BB_RS07570 (BB1518) | PHI:9188 | Bordetella bronchiseptica | unaffected pathogenicity |
| gene0989 | Chromosome | fabG1 | PHI:5271 | Ralstonia solanacearum | lethal |
| gene0991 | Chromosome | CBU_0265 | PHI:9509 | Coxiella burnetii | unaffected pathogenicity |
| gene0995 | Chromosome | recA | PHI:4702 | Streptococcus pneumoniae | reduced virulence |
| gene0998 | Chromosome | csrA | PHI:7161 | Erwinia amylovora | reduced virulence |
| gene1006 | Chromosome | gshA | PHI:9280 | Pseudomonas aeruginosa | reduced virulence |
| gene1007 | Chromosome | LuxS | PHI:3731 | Escherichia coli | reduced virulence |
| gene1017 | Chromosome | aroG2 | PHI:8885 | Ralstonia solanacearum | unaffected pathogenicity |
| gene1022 | Chromosome | yfiA | PHI:3675 | Erwinia amylovora | unaffected pathogenicity |
| gene1026 | Chromosome | clpV-2 | PHI:7094 | Erwinia amylovora | unaffected pathogenicity |
| gene1034 | Chromosome | Pat | PHI:5571 | Salmonella enterica | reduced virulence |
| gene1040 | Chromosome | MprA | PHI:5407 | Escherichia coli | reduced virulence |
| gene1045 | Chromosome | proV | PHI:8968 | Klebsiella pneumoniae | reduced virulence |
| gene1061 | Chromosome | MorA | PHI:4684 | Pseudomonas aeruginosa | increased virulence (hypervirulence) |
| gene1067 | Chromosome | smpB | PHI:4218 | Escherichia coli | reduced virulence |
| gene1076 | Chromosome | Lmo0866 | PHI:6510 | Listeria monocytogenes | unaffected pathogenicity |
| gene1078 | Chromosome | nadB | PHI:8507 | Coxiella burnetii | reduced virulence |
| gene1079 | Chromosome | rpoE | PHI:2680 | Salmonella enterica | reduced virulence |
| gene1085 | Chromosome | RNase III | PHI:3724 | Salmonella enterica | reduced virulence |
| gene1086 | Chromosome | mnmE | PHI:9270 | Streptococcus suis | reduced virulence |
| gene1095 | Chromosome | purL | PHI:9320 | Erwinia amylovora | reduced virulence |
| gene1097 | Chromosome | cpxA | PHI:8513 | Citrobacter rodentium | unaffected pathogenicity |
| gene1098 | Chromosome | qseG | PHI:9123 | Citrobacter rodentium | reduced virulence |
| gene1099 | Chromosome | PXO_RS02690 | PHI:9014 | Xanthomonas oryzae | unaffected pathogenicity |
| gene1101 | Chromosome | fhpB | PHI:3276 | Aspergillus fumigatus | unaffected pathogenicity |
| gene1102 | Chromosome | glyA | PHI:2962 | Edwardsiella ictaluri | reduced virulence |
| gene1107 | Chromosome | SuhB | PHI:3915 | Pseudomonas aeruginosa | loss of pathogenicity |
| gene1109 | Chromosome | iscR | PHI:3043 | Yersinia pseudotuberculosis | reduced virulence |
| gene1112 | Chromosome | sseB | PHI:3761 | Salmonella enterica | effector (plant avirulence determinant) |
| gene1113 | Chromosome | sseA | PHI:609 | Salmonella enterica | reduced virulence |
| gene1114 | Chromosome | XC_2203 | PHI:3945 | Xanthomonas campestris | reduced virulence |
| gene1122 | Chromosome | mnmE | PHI:9270 | Streptococcus suis | reduced virulence |
| gene1124 | Chromosome | XC_2659 | PHI:9170 | Xanthomonas campestris | unaffected pathogenicity |
| gene1130 | Chromosome | pagC | PHI:3777 | Salmonella enterica | effector (plant avirulence determinant) |
| gene1134 | Chromosome | guaB | PHI:9306 | Erwinia amylovora | reduced virulence |
| gene1135 | Chromosome | gua1 | PHI:6871 | Cryptococcus neoformans | loss of pathogenicity |
| gene1138 | Chromosome | rpfR (bcam0580) | PHI:8839 | Burkholderia cenocepacia | increased virulence (hypervirulence) |
| gene1141 | Chromosome | ppk | PHI:5063 | Proteus mirabilis | reduced virulence |
| gene1143 | Chromosome | Rv0930 | PHI:3635 | Mycobacterium tuberculosis | unaffected pathogenicity |
| gene1144 | Chromosome | PstB | PHI:9450 | Staphylococcus aureus | unaffected pathogenicity |
| gene1145 | Chromosome | purN | PHI:8652 | Escherichia coli | unaffected pathogenicity |
| gene1146 | Chromosome | ADE5 | PHI:744 | Fusarium graminearum | reduced virulence |
| gene1151 | Chromosome | Oma1 | PHI:7048 | Candida albicans | increased virulence (hypervirulence) |
| gene1158 | Chromosome | potA | PHI:6319 | Streptococcus pneumoniae | reduced virulence |
| gene1160 | Chromosome | ECA3851 | PHI:7691 | Pectobacterium atrosepticum | reduced virulence |
| gene1174 | Chromosome | AcrD | PHI:4043 | Erwinia amylovora | unaffected pathogenicity |
| gene1181 | Chromosome | Tkt | PHI:8028 | Leishmania mexicana | loss of pathogenicity |
| gene1184 | Chromosome | amiA | PHI:8728 | Salmonella enterica | unaffected pathogenicity |
| gene1191 | Chromosome | potC | PHI:6321 | Streptococcus pneumoniae | reduced virulence |
| gene1193 | Chromosome | potA | PHI:6319 | Streptococcus pneumoniae | reduced virulence |
| gene1194 | Chromosome | mecA | PHI:6556 | Aspergillus fumigatus | unaffected pathogenicity |
| gene1195 | Chromosome | cpxR | PHI:8512 | Citrobacter rodentium | unaffected pathogenicity |
| gene1196 | Chromosome | cpxA | PHI:8513 | Citrobacter rodentium | unaffected pathogenicity |
| gene1198 | Chromosome | glcA | PHI:6308 | Staphylococcus aureus | reduced virulence |
| gene1199 | Chromosome | ptsI | PHI:9312 | Erwinia amylovora | reduced virulence |
| gene1200 | Chromosome | ptsH | PHI:9311 | Erwinia amylovora | reduced virulence |
| gene1201 | Chromosome | mecA | PHI:6556 | Aspergillus fumigatus | unaffected pathogenicity |
| gene1204 | Chromosome | adeN | PHI:7809 | Acinetobacter baumannii | increased virulence (hypervirulence) |
| gene1207 | Chromosome | OxyR | PHI:3714 | Escherichia coli | reduced virulence |
| gene1224 | Chromosome | mntH2 | PHI:8587 | Enterococcus faecalis | unaffected pathogenicity |
| gene1230 | Chromosome | 1910HK (ssu05 1911) | PHI:6896 | Streptococcus suis | reduced virulence |
| gene1233 | Chromosome | oqxA | PHI:6448 | Klebsiella pneumoniae | reduced virulence |
| gene1252 | Chromosome | tssA-3 | PHI:7079 | Erwinia amylovora | unaffected pathogenicity |
| gene1260 | Chromosome | CcmA (VK055_1938) | PHI:7534 | Klebsiella pneumoniae | unaffected pathogenicity |
| gene1261 | Chromosome | CcmA (VK055_1938) | PHI:7534 | Klebsiella pneumoniae | unaffected pathogenicity |
| gene1269 | Chromosome | vacJ | PHI:6900 | Glaesserella parasuis | reduced virulence |
| gene1272 | Chromosome | ERG10 | PHI:2529 | Aspergillus fumigatus | lethal |
| gene1273 | Chromosome | FgEch1 (FGSG_13111) | PHI:9517 | Fusarium graminearum | reduced virulence |
| gene1276 | Chromosome | XC_1565 | PHI:3966 | Xanthomonas campestris | reduced virulence |
| gene1277 | Chromosome | aroB | PHI:7654 | Aspergillus fumigatus | reduced virulence |
| gene1283 | Chromosome | KSA1 | PHI:724 | Fusarium graminearum | unaffected pathogenicity |
| gene1287 | Chromosome | VK055_1939 | PHI:7533 | Klebsiella pneumoniae | unaffected pathogenicity |
| gene1288 | Chromosome | proV | PHI:8968 | Klebsiella pneumoniae | reduced virulence |
| gene1297 | Chromosome | PXO_RS19415 | PHI:8999 | Xanthomonas oryzae | unaffected pathogenicity |
| gene1298 | Chromosome | PidS | PHI:2439 | Burkholderia glumae | reduced virulence |
| gene1308 | Chromosome | purF | PHI:9317 | Erwinia amylovora | reduced virulence |
| gene1310 | Chromosome | bab2_0612 | PHI:7201 | Brucella abortus | reduced virulence |
| gene1313 | Chromosome | cab (NMB0789) | PHI:8623 | Neisseria meningitidis | unaffected pathogenicity |
| gene1317 | Chromosome | Bsi | PHI:7698 | Pseudomonas syringae | increased virulence (hypervirulence) |
| gene1318 | Chromosome | eutD | PHI:6516 | Mycoplasma agalactiae | reduced virulence |
| gene1326 | Chromosome | LrhA | PHI:7647 | Escherichia coli | reduced virulence |
| gene1329 | Chromosome | NOS1 | PHI:445 | Fusarium graminearum | reduced virulence |
| gene1334 | Chromosome | nuoI (PA2644) | PHI:8937 | Pseudomonas aeruginosa | unaffected pathogenicity |
| gene1335 | Chromosome | nuoJ (PA2645) | PHI:8938 | Pseudomonas aeruginosa | unaffected pathogenicity |
| gene1337 | Chromosome | MnhA2 | PHI:7819 | Staphylococcus aureus | unaffected pathogenicity |
| gene1341 | Chromosome | opgG | PHI:5564 | Dickeya dadantii | reduced virulence |
| gene1350 | Chromosome | pvdL | PHI:6991 | Pseudomonas aeruginosa | loss of pathogenicity |
| gene1356 | Chromosome | gyrA | PHI:824 | Burkholderia glumae | loss of pathogenicity |
| gene1357 | Chromosome | rcsC | PHI:2500 | Erwinia amylovora | loss of pathogenicity |
| gene1358 | Chromosome | rcsB | PHI:2499 | Erwinia amylovora | loss of pathogenicity |
| gene1359 | Chromosome | rcsD | PHI:4481 | Erwinia amylovora | loss of pathogenicity |
| gene1362 | Chromosome | tolC | PHI:3710 | Pectobacterium carotovorum | reduced virulence |
| gene1363 | Chromosome | LssB | PHI:4975 | Legionella pneumophila | reduced virulence |
| gene1364 | Chromosome | LssD | PHI:4976 | Legionella pneumophila | reduced virulence |
| gene1365 | Chromosome | OmpK36 | PHI:4161 | Klebsiella pneumoniae | reduced virulence |
| gene1369 | Chromosome | potA | PHI:6319 | Streptococcus pneumoniae | reduced virulence |
| gene1377 | Chromosome | RplY | PHI:7469 | Pectobacterium carotovorum | reduced virulence |
| gene1385 | Chromosome | oppD (bb0334) | PHI:7820 | Borreliella burgdorferi | reduced virulence |
| gene1387 | Chromosome | App | PHI:4612 | Clostridioides difficile | increased virulence (hypervirulence) |
| gene1396 | Chromosome | fruK | PHI:6527 | Borreliella burgdorferi | unaffected pathogenicity |
| gene1397 | Chromosome | fruA1 | PHI:6524 | Borreliella burgdorferi | unaffected pathogenicity |
| gene1398 | Chromosome | ANP1 | PHI:3793 | Candida glabrata | increased virulence (hypervirulence) |
| gene1403 | Chromosome | hmuU | PHI:7908 | Yersinia pseudotuberculosis | unaffected pathogenicity |
| gene1404 | Chromosome | FepC (VK055_1930) | PHI:7541 | Klebsiella pneumoniae | reduced virulence |
| gene1405 | Chromosome | ireA | PHI:6654 | Escherichia coli | unaffected pathogenicity |
| gene1407 | Chromosome | gnoA | PHI:3277 | Aspergillus fumigatus | unaffected pathogenicity |
| gene1408 | Chromosome | hdfR (Dda3937_04419) | PHI:8567 | Dickeya dadantii | reduced virulence |
| gene1412 | Chromosome | CcpA | PHI:4564 | Enterococcus faecium | reduced virulence |
| gene1413 | Chromosome | bab2_1109 | PHI:9129 | Brucella abortus | reduced virulence |
| gene1414 | Chromosome | oppF (bb0335) | PHI:7821 | Borreliella burgdorferi | reduced virulence |
| gene1429 | Chromosome | EAMY _3223 | PHI:7108 | Erwinia amylovora | unaffected pathogenicity |
| gene1436 | Chromosome | Xdh1 | PHI:2256 | Parastagonospora nodorum | unaffected pathogenicity |
| gene1439 | Chromosome | hp0169 | PHI:6964 | Helicobacter pylori | reduced virulence |
| gene1440 | Chromosome | SrrAB | PHI:5466 | Staphylococcus aureus | reduced virulence |
| gene1441 | Chromosome | cpxA | PHI:8513 | Citrobacter rodentium | unaffected pathogenicity |
| gene1442 | Chromosome | BCMFS1 | PHI:544 | Botrytis cinerea | unaffected pathogenicity |
| gene1443 | Chromosome | yegN | PHI:9198 | Escherichia coli | reduced virulence |
| gene1444 | Chromosome | yegN | PHI:9198 | Escherichia coli | reduced virulence |
| gene1446 | Chromosome | XC_0281 | PHI:3947 | Xanthomonas campestris | reduced virulence |
| gene1448 | Chromosome | MorA | PHI:4684 | Pseudomonas aeruginosa | increased virulence (hypervirulence) |
| gene1451 | Chromosome | magA | PHI:2652 | Klebsiella pneumoniae | reduced virulence |
| gene1453 | Chromosome | PSPTO_2691 | PHI:3118 | Pseudomonas syringae | unaffected pathogenicity |
| gene1454 | Chromosome | cps2E | PHI:6138 | Streptococcus suis | reduced virulence |
| gene1457 | Chromosome | Ams | PHI:2471 | Erwinia amylovora | loss of pathogenicity |
| gene1466 | Chromosome | galU | PHI:3112 | Xanthomonas campestris | loss of pathogenicity |
| gene1467 | Chromosome | galE | PHI:9264 | Haemophilus influenzae | unaffected pathogenicity |
| gene1471 | Chromosome | nab2 (VV1_0808) | PHI:8517 | Vibrio vulnificus | reduced virulence |
| gene1472 | Chromosome | gigX6 | PHI:6958 | Xanthomonas oryzae | increased virulence (hypervirulence) |
| gene1474 | Chromosome | nab1 (VV1_0803) | PHI:8516 | Vibrio vulnificus | reduced virulence |
| gene1481 | Chromosome | WzzST | PHI:3728 | Salmonella enterica | loss of pathogenicity |
| gene1485 | Chromosome | hisA | PHI:9324 | Erwinia amylovora | unaffected pathogenicity |
| gene1487 | Chromosome | HisB | PHI:7790 | Xanthomonas oryzae | reduced virulence |
| gene1490 | Chromosome | prt | PHI:5109 | Salmonella enterica | reduced virulence |
| gene1496 | Chromosome | ArgD | PHI:8863 | Salmonella enterica | reduced virulence |
| gene1508 | Chromosome | CcpA | PHI:4564 | Enterococcus faecium | reduced virulence |
| gene1527 | Chromosome | bab2_0822 | PHI:9128 | Brucella abortus | reduced virulence |
| gene1530 | Chromosome | StpC | PHI:6324 | Staphylococcus aureus | unaffected pathogenicity |
| gene1531 | Chromosome | PstB | PHI:9450 | Staphylococcus aureus | unaffected pathogenicity |
| gene1544 | Chromosome | cel-EIIB (SAG0329) | PHI:8620 | Streptococcus agalactiae | reduced virulence |
| gene1545 | Chromosome | chbC | PHI:6529 | Borreliella burgdorferi | unaffected pathogenicity |
| gene1546 | Chromosome | BglA3 | PHI:6501 | Streptococcus pneumoniae | reduced virulence |
| gene1550 | Chromosome | iaaH-1 | PHI:4171 | Pseudomonas savastanoi | reduced virulence |
| gene1551 | Chromosome | XC_2801 | PHI:3260 | Xanthomonas campestris | reduced virulence |
| gene1552 | Chromosome | ADH1 | PHI:9524 | Candida albicans | reduced virulence |
| gene1555 | Chromosome | pobA | PHI:5404 | Xanthomonas campestris | reduced virulence |
| gene1557 | Chromosome | ovrB (Z0346) | PHI:9508 | Escherichia coli | reduced virulence |
| gene1564 | Chromosome | F-avi4330 | PHI:2895 | Agrobacterium vitis | loss of pathogenicity |
| gene1568 | Chromosome | aphB | PHI:5548 | Vibrio cholerae | reduced virulence |
| gene1572 | Chromosome | metR | PHI:9326 | Erwinia amylovora | unaffected pathogenicity |
| gene1576 | Chromosome | Smp | PHI:7699 | Pseudomonas syringae | unaffected pathogenicity |
| gene1583 | Chromosome | vexD | PHI:7772 | Vibrio cholerae | reduced virulence |
| gene1584 | Chromosome | KSA1 | PHI:724 | Fusarium graminearum | unaffected pathogenicity |
| gene1590 | Chromosome | proV | PHI:8968 | Klebsiella pneumoniae | reduced virulence |
| gene1591 | Chromosome | VK055_1939 | PHI:7533 | Klebsiella pneumoniae | unaffected pathogenicity |
| gene1593 | Chromosome | SoxR | PHI:3092 | Pantoea stewartii | reduced virulence |
| gene1594 | Chromosome | cysB | PHI:8764 | Pseudomonas aeruginosa | reduced virulence |
| gene1595 | Chromosome | Sdh1 | PHI:3914 | Parastagonospora nodorum | reduced virulence |
| gene1596 | Chromosome | AldA (PSPTO_0092) | PHI:7787 | Pseudomonas syringae | reduced virulence |
| gene1602 | Chromosome | oppF (bb0335) | PHI:7821 | Borreliella burgdorferi | reduced virulence |
| gene1606 | Chromosome | argD | PHI:3126 | Erwinia amylovora | unaffected pathogenicity |
| gene1607 | Chromosome | lrp | PHI:6497 | Xenorhabdus nematophila | reduced virulence |
| gene1609 | Chromosome | FgOXP1 | PHI:7717 | Fusarium graminearum | reduced virulence |
| gene1611 | Chromosome | spxB | PHI:3604 | Streptococcus pneumoniae | increased virulence (hypervirulence) |
| gene1612 | Chromosome | VK055_1961 | PHI:7511 | Klebsiella pneumoniae | unaffected pathogenicity |
| gene1618 | Chromosome | oppF (bb0335) | PHI:7821 | Borreliella burgdorferi | reduced virulence |
| gene1619 | Chromosome | FepC (VK055_1930) | PHI:7541 | Klebsiella pneumoniae | reduced virulence |
| gene1623 | Chromosome | VraD | PHI:6579 | Staphylococcus aureus | reduced virulence |
| gene1632 | Chromosome | fabD | PHI:7916 | Staphylococcus aureus | reduced virulence |
| gene1633 | Chromosome | fabG1 | PHI:5271 | Ralstonia solanacearum | lethal |
| gene1634 | Chromosome | Acp1 (PA1869) | PHI:8556 | Pseudomonas aeruginosa | increased virulence (hypervirulence) |
| gene1635 | Chromosome | KSA1 | PHI:724 | Fusarium graminearum | unaffected pathogenicity |
| gene1641 | Chromosome | glcB | PHI:6309 | Staphylococcus aureus | reduced virulence |
| gene1646 | Chromosome | NagZ | PHI:3282 | Xanthomonas campestris | reduced virulence |
| gene1648 | Chromosome | ndh (PA4538) | PHI:8939 | Pseudomonas aeruginosa | unaffected pathogenicity |
| gene1652 | Chromosome | Mfd | PHI:6483 | Bacillus cereus | loss of pathogenicity |
| gene1655 | Chromosome | MacB | PHI:3928 | Salmonella enterica | reduced virulence |
| gene1657 | Chromosome | GLKA | PHI:2631 | Mycobacterium tuberculosis | unaffected pathogenicity |
| gene1658 | Chromosome | cob | PHI:5572 | Salmonella enterica | unaffected pathogenicity |
| gene1663 | Chromosome | PhoQ | PHI:4899 | Salmonella enterica | unaffected pathogenicity |
| gene1664 | Chromosome | phoP | PHI:9465 | Salmonella enterica | reduced virulence |
| gene1688 | Chromosome | fiuA | PHI:6373 | Pseudomonas aeruginosa | reduced virulence |
| gene1689 | Chromosome | treA | PHI:6630 | Xanthomonas citri | increased virulence (hypervirulence) |
| gene1691 | Chromosome | pafP | PHI:4631 | Escherichia coli | reduced virulence |
| gene1695 | Chromosome | NWMN_0826 | PHI:7917 | Staphylococcus aureus | reduced virulence |
| gene1697 | Chromosome | PmrA | PHI:4497 | Salmonella enterica | increased virulence (hypervirulence) |
| gene1698 | Chromosome | rcsC | PHI:4480 | Erwinia amylovora | loss of pathogenicity |
| gene1701 | Chromosome | Rv1290c | PHI:3643 | Mycobacterium tuberculosis | reduced virulence |
| gene1702 | Chromosome | trg | PHI:6982 | Salmonella enterica | reduced virulence |
| gene1703 | Chromosome | GLX3 | PHI:8693 | Candida albicans | loss of pathogenicity |
| gene1721 | Chromosome | leuB | PHI:9319 | Erwinia amylovora | reduced virulence |
| gene1727 | Chromosome | LoiA (STM14_1008) | PHI:7217 | Salmonella enterica | unaffected pathogenicity |
| gene1729 | Chromosome | fabG1 | PHI:5271 | Ralstonia solanacearum | lethal |
| gene1733 | Chromosome | fabG1 | PHI:5271 | Ralstonia solanacearum | lethal |
| gene1735 | Chromosome | ovrB (Z0346) | PHI:9508 | Escherichia coli | reduced virulence |
| gene1746 | Chromosome | bcam2836 | PHI:8835 | Burkholderia cenocepacia | increased virulence (hypervirulence) |
| gene1747 | Chromosome | FTT0673p/prsAp | PHI:4732 | Francisella tularensis | unaffected pathogenicity |
| gene1751 | Chromosome | MntE | PHI:4652 | Streptococcus pyogenes | unaffected pathogenicity |
| gene1760 | Chromosome | EAMY _3004 | PHI:7392 | Erwinia amylovora | unaffected pathogenicity |
| gene1762 | Chromosome | EAMY _3011 | PHI:7399 | Erwinia amylovora | unaffected pathogenicity |
| gene1763 | Chromosome | tagH-2 | PHI:7096 | Erwinia amylovora | reduced virulence |
| gene1764 | Chromosome | EAMY _3023 | PHI:7411 | Erwinia amylovora | unaffected pathogenicity |
| gene1765 | Chromosome | tssM-2 | PHI:7105 | Erwinia amylovora | unaffected pathogenicity |
| gene1766 | Chromosome | tssL-2 | PHI:7104 | Erwinia amylovora | unaffected pathogenicity |
| gene1767 | Chromosome | EAMY _3028 | PHI:7416 | Erwinia amylovora | unaffected pathogenicity |
| gene1771 | Chromosome | proV | PHI:8968 | Klebsiella pneumoniae | reduced virulence |
| gene1778 | Chromosome | entA | PHI:4077 | Bacillus cereus | reduced virulence |
| gene1781 | Chromosome | proV | PHI:8968 | Klebsiella pneumoniae | reduced virulence |
| gene1782 | Chromosome | VK055_1939 | PHI:7533 | Klebsiella pneumoniae | unaffected pathogenicity |
| gene1784 | Chromosome | entA | PHI:4077 | Bacillus cereus | reduced virulence |
| gene1786 | Chromosome | ychO | PHI:6184 | Escherichia coli | reduced virulence |
| gene1787 | Chromosome | argD | PHI:3126 | Erwinia amylovora | unaffected pathogenicity |
| gene1789 | Chromosome | AldA (PSPTO_0092) | PHI:7787 | Pseudomonas syringae | reduced virulence |
| gene1800 | Chromosome | katE | PHI:6090 | Acinetobacter nosocomialis | increased virulence (hypervirulence) |
| gene1815 | Chromosome | hupB (XAC1081) | PHI:8676 | Xanthomonas citri | reduced virulence |
| gene1816 | Chromosome | hmuU | PHI:7908 | Yersinia pseudotuberculosis | unaffected pathogenicity |
| gene1817 | Chromosome | MoHYR1 | PHI:2153 | Magnaporthe oryzae | reduced virulence |
| gene1823 | Chromosome | XC_2801 | PHI:3260 | Xanthomonas campestris | reduced virulence |
| gene1825 | Chromosome | aroG2 | PHI:8885 | Ralstonia solanacearum | unaffected pathogenicity |
| gene1830 | Chromosome | ybdB | PHI:6270 | Escherichia coli | lethal |
| gene1832 | Chromosome | sufB | PHI:6893 | Salmonella enterica | unaffected pathogenicity |
| gene1833 | Chromosome | sufC | PHI:6894 | Salmonella enterica | unaffected pathogenicity |
| gene1839 | Chromosome | pykF | PHI:3134 | Yersinia pseudotuberculosis | reduced virulence |
| gene1845 | Chromosome | CFAS | PHI:2643 | Leishmania infantum | reduced virulence |
| gene1846 | Chromosome | CaNAG4 | PHI:511 | Candida albicans | reduced virulence |
| gene1847 | Chromosome | SoxR | PHI:3092 | Pantoea stewartii | reduced virulence |
| gene1851 | Chromosome | Grx4 (CNAG_02950) | PHI:8615 | Cryptococcus neoformans | loss of pathogenicity |
| gene1853 | Chromosome | GOX1 | PHI:414 | Parastagonospora nodorum | unaffected pathogenicity |
| gene1855 | Chromosome | LptA | PHI:4910 | Haemophilus ducreyi | unaffected pathogenicity |
| gene1859 | Chromosome | oqxA | PHI:6448 | Klebsiella pneumoniae | reduced virulence |
| gene1861 | Chromosome | slyA | PHI:2678 | Salmonella enterica | reduced virulence |
| gene1864 | Chromosome | mliC | PHI:4981 | Edwardsiella tarda | reduced virulence |
| gene1865 | Chromosome | PhzG1 | PHI:7298 | Pseudomonas aeruginosa | reduced virulence |
| gene1869 | Chromosome | aphB | PHI:5548 | Vibrio cholerae | reduced virulence |
| gene1870 | Chromosome | Smp | PHI:7699 | Pseudomonas syringae | unaffected pathogenicity |
| gene1873 | Chromosome | BetI (VK055_1951) | PHI:7521 | Klebsiella pneumoniae | unaffected pathogenicity |
| gene1874 | Chromosome | VK055_1952 | PHI:7520 | Klebsiella pneumoniae | unaffected pathogenicity |
| gene1875 | Chromosome | bab2_0277 | PHI:9126 | Brucella abortus | reduced virulence |
| gene1882 | Chromosome | nqrE (PA2995) | PHI:8941 | Pseudomonas aeruginosa | unaffected pathogenicity |
| gene1885 | Chromosome | expI | PHI:4179 | Dickeya solani | reduced virulence |
| gene1886 | Chromosome | bab2_1109 | PHI:9129 | Brucella abortus | reduced virulence |
| gene1887 | Chromosome | potA | PHI:6319 | Streptococcus pneumoniae | reduced virulence |
| gene1889 | Chromosome | VK055_1939 | PHI:7533 | Klebsiella pneumoniae | unaffected pathogenicity |
| gene1895 | Chromosome | potA | PHI:6319 | Streptococcus pneumoniae | reduced virulence |
| gene1901 | Chromosome | pafP | PHI:4631 | Escherichia coli | reduced virulence |
| gene1904 | Chromosome | MAN1 | PHI:220 | Cryptococcus neoformans | reduced virulence |
| gene1907 | Chromosome | hns | PHI:6369 | Escherichia coli | increased virulence (hypervirulence) |
| gene1914 | Chromosome | msrA2 | PHI:4580 | Staphylococcus aureus | unaffected pathogenicity |
| gene1916 | Chromosome | OxyR | PHI:5305 | Pseudomonas syringae | reduced virulence |
| gene1919 | Chromosome | proV | PHI:8968 | Klebsiella pneumoniae | reduced virulence |
| gene1927 | Chromosome | VK055_1961 | PHI:7511 | Klebsiella pneumoniae | unaffected pathogenicity |
| gene1932 | Chromosome | cheM | PHI:6983 | Salmonella enterica | reduced virulence |
| gene1933 | Chromosome | bcam2822 | PHI:8834 | Burkholderia cenocepacia | increased virulence (hypervirulence) |
| gene1943 | Chromosome | FgShyC (FGSG_03657) | PHI:8803 | Fusarium graminearum | unaffected pathogenicity |
| gene1952 | Chromosome | metR | PHI:9326 | Erwinia amylovora | unaffected pathogenicity |
| gene1953 | Chromosome | AldB (PSPTO_2673) | PHI:7788 | Pseudomonas syringae | reduced virulence |
| gene1954 | Chromosome | ECA2008 | PHI:7690 | Pectobacterium atrosepticum | increased virulence (hypervirulence) |
| gene1958 | Chromosome | PA2414 | PHI:4717 | Pseudomonas aeruginosa | reduced virulence |
| gene1959 | Chromosome | XC_0570 | PHI:3961 | Xanthomonas campestris | reduced virulence |
| gene1961 | Chromosome | cps2J | PHI:4130 | Streptococcus suis | reduced virulence |
| gene1963 | Chromosome | XC_2659 | PHI:9170 | Xanthomonas campestris | unaffected pathogenicity |
| gene1965 | Chromosome | cspE | PHI:7263 | Salmonella enterica | unaffected pathogenicity |
| gene1974 | Chromosome | CcpA | PHI:4564 | Enterococcus faecium | reduced virulence |
| gene1976 | Chromosome | FVEG_12531 | PHI:3383 | Fusarium verticillioides | unaffected pathogenicity |
| gene1981 | Chromosome | Xdh1 | PHI:2256 | Parastagonospora nodorum | unaffected pathogenicity |
| gene1989 | Chromosome | PXO_RS02690 | PHI:9014 | Xanthomonas oryzae | unaffected pathogenicity |
| gene1993 | Chromosome | XC_2801 | PHI:3260 | Xanthomonas campestris | reduced virulence |
| gene1995 | Chromosome | THR | PHI:2312 | Alternaria alternata | unaffected pathogenicity |
| gene1998 | Chromosome | XC_2659 | PHI:9170 | Xanthomonas campestris | unaffected pathogenicity |
| gene2006 | Chromosome | pvdH | PHI:5059 | Pseudomonas aeruginosa | unaffected pathogenicity |
| gene2008 | Chromosome | fabG1 | PHI:5271 | Ralstonia solanacearum | lethal |
| gene2017 | Chromosome | potA | PHI:6319 | Streptococcus pneumoniae | reduced virulence |
| gene2018 | Chromosome | VK055_1939 | PHI:7533 | Klebsiella pneumoniae | unaffected pathogenicity |
| gene2019 | Chromosome | Tkt | PHI:8028 | Leishmania mexicana | loss of pathogenicity |
| gene2025 | Chromosome | BdlA | PHI:3041 | Pseudomonas aeruginosa | reduced virulence |
| gene2034 | Chromosome | bab2_1109 | PHI:9129 | Brucella abortus | reduced virulence |
| gene2035 | Chromosome | VK055_1939 | PHI:7533 | Klebsiella pneumoniae | unaffected pathogenicity |
| gene2036 | Chromosome | proV | PHI:8968 | Klebsiella pneumoniae | reduced virulence |
| gene2038 | Chromosome | Xdh1 | PHI:2256 | Parastagonospora nodorum | unaffected pathogenicity |
| gene2046 | Chromosome | ireA | PHI:6654 | Escherichia coli | unaffected pathogenicity |
| gene2052 | Chromosome | DipA | PHI:3042 | Pseudomonas aeruginosa | reduced virulence |
| gene2053 | Chromosome | zmpR | PHI:3008 | Streptococcus pneumoniae | unaffected pathogenicity |
| gene2057 | Chromosome | MgMfs1 | PHI:1161 | Zymoseptoria tritici | unaffected pathogenicity |
| gene2064 | Chromosome | MCC | PHI:5068 | Fusarium graminearum | reduced virulence |
| gene2073 | Chromosome | adhT | PHI:6517 | Mycoplasma agalactiae | reduced virulence |
| gene2074 | Chromosome | BdlA | PHI:3041 | Pseudomonas aeruginosa | reduced virulence |
| gene2075 | Chromosome | potA | PHI:6319 | Streptococcus pneumoniae | reduced virulence |
| gene2081 | Chromosome | bab2_1109 | PHI:9129 | Brucella abortus | reduced virulence |
| gene2083 | Chromosome | tsr | PHI:6981 | Salmonella enterica | reduced virulence |
| gene2085 | Chromosome | MarA | PHI:5369 | Salmonella enterica | reduced virulence |
| gene2086 | Chromosome | oqxA | PHI:6448 | Klebsiella pneumoniae | reduced virulence |
| gene2087 | Chromosome | oqxB | PHI:6449 | Klebsiella pneumoniae | reduced virulence |
| gene2098 | Chromosome | QsvR (VPA0606) | PHI:8742 | Vibrio parahaemolyticus | reduced virulence |
| gene2101 | Chromosome | AldB (PSPTO_2673) | PHI:7788 | Pseudomonas syringae | reduced virulence |
| gene2105 | Chromosome | vtlR | PHI:5012 | Brucella abortus | reduced virulence |
| gene2113 | Chromosome | XC_1368 | PHI:3951 | Xanthomonas campestris | reduced virulence |
| gene2117 | Chromosome | vtlR | PHI:5012 | Brucella abortus | reduced virulence |
| gene2118 | Chromosome | mcpB | PHI:6985 | Salmonella enterica | reduced virulence |
| gene2119 | Chromosome | VTL1 | PHI:2032 | Magnaporthe oryzae | unaffected pathogenicity |
| gene2123 | Chromosome | SP_0676 | PHI:3683 | Streptococcus pneumoniae | reduced virulence |
| gene2129 | Chromosome | FNR | PHI:4876 | Escherichia coli | reduced virulence |
| gene2133 | Chromosome | sssB | PHI:3780 | Salmonella enterica | effector (plant avirulence determinant) |
| gene2136 | Chromosome | RstA | PHI:3466 | Escherichia coli | reduced virulence |
| gene2137 | Chromosome | cpxA | PHI:8513 | Citrobacter rodentium | unaffected pathogenicity |
| gene2141 | Chromosome | GzOB019 | PHI:1579 | Fusarium graminearum | unaffected pathogenicity |
| gene2150 | Chromosome | Lmo0866 | PHI:6510 | Listeria monocytogenes | unaffected pathogenicity |
| gene2155 | Chromosome | bab2_0700 | PHI:9130 | Brucella abortus | reduced virulence |
| gene2159 | Chromosome | TyrR | PHI:4563 | Yersinia pestis | reduced virulence |
| gene2162 | Chromosome | pspD | PHI:7660 | Salmonella enterica | unaffected pathogenicity |
| gene2163 | Chromosome | pspC | PHI:7659 | Salmonella enterica | unaffected pathogenicity |
| gene2164 | Chromosome | pspB | PHI:7658 | Salmonella enterica | reduced virulence |
| gene2165 | Chromosome | pspA | PHI:7657 | Salmonella enterica | unaffected pathogenicity |
| gene2166 | Chromosome | PXO_RS01340 | PHI:9011 | Xanthomonas oryzae | unaffected pathogenicity |
| gene2170 | Chromosome | oppD (bb0334) | PHI:7820 | Borreliella burgdorferi | reduced virulence |
| gene2171 | Chromosome | oppF (bb0335) | PHI:7821 | Borreliella burgdorferi | reduced virulence |
| gene2173 | Chromosome | SoxR | PHI:3092 | Pantoea stewartii | reduced virulence |
| gene2174 | Chromosome | pafP | PHI:4631 | Escherichia coli | reduced virulence |
| gene2176 | Chromosome | ssdA (AFUB_010850) | PHI:9183 | Aspergillus fumigatus | reduced virulence |
| gene2177 | Chromosome | cstA | PHI:7577 | Salmonella enterica | reduced virulence |
| gene2179 | Chromosome | rpfR | PHI:5504 | Cronobacter turicensis | unaffected pathogenicity |
| gene2180 | Chromosome | rpfF | PHI:5503 | Cronobacter turicensis | reduced virulence |
| gene2191 | Chromosome | cysB | PHI:8764 | Pseudomonas aeruginosa | reduced virulence |
| gene2195 | Chromosome | gigX5 | PHI:6957 | Xanthomonas oryzae | increased virulence (hypervirulence) |
| gene2202 | Chromosome | trpA | PHI:7653 | Aspergillus fumigatus | reduced virulence |
| gene2203 | Chromosome | Mrtdc | PHI:7251 | Metarhizium robertsii | reduced virulence |
| gene2206 | Chromosome | trpB | PHI:9321 | Erwinia amylovora | reduced virulence |
| gene2207 | Chromosome | TRPS | PHI:2517 | Aspergillus fumigatus | lethal |
| gene2214 | Chromosome | tonB | PHI:6651 | Klebsiella pneumoniae | unaffected pathogenicity |
| gene2221 | Chromosome | oppF (bb0335) | PHI:7821 | Borreliella burgdorferi | reduced virulence |
| gene2222 | Chromosome | oppD (bb0334) | PHI:7820 | Borreliella burgdorferi | reduced virulence |
| gene2225 | Chromosome | bab2_0700 | PHI:9130 | Brucella abortus | reduced virulence |
| gene2226 | Chromosome | bab2_0700 | PHI:9130 | Brucella abortus | reduced virulence |
| gene2228 | Chromosome | adhE | PHI:8932 | Streptococcus suis | reduced virulence |
| gene2230 | Chromosome | hns | PHI:6097 | Klebsiella pneumoniae | increased virulence (hypervirulence) |
| gene2232 | Chromosome | UGD1 | PHI:387 | Cryptococcus neoformans | loss of pathogenicity |
| gene2233 | Chromosome | galU | PHI:3112 | Xanthomonas campestris | loss of pathogenicity |
| gene2234 | Chromosome | hnr | PHI:2679 | Salmonella enterica | reduced virulence |
| gene2248 | Chromosome | GAPDH | PHI:6904 | Staphylococcus aureus | reduced virulence |
| gene2250 | Chromosome | XC_0281 | PHI:3947 | Xanthomonas campestris | reduced virulence |
| gene2256 | Chromosome | cps2E | PHI:6138 | Streptococcus suis | reduced virulence |
| gene2262 | Chromosome | gntR | PHI:6702 | Vibrio cholerae | reduced virulence |
| gene2273 | Chromosome | fadD | PHI:4108 | Vibrio cholerae | unaffected pathogenicity |
| gene2274 | Chromosome | slp | PHI:9256 | Escherichia coli | reduced virulence |
| gene2275 | Chromosome | yeaZ | PHI:6870 | Vibrio harveyi | reduced virulence |
| gene2282 | Chromosome | trpA | PHI:7653 | Aspergillus fumigatus | reduced virulence |
| gene2283 | Chromosome | yeaB | PHI:9496 | Salmonella enterica | reduced virulence |
| gene2285 | Chromosome | pdeB | PHI:8643 | Erwinia amylovora | unaffected pathogenicity |
| gene2286 | Chromosome | PSPTO_2691 | PHI:3118 | Pseudomonas syringae | unaffected pathogenicity |
| gene2292 | Chromosome | cspC | PHI:7262 | Salmonella enterica | unaffected pathogenicity |
| gene2293 | Chromosome | VEDA_05199 | PHI:7709 | Verticillium dahliae | unaffected pathogenicity |
| gene2295 | Chromosome | yobH (SL1344_1770) | PHI:9521 | Salmonella enterica | reduced virulence |
| gene2296 | Chromosome | kdgR | PHI:4175 | Dickeya solani | unaffected pathogenicity |
| gene2299 | Chromosome | Prc | PHI:3894 | Xanthomonas oryzae | reduced virulence |
| gene2300 | Chromosome | ProQ | PHI:8783 | Salmonella enterica | unaffected pathogenicity |
| gene2326 | Chromosome | Oligopeptidase B | PHI:2580 | Trypanosoma cruzi | reduced virulence |
| gene2330 | Chromosome | purT | PHI:8653 | Escherichia coli | unaffected pathogenicity |
| gene2334 | Chromosome | pyk | PHI:6684 | Brucella abortus | reduced virulence |
| gene2337 | Chromosome | asbb | PHI:4079 | Bacillus cereus | unaffected pathogenicity |
| gene2340 | Chromosome | ypo2062 | PHI:4185 | Yersinia pestis | reduced virulence |
| gene2341 | Chromosome | znuA | PHI:4877 | Pseudomonas aeruginosa | reduced virulence |
| gene2342 | Chromosome | fecE | PHI:8010 | Escherichia coli | reduced virulence |
| gene2343 | Chromosome | znuB | PHI:5013 | Yersinia pseudotuberculosis | reduced virulence |
| gene2344 | Chromosome | rarA | PHI:6447 | Klebsiella pneumoniae | reduced virulence |
| gene2423 | Chromosome | flhA | PHI:8731 | Salmonella enterica | reduced virulence |
| gene2424 | Chromosome | sctU | PHI:4470 | Burkholderia pseudomallei | reduced virulence |
| gene2426 | Chromosome | cheY | PHI:8732 | Salmonella enterica | reduced virulence |
| gene2427 | Chromosome | cheB | PHI:6461 | Salmonella enterica | reduced virulence |
| gene2428 | Chromosome | cheR1 | PHI:9031 | Pseudomonas aeruginosa | reduced virulence |
| gene2429 | Chromosome | tsr | PHI:6981 | Salmonella enterica | reduced virulence |
| gene2430 | Chromosome | tsr | PHI:6981 | Salmonella enterica | reduced virulence |
| gene2431 | Chromosome | tsr | PHI:6981 | Salmonella enterica | reduced virulence |
| gene2432 | Chromosome | cheW | PHI:6536 | Escherichia coli | unaffected pathogenicity |
| gene2433 | Chromosome | cheA | PHI:6535 | Escherichia coli | unaffected pathogenicity |
| gene2434 | Chromosome | motB | PHI:6534 | Escherichia coli | reduced virulence |
| gene2435 | Chromosome | motA | PHI:6361 | Pantoea ananatis | reduced virulence |
| gene2436 | Chromosome | flhC (Dda3937_02784) | PHI:8572 | Dickeya dadantii | reduced virulence |
| gene2437 | Chromosome | flhD | PHI:4075 | Serratia marcescens | unaffected pathogenicity |
| gene2438 | Chromosome | otsA | PHI:4144 | Xanthomonas citri | reduced virulence |
| gene2440 | Chromosome | VCA0681 | PHI:3227 | Vibrio cholerae | unaffected pathogenicity |
| gene2453 | Chromosome | XC_2801 | PHI:3260 | Xanthomonas campestris | reduced virulence |
| gene2454 | Chromosome | fabG1 | PHI:5271 | Ralstonia solanacearum | lethal |
| gene2455 | Chromosome | XC_2801 | PHI:3260 | Xanthomonas campestris | reduced virulence |
| gene2458 | Chromosome | Abd1 | PHI:2255 | Parastagonospora nodorum | unaffected pathogenicity |
| gene2460 | Chromosome | leuO | PHI:7770 | Vibrio cholerae | reduced virulence |
| gene2468 | Chromosome | MorA | PHI:4684 | Pseudomonas aeruginosa | increased virulence (hypervirulence) |
| gene2469 | Chromosome | AldB (PSPTO_2673) | PHI:7788 | Pseudomonas syringae | reduced virulence |
| gene2481 | Chromosome | XacFhaB | PHI:5388 | Xanthomonas citri | reduced virulence |
| gene2490 | Chromosome | putA | PHI:7999 | Pseudomonas aeruginosa | reduced virulence |
| gene2493 | Chromosome | cab (NMB0789) | PHI:8623 | Neisseria meningitidis | unaffected pathogenicity |
| gene2494 | Chromosome | ctp (NMB0788) | PHI:8622 | Neisseria meningitidis | unaffected pathogenicity |
| gene2495 | Chromosome | cbp (NMB0787) | PHI:8621 | Neisseria meningitidis | unaffected pathogenicity |
| gene2496 | Chromosome | ACC deaminase (VDAG_10392) | PHI:8670 | Verticillium dahliae | reduced virulence |
| gene2497 | Chromosome | FliZ | PHI:3922 | Xenorhabdus nematophila | reduced virulence |
| gene2498 | Chromosome | rpoS | PHI:3336 | Erwinia amylovora | unaffected pathogenicity |
| gene2500 | Chromosome | FliC | PHI:2642 | Edwardsiella tarda | reduced virulence |
| gene2501 | Chromosome | fliD | PHI:6733 | Escherichia coli | unaffected pathogenicity |
| gene2507 | Chromosome | bpdA | PHI:3645 | Brucella melitensis | reduced virulence |
| gene2524 | Chromosome | hopA1 | PHI:3165 | Pseudomonas cichorii | unaffected pathogenicity |
| gene2568 | Chromosome | PspK | PHI:6281 | Streptococcus pneumoniae | reduced virulence |
| gene2586 | Chromosome | Flil | PHI:6995 | Pseudomonas aeruginosa | reduced virulence |
| gene2587 | Chromosome | filj | PHI:6463 | Salmonella enterica | reduced virulence |
| gene2590 | Chromosome | fliM | PHI:6194 | Leptospira interrogans | reduced virulence |
| gene2593 | Chromosome | yscR | PHI:627 | Salmonella enterica | reduced virulence |
| gene2594 | Chromosome | ssaS | PHI:628 | Salmonella enterica | reduced virulence |
| gene2596 | Chromosome | rcsA | PHI:4478 | Erwinia amylovora | loss of pathogenicity |
| gene2597 | Chromosome | trg | PHI:6982 | Salmonella enterica | reduced virulence |
| gene2603 | Chromosome | bcam2836 | PHI:8835 | Burkholderia cenocepacia | increased virulence (hypervirulence) |
| gene2611 | Chromosome | tsr | PHI:6981 | Salmonella enterica | reduced virulence |
| gene2617 | Chromosome | cspE | PHI:7263 | Salmonella enterica | unaffected pathogenicity |
| gene2624 | Chromosome | vtlR | PHI:5012 | Brucella abortus | reduced virulence |
| gene2625 | Chromosome | YHB1 | PHI:500 | Candida albicans | reduced virulence |
| gene2630 | Chromosome | potA | PHI:6319 | Streptococcus pneumoniae | reduced virulence |
| gene2636 | Chromosome | entA | PHI:4077 | Bacillus cereus | reduced virulence |
| gene2642 | Chromosome | RNase E | PHI:3723 | Salmonella enterica | reduced virulence |
| gene2644 | Chromosome | flgK | PHI:6360 | Pantoea ananatis | reduced virulence |
| gene2662 | Chromosome | pyrC | PHI:9307 | Erwinia amylovora | reduced virulence |
| gene2669 | Chromosome | PA0423 | PHI:3166 | Pseudomonas aeruginosa | reduced virulence |
| gene2670 | Chromosome | spr0084 | PHI:3154 | Streptococcus pneumoniae | reduced virulence |
| gene2672 | Chromosome | lpxL1 (BN49_2155) | PHI:7946 | Klebsiella pneumoniae | unaffected pathogenicity |
| gene2675 | Chromosome | HrpM | PHI:2699 | Xanthomonas citri | loss of pathogenicity |
| gene2676 | Chromosome | opgG | PHI:5564 | Dickeya dadantii | reduced virulence |
| gene2678 | Chromosome | PLD2 | PHI:5093 | Acinetobacter baumannii | reduced virulence |
| gene2680 | Chromosome | KSA1 | PHI:724 | Fusarium graminearum | unaffected pathogenicity |
| gene2681 | Chromosome | fabG2 | PHI:5272 | Ralstonia solanacearum | reduced virulence |
| gene2685 | Chromosome | z4861 | PHI:9197 | Escherichia coli | reduced virulence |
| gene2696 | Chromosome | adeK | PHI:6388 | Acinetobacter baumannii | unaffected pathogenicity |
| gene2697 | Chromosome | oqxA | PHI:6448 | Klebsiella pneumoniae | reduced virulence |
| gene2709 | Chromosome | sirA | PHI:560 | Salmonella enterica | reduced virulence |
| gene2713 | Chromosome | RhlR | PHI:7289 | Pseudomonas aeruginosa | loss of pathogenicity |
| gene2724 | Chromosome | BbbqrA | PHI:4917 | Beauveria bassiana | unaffected pathogenicity |
| gene2726 | Chromosome | Sdh1 | PHI:3914 | Parastagonospora nodorum | reduced virulence |
| gene2748 | Chromosome | OmpA2 | PHI:4160 | Klebsiella pneumoniae | reduced virulence |
| gene2750 | Chromosome | LonC | PHI:5467 | Actinobacillus pleuropneumoniae | unaffected pathogenicity |
| gene2755 | Chromosome | raxB | PHI:1141 | Xanthomonas oryzae | increased virulence (hypervirulence) |
| gene2764 | Chromosome | proV | PHI:8968 | Klebsiella pneumoniae | reduced virulence |
| gene2767 | Chromosome | GzOB046 | PHI:1605 | Fusarium graminearum | unaffected pathogenicity |
| gene2768 | Chromosome | ompF | PHI:7023 | Escherichia coli | reduced virulence |
| gene2769 | Chromosome | PsAAT3 | PHI:6261 | Phytophthora sojae | reduced virulence |
| gene2783 | Chromosome | ABC3 | PHI:1018 | Magnaporthe oryzae | loss of pathogenicity |
| gene2785 | Chromosome | himD | PHI:2673 | Salmonella enterica | reduced virulence |
| gene2786 | Chromosome | pnp | PHI:3878 | Salmonella enterica | reduced virulence |
| gene2788 | Chromosome | aroA | PHI:9459 | Salmonella enterica | reduced virulence |
| gene2796 | Chromosome | rarA | PHI:6447 | Klebsiella pneumoniae | reduced virulence |
| gene2798 | Chromosome | essC (lmo0061) | PHI:7805 | Listeria monocytogenes | increased virulence (hypervirulence) |
| gene2799 | Chromosome | lrp | PHI:9339 | Erwinia amylovora | unaffected pathogenicity |
| gene2800 | Chromosome | Trr1 | PHI:6470 | Beauveria bassiana | unaffected pathogenicity |
| gene2801 | Chromosome | ABC3 | PHI:1018 | Magnaporthe oryzae | loss of pathogenicity |
| gene2802 | Chromosome | iroC | PHI:9461 | Salmonella enterica | reduced virulence |
| gene2805 | Chromosome | clpA (Dda3937_02363) | PHI:8569 | Dickeya dadantii | reduced virulence |
| gene2806 | Chromosome | clpS | PHI:8568 | Dickeya dadantii | reduced virulence |
| gene2807 | Chromosome | CspD ( WP_003488188.1) | PHI:8639 | Xanthomonas oryzae | unaffected pathogenicity |
| gene2810 | Chromosome | TOXG | PHI:469 | Bipolaris zeicola | reduced virulence |
| gene2814 | Chromosome | cab (NMB0789) | PHI:8623 | Neisseria meningitidis | unaffected pathogenicity |
| gene2815 | Chromosome | bab2_0612 | PHI:7201 | Brucella abortus | reduced virulence |
| gene2816 | Chromosome | ctp (NMB0788) | PHI:8622 | Neisseria meningitidis | unaffected pathogenicity |
| gene2818 | Chromosome | bab2_0612 | PHI:7201 | Brucella abortus | reduced virulence |
| gene2824 | Chromosome | potC | PHI:6321 | Streptococcus pneumoniae | reduced virulence |
| gene2826 | Chromosome | potA | PHI:6319 | Streptococcus pneumoniae | reduced virulence |
| gene2849 | Chromosome | oppF (bb0335) | PHI:7821 | Borreliella burgdorferi | reduced virulence |
| gene2853 | Chromosome | bcam0748 | PHI:8829 | Burkholderia cenocepacia | increased virulence (hypervirulence) |
| gene2854 | Chromosome | FepC (VK055_1930) | PHI:7541 | Klebsiella pneumoniae | reduced virulence |
| gene2855 | Chromosome | CBU_0347 | PHI:9510 | Coxiella burnetii | unaffected pathogenicity |
| gene2856 | Chromosome | adeK | PHI:6388 | Acinetobacter baumannii | unaffected pathogenicity |
| gene2860 | Chromosome | mntR | PHI:9035 | Staphylococcus aureus | unaffected pathogenicity |
| gene2862 | Chromosome | OmpX | PHI:6250 | Escherichia coli | reduced virulence |
| gene2866 | Chromosome | bab2_0612 | PHI:7201 | Brucella abortus | reduced virulence |
| gene2868 | Chromosome | cab (NMB0789) | PHI:8623 | Neisseria meningitidis | unaffected pathogenicity |
| gene2875 | Chromosome | agaR1 | PHI:5301 | Streptococcus suis | unaffected pathogenicity |
| gene2885 | Chromosome | ydcN | PHI:2458 | Erwinia amylovora | reduced virulence |
| gene2886 | Chromosome | orf242 | PHI:602 | Salmonella enterica | unaffected pathogenicity |
| gene2891 | Chromosome | proV | PHI:8968 | Klebsiella pneumoniae | reduced virulence |
| gene2896 | Chromosome | cheM | PHI:6983 | Salmonella enterica | reduced virulence |
| gene2899 | Chromosome | TOM1 | PHI:191 | Septoria lycopersici | unaffected pathogenicity |
| gene2905 | Chromosome | fabG1 | PHI:5271 | Ralstonia solanacearum | lethal |
| gene2909 | Chromosome | PitA | PHI:9451 | Staphylococcus aureus | unaffected pathogenicity |
| gene2910 | Chromosome | Rv3232c | PHI:3634 | Mycobacterium tuberculosis | reduced virulence |
| gene2913 | Chromosome | Lmo0866 | PHI:6510 | Listeria monocytogenes | unaffected pathogenicity |
| gene2923 | Chromosome | cheM | PHI:6983 | Salmonella enterica | reduced virulence |
| gene2927 | Chromosome | rv3111 | PHI:7748 | Mycobacterium tuberculosis | unaffected pathogenicity |
| gene2932 | Chromosome | iroC | PHI:9461 | Salmonella enterica | reduced virulence |
| gene2937 | Chromosome | argD | PHI:3126 | Erwinia amylovora | unaffected pathogenicity |
| gene2938 | Chromosome | RED1 | PHI:2839 | Bipolaris maydis | reduced virulence |
| gene2939 | Chromosome | GLX3 | PHI:8693 | Candida albicans | loss of pathogenicity |
| gene2943 | Chromosome | potA | PHI:6319 | Streptococcus pneumoniae | reduced virulence |
| gene2945 | Chromosome | modA10 | PHI:5422 | Haemophilus influenzae | increased virulence (hypervirulence) |
| gene2948 | Chromosome | ABC3 | PHI:1018 | Magnaporthe oryzae | loss of pathogenicity |
| gene2950 | Chromosome | galK | PHI:6269 | Escherichia coli | reduced virulence |
| gene2953 | Chromosome | aroG1 | PHI:8884 | Ralstonia solanacearum | loss of pathogenicity |
| gene2955 | Chromosome | ssrB | PHI:6965 | Salmonella enterica | unaffected pathogenicity |
| gene2956 | Chromosome | BvgS | PHI:8545 | Bordetella pertussis | unaffected pathogenicity |
| gene2966 | Chromosome | cadF | PHI:9291 | Campylobacter jejuni | unaffected pathogenicity |
| gene2967 | Chromosome | TolB | PHI:3137 | Pseudomonas aeruginosa | reduced virulence |
| gene2968 | Chromosome | TolA | PHI:3472 | Escherichia coli | reduced virulence |
| gene2978 | Chromosome | odhB | PHI:7353 | Staphylococcus aureus | unaffected pathogenicity |
| gene2979 | Chromosome | VdOGDH (VDAG_10018) | PHI:9378 | Verticillium dahliae | reduced virulence |
| gene2980 | Chromosome | sdhB | PHI:7965 | Escherichia coli | reduced virulence |
| gene2981 | Chromosome | sdhA | PHI:7964 | Escherichia coli | reduced virulence |
| gene2982 | Chromosome | sdhD | PHI:7963 | Escherichia coli | reduced virulence |
| gene2983 | Chromosome | sdhC | PHI:7962 | Escherichia coli | reduced virulence |
| gene2993 | Chromosome | phr1 (EJP69699) | PHI:8671 | Beauveria bassiana | unaffected pathogenicity |
| gene2996 | Chromosome | kdpE | PHI:6314 | Pseudomonas syringae | increased virulence (hypervirulence) |
| gene3002 | Chromosome | Fur | PHI:4887 | Escherichia coli | unaffected pathogenicity |
| gene3003 | Chromosome | GUS1 | PHI:2521 | Aspergillus fumigatus | lethal |
| gene3004 | Chromosome | glcA | PHI:6308 | Staphylococcus aureus | reduced virulence |
| gene3005 | Chromosome | MoDeam | PHI:5472 | Magnaporthe oryzae | reduced virulence |
| gene3006 | Chromosome | MoDac | PHI:5471 | Magnaporthe oryzae | reduced virulence |
| gene3009 | Chromosome | asnB | PHI:9327 | Erwinia amylovora | unaffected pathogenicity |
| gene3017 | Chromosome | ubiI | PHI:7028 | Escherichia coli | reduced virulence |
| gene3020 | Chromosome | ybeY | PHI:7671 | Yersinia enterocolitica | reduced virulence |
| gene3022 | Chromosome | PA3984 | PHI:3782 | Pseudomonas aeruginosa | reduced virulence |
| gene3026 | Chromosome | cab (NMB0789) | PHI:8623 | Neisseria meningitidis | unaffected pathogenicity |
| gene3036 | Chromosome | PA4485 | PHI:5448 | Pseudomonas aeruginosa | unaffected pathogenicity |
| gene3040 | Chromosome | lipA | PHI:6492 | Acinetobacter baumannii | reduced virulence |
| gene3041 | Chromosome | tatA | PHI:8757 | Salmonella enterica | reduced virulence |
| gene3042 | Chromosome | cab (NMB0789) | PHI:8623 | Neisseria meningitidis | unaffected pathogenicity |
| gene3049 | Chromosome | cspE | PHI:7263 | Salmonella enterica | unaffected pathogenicity |
| gene3050 | Chromosome | pagP | PHI:7956 | Klebsiella pneumoniae | unaffected pathogenicity |
| gene3054 | Chromosome | hrpS | PHI:3671 | Erwinia amylovora | loss of pathogenicity |
| gene3056 | Chromosome | BP1026B_II1646 | PHI:5331 | Burkholderia pseudomallei | effector (plant avirulence determinant) |
| gene3061 | Chromosome | hrcC | PHI:6727 | Pseudomonas syringae | increased virulence (hypervirulence) |
| gene3070 | Chromosome | HrcU | PHI:6338 | Erwinia amylovora | loss of pathogenicity |
| gene3072 | Chromosome | ssaS | PHI:628 | Salmonella enterica | reduced virulence |
| gene3073 | Chromosome | yscR | PHI:627 | Salmonella enterica | reduced virulence |
| gene3077 | Chromosome | yscN | PHI:7902 | Yersinia pseudotuberculosis | unaffected pathogenicity |
| gene3079 | Chromosome | ascV | PHI:6829 | Aeromonas salmonicida | reduced virulence |
| gene3080 | Chromosome | hrpJ | PHI:2468 | Erwinia amylovora | reduced virulence |
| gene3081 | Chromosome | hrpL | PHI:5281 | Erwinia amylovora | reduced virulence |
| gene3082 | Chromosome | hrpx | PHI:2473 | Pantoea stewartii | reduced virulence |
| gene3083 | Chromosome | BceR | PHI:3451 | Burkholderia cenocepacia | unaffected pathogenicity |
| gene3086 | Chromosome | gntU | PHI:6700 | Vibrio cholerae | reduced virulence |
| gene3094 | Chromosome | raxB | PHI:1141 | Xanthomonas oryzae | increased virulence (hypervirulence) |
| gene3095 | Chromosome | XC_2659 | PHI:9170 | Xanthomonas campestris | unaffected pathogenicity |
| gene3096 | Chromosome | fiuA | PHI:6373 | Pseudomonas aeruginosa | reduced virulence |
| gene3098 | Chromosome | adhE | PHI:8932 | Streptococcus suis | reduced virulence |
| gene3100 | Chromosome | ovrB (Z0346) | PHI:9508 | Escherichia coli | reduced virulence |
| gene3104 | Chromosome | WtsE | PHI:6376 | Pantoea stewartii | effector (plant avirulence determinant) |
| gene3112 | Chromosome | PXO_RS11880/ PXO_RS12810 | PHI:8985 | Xanthomonas oryzae | unaffected pathogenicity |
| gene3113 | Chromosome | FVEG_12531 | PHI:3383 | Fusarium verticillioides | unaffected pathogenicity |
| gene3114 | Chromosome | pdhR | PHI:3135 | Yersinia pseudotuberculosis | reduced virulence |
| gene3117 | Chromosome | entA | PHI:4077 | Bacillus cereus | reduced virulence |
| gene3121 | Chromosome | Mqo | PHI:6190 | Staphylococcus aureus | reduced virulence |
| gene3128 | Chromosome | THR | PHI:2312 | Alternaria alternata | unaffected pathogenicity |
| gene3129 | Chromosome | Abd1 | PHI:2255 | Parastagonospora nodorum | unaffected pathogenicity |
| gene3132 | Chromosome | xrp8 | PHI:4137 | Xanthomonas oryzae | effector (plant avirulence determinant) |
| gene3133 | Chromosome | katN | PHI:7042 | Escherichia coli | effector (plant avirulence determinant) |
| gene3146 | Chromosome | XC_0281 | PHI:3947 | Xanthomonas campestris | reduced virulence |
| gene3153 | Chromosome | glcA | PHI:6308 | Staphylococcus aureus | reduced virulence |
| gene3154 | Chromosome | CcpA | PHI:4564 | Enterococcus faecium | reduced virulence |
| gene3158 | Chromosome | ppiB | PHI:7633 | Pseudomonas aeruginosa | reduced virulence |
| gene3160 | Chromosome | ADE2 | PHI:501 | Saccharomyces cerevisiae | reduced virulence |
| gene3161 | Chromosome | ADE2 | PHI:14 | Cryptococcus neoformans | reduced virulence |
| gene3162 | Chromosome | expR | PHI:4178 | Dickeya solani | unaffected pathogenicity |
| gene3163 | Chromosome | expI | PHI:5535 | Pectobacterium carotovorum | reduced virulence |
| gene3165 | Chromosome | MacB | PHI:3928 | Salmonella enterica | reduced virulence |
| gene3174 | Chromosome | copA | PHI:5275 | Vibrio tasmaniensis | reduced virulence |
| gene3177 | Chromosome | ovrB (Z0346) | PHI:9508 | Escherichia coli | reduced virulence |
| gene3183 | Chromosome | hemH | PHI:4734 | Francisella tularensis | unaffected pathogenicity |
| gene3189 | Chromosome | lhnR | PHI:3491 | Agrobacterium vitis | reduced virulence |
| gene3191 | Chromosome | htpG | PHI:6472 | Escherichia coli | reduced virulence |
| gene3199 | Chromosome | rsmS (ECA1172) | PHI:8933 | Pectobacterium atrosepticum | increased virulence (hypervirulence) |
| gene3201 | Chromosome | adeI | PHI:6386 | Acinetobacter baumannii | unaffected pathogenicity |
| gene3202 | Chromosome | AcrB | PHI:2469 | Erwinia amylovora | reduced virulence |
| gene3207 | Chromosome | hmuV | PHI:7909 | Yersinia pseudotuberculosis | unaffected pathogenicity |
| gene3210 | Chromosome | ymoA | PHI:3819 | Yersinia pestis | increased virulence (hypervirulence) |
| gene3216 | Chromosome | ABC3 | PHI:1018 | Magnaporthe oryzae | loss of pathogenicity |
| gene3217 | Chromosome | ybtP | PHI:6912 | Escherichia coli | reduced virulence |
| gene3226 | Chromosome | hupB (XAC1081) | PHI:8676 | Xanthomonas citri | reduced virulence |
| gene3227 | Chromosome | lon | PHI:7160 | Erwinia amylovora | increased virulence (hypervirulence) |
| gene3228 | Chromosome | ClpX | PHI:3004 | Staphylococcus aureus | reduced virulence |
| gene3229 | Chromosome | ClpP | PHI:3040 | Salmonella enterica | reduced virulence |
| gene3235 | Chromosome | XAC1258 | PHI:4147 | Xanthomonas citri | reduced virulence |
| gene3236 | Chromosome | qoxB | PHI:4038 | Staphylococcus aureus | reduced virulence |
| gene3241 | Chromosome | XC_3703 | PHI:3259 | Xanthomonas campestris | effector (plant avirulence determinant) |
| gene3250 | Chromosome | CSH1 | PHI:419 | Candida albicans | reduced virulence |
| gene3256 | Chromosome | nrdR | PHI:3464 | Streptococcus pyogenes | unaffected pathogenicity |
| gene3265 | Chromosome | TSA1 | PHI:386 | Cryptococcus neoformans | reduced virulence |
| gene3268 | Chromosome | brnQ1 | PHI:6629 | Staphylococcus aureus | unaffected pathogenicity |
| gene3271 | Chromosome | phoR | PHI:9434 | Salmonella enterica | reduced virulence |
| gene3272 | Chromosome | PhoB | PHI:8032 | Xanthomonas oryzae | unaffected pathogenicity |
| gene3296 | Chromosome | lacZ | PHI:6268 | Escherichia coli | reduced virulence |
| gene3298 | Chromosome | cspE | PHI:7263 | Salmonella enterica | unaffected pathogenicity |
| gene3302 | Chromosome | MorA | PHI:4684 | Pseudomonas aeruginosa | increased virulence (hypervirulence) |
| gene3307 | Chromosome | oppD (bb0334) | PHI:7820 | Borreliella burgdorferi | reduced virulence |
| gene3308 | Chromosome | oppF (bb0335) | PHI:7821 | Borreliella burgdorferi | reduced virulence |
| gene3318 | Chromosome | acrB | PHI:6451 | Klebsiella pneumoniae | reduced virulence |
| gene3319 | Chromosome | Ss-odc2 | PHI:4509 | Sclerotinia sclerotiorum | reduced virulence |
| gene3323 | Chromosome | CBU_0347 | PHI:9510 | Coxiella burnetii | unaffected pathogenicity |
| gene3328 | Chromosome | lacA | PHI:3526 | Salmonella enterica | reduced virulence |
| gene3334 | Chromosome | treY | PHI:2745 | Pseudomonas aeruginosa | unaffected pathogenicity |
| gene3335 | Chromosome | XC_0423 | PHI:3958 | Xanthomonas campestris | reduced virulence |
| gene3340 | Chromosome | ompF | PHI:7023 | Escherichia coli | reduced virulence |
| gene3354 | Chromosome | mcpB | PHI:6985 | Salmonella enterica | reduced virulence |
| gene3362 | Chromosome | yedX | PHI:2651 | Salmonella enterica | unaffected pathogenicity |
| gene3366 | Chromosome | cab (NMB0789) | PHI:8623 | Neisseria meningitidis | unaffected pathogenicity |
| gene3373 | Chromosome | MCC | PHI:5068 | Fusarium graminearum | reduced virulence |
| gene3389 | Chromosome | ipx10 | PHI:2975 | Pseudomonas syringae | effector (plant avirulence determinant) |
| gene3394 | Chromosome | XC_2801 | PHI:3260 | Xanthomonas campestris | reduced virulence |
| gene3402 | Chromosome | proV | PHI:8968 | Klebsiella pneumoniae | reduced virulence |
| gene3404 | Chromosome | metQ | PHI:2732 | Streptococcus pneumoniae | unaffected pathogenicity |
| gene3416 | Chromosome | speC (RSc2365) | PHI:7723 | Ralstonia solanacearum | loss of pathogenicity |
| gene3417 | Chromosome | VcpD | PHI:702 | Vibrio cholerae | reduced virulence |
| gene3422 | Chromosome | PA3644 | PHI:3785 | Pseudomonas aeruginosa | reduced virulence |
| gene3424 | Chromosome | PA3646 | PHI:3784 | Pseudomonas aeruginosa | reduced virulence |
| gene3427 | Chromosome | PXO_01122 | PHI:3895 | Xanthomonas oryzae | reduced virulence |
| gene3435 | Chromosome | map | PHI:9334 | Citrobacter rodentium | effector (plant avirulence determinant) |
| gene3440 | Chromosome | degP (ROD_01651) | PHI:8690 | Citrobacter rodentium | unaffected pathogenicity |
| gene3446 | Chromosome | CLC-A | PHI:286 | Cryptococcus neoformans | reduced virulence |
| gene3447 | Chromosome | XC_0892 | PHI:3950 | Xanthomonas campestris | reduced virulence |
| gene3448 | Chromosome | fecC | PHI:8008 | Escherichia coli | reduced virulence |
| gene3450 | Chromosome | FepC (VK055_1930) | PHI:7541 | Klebsiella pneumoniae | reduced virulence |
| gene3451 | Chromosome | fiuA | PHI:6373 | Pseudomonas aeruginosa | reduced virulence |
| gene3453 | Chromosome | GzOB019 | PHI:1579 | Fusarium graminearum | unaffected pathogenicity |
| gene3456 | Chromosome | DksA | PHI:6508 | Salmonella enterica | reduced virulence |
| gene3461 | Chromosome | Pbl1 | PHI:2257 | Parastagonospora nodorum | unaffected pathogenicity |
| gene3462 | Chromosome | XC_2466 | PHI:3968 | Xanthomonas campestris | reduced virulence |
| gene3466 | Chromosome | ABC4 | PHI:1017 | Magnaporthe oryzae | reduced virulence |
| gene3467 | Chromosome | Can1 | PHI:673 | Cryptococcus neoformans | unaffected pathogenicity |
| gene3468 | Chromosome | XhpT | PHI:2936 | Xylella fastidiosa | reduced virulence |
| gene3469 | Chromosome | metB (Dda3937_03888) | PHI:8570 | Dickeya dadantii | reduced virulence |
| gene3473 | Chromosome | speE | PHI:9153 | Escherichia coli | unaffected pathogenicity |
| gene3474 | Chromosome | speD | PHI:9152 | Escherichia coli | unaffected pathogenicity |
| gene3478 | Chromosome | adhE | PHI:8932 | Streptococcus suis | reduced virulence |
| gene3484 | Chromosome | vtlR | PHI:5012 | Brucella abortus | reduced virulence |
| gene3489 | Chromosome | oppD (bb0334) | PHI:7820 | Borreliella burgdorferi | reduced virulence |
| gene3490 | Chromosome | oppF (bb0335) | PHI:7821 | Borreliella burgdorferi | reduced virulence |
| gene3497 | Chromosome | LpdA | PHI:6520 | Legionella pneumophila | effector (plant avirulence determinant) |
| gene3500 | Chromosome | pdhR | PHI:3135 | Yersinia pseudotuberculosis | reduced virulence |
| gene3507 | Chromosome | pilA | PHI:6362 | Pantoea ananatis | reduced virulence |
| gene3508 | Chromosome | pilT | PHI:6363 | Pantoea ananatis | reduced virulence |
| gene3510 | Chromosome | IMPDH | PHI:4071 | Streptococcus suis | reduced virulence |
| gene3515 | Chromosome | secA | PHI:5240 | Acinetobacter baumannii | reduced virulence |
| gene3518 | Chromosome | wcbS | PHI:5346 | Burkholderia pseudomallei | reduced virulence |
| gene3526 | Chromosome | murD | PHI:7631 | Pseudomonas aeruginosa | reduced virulence |
| gene3528 | Chromosome | murF | PHI:7632 | Pseudomonas aeruginosa | unaffected pathogenicity |
| gene3532 | Chromosome | rsmH | PHI:4961 | Staphylococcus aureus | reduced virulence |
| gene3535 | Chromosome | fruR | PHI:2672 | Salmonella enterica | reduced virulence |
| gene3536 | Chromosome | MoIlv6 | PHI:3976 | Magnaporthe oryzae | loss of pathogenicity |
| gene3537 | Chromosome | MoIlv2 | PHI:3975 | Magnaporthe oryzae | loss of pathogenicity |
| gene3538 | Chromosome | leuA | PHI:8962 | Klebsiella pneumoniae | reduced virulence |
| gene3539 | Chromosome | leuB | PHI:9319 | Erwinia amylovora | reduced virulence |
| gene3540 | Chromosome | leuC | PHI:9329 | Erwinia amylovora | reduced virulence |
| gene3541 | Chromosome | leuD | PHI:8965 | Klebsiella pneumoniae | reduced virulence |
| gene3546 | Chromosome | potA | PHI:6319 | Streptococcus pneumoniae | reduced virulence |
| gene3555 | Chromosome | RsmA | PHI:2735 | Pectobacterium wasabiae | increased virulence (hypervirulence) |
| gene3560 | Chromosome | phoN | PHI:8756 | Salmonella enterica | unaffected pathogenicity |
| gene3562 | Chromosome | carA | PHI:9328 | Erwinia amylovora | reduced virulence |
| gene3570 | Chromosome | nhaR | PHI:3267 | Escherichia coli | reduced virulence |
| gene3571 | Chromosome | nhaA | PHI:3266 | Escherichia coli | reduced virulence |
| gene3572 | Chromosome | DnaJ | PHI:6986 | Streptococcus pneumoniae | reduced virulence |
| gene3582 | Chromosome | FVEG_12521 | PHI:3389 | Fusarium verticillioides | unaffected pathogenicity |
| gene3584 | Chromosome | arcA | PHI:6532 | Escherichia coli | reduced virulence |
| gene3586 | Chromosome | MarA | PHI:5369 | Salmonella enterica | reduced virulence |
| gene3591 | Chromosome | MCC | PHI:5068 | Fusarium graminearum | reduced virulence |
| gene3592 | Chromosome | MCC | PHI:5068 | Fusarium graminearum | reduced virulence |
| gene3594 | Chromosome | bab2_0822 | PHI:9128 | Brucella abortus | reduced virulence |
| gene3597 | Chromosome | hmuV | PHI:7909 | Yersinia pseudotuberculosis | unaffected pathogenicity |
| gene3598 | Chromosome | pstB | PHI:3412 | Xanthomonas citri | loss of pathogenicity |
| gene3599 | Chromosome | PrdR | PHI:9139 | Clostridioides difficile | increased virulence (hypervirulence) |
| gene3601 | Chromosome | bab2_1109 | PHI:9129 | Brucella abortus | reduced virulence |
| gene3604 | Chromosome | orf408 | PHI:612 | Salmonella enterica | reduced virulence |
| gene3608 | Chromosome | virB (lmo1747) | PHI:9216 | Listeria monocytogenes | unaffected pathogenicity |
| gene3612 | Chromosome | aphB | PHI:5548 | Vibrio cholerae | reduced virulence |
| gene3613 | Chromosome | VEDA_05198 | PHI:7708 | Verticillium dahliae | unaffected pathogenicity |
| gene3641 | Chromosome | bcal2852 | PHI:8828 | Burkholderia cenocepacia | increased virulence (hypervirulence) |
| gene3651 | Chromosome | CcpA | PHI:4564 | Enterococcus faecium | reduced virulence |
| gene3654 | Chromosome | ovrB (Z0346) | PHI:9508 | Escherichia coli | reduced virulence |
| gene3668 | Chromosome | Can2 | PHI:674 | Cryptococcus neoformans | unaffected pathogenicity |
| gene3692 | Chromosome | rsmI | PHI:4960 | Staphylococcus aureus | reduced virulence |
| gene3712 | Chromosome | tcp | PHI:6980 | Salmonella enterica | reduced virulence |
| gene3724 | Chromosome | cpdB | PHI:7020 | Escherichia coli | reduced virulence |
| gene3733 | Chromosome | tsr | PHI:6981 | Salmonella enterica | reduced virulence |
| gene3737 | Chromosome | ssdA (AFUB_010850) | PHI:9183 | Aspergillus fumigatus | reduced virulence |
| gene3739 | Chromosome | purA | PHI:2625 | Salmonella enterica | reduced virulence |
| gene3744 | Chromosome | hfq | PHI:6403 | Yersinia enterocolitica | reduced virulence |
| gene3745 | Chromosome | CKS1 | PHI:6158 | Magnaporthe oryzae | reduced virulence |
| gene3746 | Chromosome | mlh1 | PHI:7208 | Cryptococcus neoformans | unaffected pathogenicity |
| gene3747 | Chromosome | amiC | PHI:9372 | Yersinia pestis | unaffected pathogenicity |
| gene3754 | Chromosome | Orn | PHI:6274 | Pseudomonas aeruginosa | reduced virulence |
| gene3759 | Chromosome | frdA | PHI:2960 | Edwardsiella ictaluri | reduced virulence |
| gene3766 | Chromosome | EF-P | PHI:6420 | Salmonella enterica | increased virulence (hypervirulence) |
| gene3769 | Chromosome | GroEL | PHI:3085 | Porphyromonas gingivalis | increased virulence (hypervirulence) |
| gene3775 | Chromosome | XC_0531 | PHI:7255 | Xanthomonas campestris | reduced virulence |
| gene3781 | Chromosome | PA0423 | PHI:3166 | Pseudomonas aeruginosa | reduced virulence |
| gene3793 | Chromosome | bab2_0612 | PHI:7201 | Brucella abortus | reduced virulence |
| gene3794 | Chromosome | treA | PHI:6630 | Xanthomonas citri | increased virulence (hypervirulence) |
| gene3796 | Chromosome | XC_2801 | PHI:3260 | Xanthomonas campestris | reduced virulence |
| gene3800 | Chromosome | XC_2801 | PHI:3260 | Xanthomonas campestris | reduced virulence |
| gene3806 | Chromosome | bcam1160 | PHI:8840 | Burkholderia cenocepacia | increased virulence (hypervirulence) |
| gene3810 | Chromosome | aer | PHI:6979 | Salmonella enterica | reduced virulence |
| gene3812 | Chromosome | acs | PHI:9227 | Escherichia coli | unaffected pathogenicity |
| gene3813 | Chromosome | yjcH | PHI:9228 | Escherichia coli | unaffected pathogenicity |
| gene3814 | Chromosome | actP | PHI:9229 | Escherichia coli | unaffected pathogenicity |
| gene3818 | Chromosome | OxyR | PHI:5305 | Pseudomonas syringae | reduced virulence |
| gene3821 | Chromosome | Smp | PHI:7699 | Pseudomonas syringae | unaffected pathogenicity |
| gene3822 | Chromosome | OxyR | PHI:3091 | Pantoea stewartii | unaffected pathogenicity |
| gene3823 | Chromosome | ramA (VK055_1983) | PHI:7475 | Klebsiella pneumoniae | unaffected pathogenicity |
| gene3824 | Chromosome | yfiN | PHI:7571 | Klebsiella pneumoniae | unaffected pathogenicity |
| gene3831 | Chromosome | ybjX | PHI:9384 | Escherichia coli | unaffected pathogenicity |
| gene3834 | Chromosome | NWMN_0826 | PHI:7917 | Staphylococcus aureus | reduced virulence |
| gene3836 | Chromosome | GLX3 | PHI:8693 | Candida albicans | loss of pathogenicity |
| gene3841 | Chromosome | MoHYR1 | PHI:2153 | Magnaporthe oryzae | reduced virulence |
| gene3846 | Chromosome | XC_2801 | PHI:3260 | Xanthomonas campestris | reduced virulence |
| gene3847 | Chromosome | PidS | PHI:2439 | Burkholderia glumae | reduced virulence |
| gene3848 | Chromosome | BMEI1329 | PHI:5423 | Brucella melitensis | reduced virulence |
| gene3852 | Chromosome | SSBX | PHI:2737 | Xanthomonas oryzae | reduced virulence |
| gene3859 | Chromosome | fruR | PHI:2672 | Salmonella enterica | reduced virulence |
| gene3860 | Chromosome | potA | PHI:6319 | Streptococcus pneumoniae | reduced virulence |
| gene3861 | Chromosome | VK055_1939 | PHI:7533 | Klebsiella pneumoniae | unaffected pathogenicity |
| gene3863 | Chromosome | orf245 | PHI:611 | Salmonella enterica | reduced virulence |
| gene3873 | Chromosome | SsFdh1 (SS1G_10135) | PHI:9519 | Sclerotinia sclerotiorum | reduced virulence |
| gene3874 | Chromosome | pspG | PHI:7661 | Salmonella enterica | unaffected pathogenicity |
| gene3875 | Chromosome | OxyR | PHI:3714 | Escherichia coli | reduced virulence |
| gene3879 | Chromosome | iroC | PHI:9461 | Salmonella enterica | reduced virulence |
| gene3883 | Chromosome | CSH1 | PHI:419 | Candida albicans | reduced virulence |
| gene3905 | Chromosome | RED3 | PHI:2836 | Bipolaris maydis | reduced virulence |
| gene3908 | Chromosome | iaaH-1 | PHI:4171 | Pseudomonas savastanoi | reduced virulence |
| gene3911 | Chromosome | pgi | PHI:9310 | Erwinia amylovora | reduced virulence |
| gene3913 | Chromosome | FVEG_12521 | PHI:3389 | Fusarium verticillioides | unaffected pathogenicity |
| gene3917 | Chromosome | potA | PHI:6319 | Streptococcus pneumoniae | reduced virulence |
| gene3922 | Chromosome | agaR2 | PHI:5300 | Streptococcus suis | reduced virulence |
| gene3924 | Chromosome | kdgR | PHI:4175 | Dickeya solani | unaffected pathogenicity |
| gene3925 | Chromosome | gntK | PHI:6701 | Vibrio cholerae | reduced virulence |
| gene3926 | Chromosome | edd | PHI:6698 | Vibrio cholerae | reduced virulence |
| gene3929 | Chromosome | ICL1 | PHI:8917 | Candida glabrata | unaffected pathogenicity |
| gene3930 | Chromosome | AoMls | PHI:4021 | Arthrobotrys oligospora | reduced virulence |
| gene3931 | Chromosome | metA | PHI:9313 | Erwinia amylovora | reduced virulence |
| gene3932 | Chromosome | OxyR | PHI:3714 | Escherichia coli | reduced virulence |
| gene3943 | Chromosome | XC_0509 | PHI:3942 | Xanthomonas campestris | reduced virulence |
| gene3945 | Chromosome | hupA | PHI:3074 | Edwardsiella tarda | reduced virulence |
| gene3949 | Chromosome | XC_0501 | PHI:3959 | Xanthomonas campestris | reduced virulence |
| gene3957 | Chromosome | ydcR | PHI:7225 | Salmonella enterica | reduced virulence |
| gene3959 | Chromosome | yedX | PHI:2651 | Salmonella enterica | unaffected pathogenicity |
| gene3961 | Chromosome | RpoB | PHI:7860 | Acinetobacter baumannii | reduced virulence |
| gene3985 | Chromosome | Pot13p | PHI:3689 | Candida albicans | unaffected pathogenicity |
| gene3990 | Chromosome | tatC | PHI:8759 | Salmonella enterica | reduced virulence |
| gene3991 | Chromosome | tatB | PHI:8758 | Salmonella enterica | reduced virulence |
| gene3992 | Chromosome | tatA | PHI:8757 | Salmonella enterica | reduced virulence |
| gene3999 | Chromosome | metE | PHI:9315 | Erwinia amylovora | reduced virulence |
| gene4000 | Chromosome | metR | PHI:9326 | Erwinia amylovora | unaffected pathogenicity |
| gene4013 | Chromosome | corA | PHI:2479 | Pectobacterium carotovorum | reduced virulence |
| gene4020 | Chromosome | cyaA | PHI:2950 | Pseudomonas syringae | reduced virulence |
| gene4034 | Chromosome | arnB | PHI:7949 | Klebsiella pneumoniae | unaffected pathogenicity |
| gene4036 | Chromosome | rmlA | PHI:7634 | Pseudomonas aeruginosa | reduced virulence |
| gene4038 | Chromosome | algD | PHI:7031 | Pseudomonas syringae | unaffected pathogenicity |
| gene4041 | Chromosome | wecA | PHI:4074 | Serratia marcescens | reduced virulence |
| gene4042 | Chromosome | Rho | PHI:8558 | Staphylococcus aureus | increased virulence (hypervirulence) |
| gene4043 | Chromosome | thioredoxin 1 | PHI:2644 | Salmonella enterica | reduced virulence |
| gene4044 | Chromosome | Lmo0866 | PHI:6510 | Listeria monocytogenes | unaffected pathogenicity |
| gene4047 | Chromosome | PrsA2 | PHI:6137 | Listeria monocytogenes | unaffected pathogenicity |
| gene4049 | Chromosome | SoxR | PHI:3092 | Pantoea stewartii | reduced virulence |
| gene4050 | Chromosome | ilvA | PHI:9318 | Erwinia amylovora | reduced virulence |
| gene4051 | Chromosome | Ilv3A | PHI:2638 | Aspergillus fumigatus | reduced virulence |
| gene4054 | Chromosome | MoIlv2 | PHI:3975 | Magnaporthe oryzae | loss of pathogenicity |
| gene4055 | Chromosome | yfiB | PHI:7572 | Klebsiella pneumoniae | unaffected pathogenicity |
| gene4057 | Chromosome | hdfR (Dda3937_04419) | PHI:8567 | Dickeya dadantii | reduced virulence |
| gene4066 | Chromosome | ireA | PHI:6654 | Escherichia coli | unaffected pathogenicity |
| gene4071 | Chromosome | SoxR | PHI:3092 | Pantoea stewartii | reduced virulence |
| gene4073 | Chromosome | argH | PHI:2632 | Staphylococcus aureus | reduced virulence |
| gene4074 | Chromosome | argG | PHI:9316 | Erwinia amylovora | reduced virulence |
| gene4076 | Chromosome | MoARG5,6 | PHI:5233 | Magnaporthe oryzae | loss of pathogenicity |
| gene4078 | Chromosome | ppc | PHI:9322 | Erwinia amylovora | unaffected pathogenicity |
| gene4079 | Chromosome | metF | PHI:9325 | Erwinia amylovora | unaffected pathogenicity |
| gene4081 | Chromosome | metB | PHI:9314 | Erwinia amylovora | reduced virulence |
| gene4082 | Chromosome | metJ | PHI:2695 | Pectobacterium atrosepticum | reduced virulence |
| gene4085 | Chromosome | fruR | PHI:2672 | Salmonella enterica | reduced virulence |
| gene4088 | Chromosome | ClpX | PHI:3004 | Staphylococcus aureus | reduced virulence |
| gene4094 | Chromosome | GPM2 | PHI:6486 | Mycobacterium tuberculosis | unaffected pathogenicity |
| gene4100 | Chromosome | Mqo | PHI:6190 | Staphylococcus aureus | reduced virulence |
| gene4104 | Chromosome | Smp | PHI:7699 | Pseudomonas syringae | unaffected pathogenicity |
| gene4105 | Chromosome | PhzF1 | PHI:7297 | Pseudomonas aeruginosa | reduced virulence |
| gene4106 | Chromosome | THR1 | PHI:7696 | Sclerotinia sclerotiorum | unaffected pathogenicity |
| gene4107 | Chromosome | XC_2801 | PHI:3260 | Xanthomonas campestris | reduced virulence |
| gene4111 | Chromosome | pfkA | PHI:9309 | Erwinia amylovora | reduced virulence |
| gene4135 | Chromosome | cpxP | PHI:8515 | Citrobacter rodentium | unaffected pathogenicity |
| gene4136 | Chromosome | cpxR | PHI:8512 | Citrobacter rodentium | unaffected pathogenicity |
| gene4137 | Chromosome | cpxA | PHI:8513 | Citrobacter rodentium | unaffected pathogenicity |
| gene4140 | Chromosome | pdh | PHI:9338 | Streptococcus suis | reduced virulence |
| gene4144 | Chromosome | envC | PHI:5573 | Salmonella enterica | reduced virulence |
| gene4149 | Chromosome | Als1 | PHI:595 | Parastagonospora nodorum | reduced virulence |
| gene4153 | Chromosome | cps2J | PHI:4130 | Streptococcus suis | reduced virulence |
| gene4154 | Chromosome | rfaL (ocw) | PHI:3106 | Escherichia coli | reduced virulence |
| gene4158 | Chromosome | pgaC | PHI:3448 | Klebsiella pneumoniae | reduced virulence |
| gene4161 | Chromosome | waaA | PHI:7635 | Pseudomonas aeruginosa | unaffected pathogenicity |
| gene4167 | Chromosome | radC | PHI:4701 | Streptococcus pneumoniae | reduced virulence |
| gene4171 | Chromosome | pyrE | PHI:7662 | Salmonella enterica | unaffected pathogenicity |
| gene4179 | Chromosome | adeN | PHI:7809 | Acinetobacter baumannii | increased virulence (hypervirulence) |
| gene4182 | Chromosome | SpoT | PHI:9162 | Salmonella enterica | reduced virulence |
| gene4184 | Chromosome | Mfd | PHI:6483 | Bacillus cereus | loss of pathogenicity |
| gene4187 | Chromosome | HilE (STM14_4514) | PHI:9177 | Salmonella enterica | reduced virulence |
| gene4194 | Chromosome | glnA | PHI:7742 | Salmonella enterica | reduced virulence |
| gene4195 | Chromosome | barA | PHI:562 | Salmonella enterica | unaffected pathogenicity |
| gene4196 | Chromosome | ntrC | PHI:7250 | Burkholderia cenocepacia | unaffected pathogenicity |
| gene4201 | Chromosome | dsbA (ROD_38991) | PHI:8691 | Citrobacter rodentium | loss of pathogenicity |
| gene4202 | Chromosome | rdoA (ROD_39001) | PHI:8692 | Citrobacter rodentium | unaffected pathogenicity |
| gene4210 | Chromosome | CcpA | PHI:4564 | Enterococcus faecium | reduced virulence |
| gene4211 | Chromosome | orf408 | PHI:612 | Salmonella enterica | reduced virulence |
| gene4213 | Chromosome | VK055_1939 | PHI:7533 | Klebsiella pneumoniae | unaffected pathogenicity |
| gene4214 | Chromosome | StpC | PHI:6324 | Staphylococcus aureus | unaffected pathogenicity |
| gene4216 | Chromosome | THR1 | PHI:7696 | Sclerotinia sclerotiorum | unaffected pathogenicity |
| gene4217 | Chromosome | AlsS | PHI:8543 | Enterococcus faecalis | reduced virulence |
| gene4219 | Chromosome | ovrB (Z0346) | PHI:9508 | Escherichia coli | reduced virulence |
| gene4221 | Chromosome | QseB | PHI:6882 | Salmonella enterica | increased virulence (hypervirulence) |
| gene4222 | Chromosome | qseC | PHI:3709 | Pectobacterium carotovorum | reduced virulence |
| gene4232 | Chromosome | atpB | PHI:9436 | Salmonella enterica | reduced virulence |
| gene4237 | Chromosome | atpG | PHI:8019 | Staphylococcus aureus | reduced virulence |
| gene4238 | Chromosome | yscN | PHI:7902 | Yersinia pseudotuberculosis | unaffected pathogenicity |
| gene4240 | Chromosome | lpxA | PHI:8042 | Acinetobacter baumannii | reduced virulence |
| gene4241 | Chromosome | glmS | PHI:9308 | Erwinia amylovora | reduced virulence |
| gene4244 | Chromosome | Rv0930 | PHI:3635 | Mycobacterium tuberculosis | unaffected pathogenicity |
| gene4245 | Chromosome | pstB | PHI:3412 | Xanthomonas citri | loss of pathogenicity |
| gene4251 | Chromosome | mnmE | PHI:9270 | Streptococcus suis | reduced virulence |
| pA_gene0003 | Plasmid 1 | DipA | PHI:3042 | Pseudomonas aeruginosa | reduced virulence |
| pA_gene0042 | Plasmid 1 | hopA1 | PHI:3165 | Pseudomonas cichorii | unaffected pathogenicity |
| pA_gene0052 | Plasmid 1 | hopA1 | PHI:3165 | Pseudomonas cichorii | unaffected pathogenicity |
| pA_gene0056 | Plasmid 1 | HMPREF0351_10118 (WxL locusC) | PHI:5205 | Enterococcus faecium | reduced virulence |
| pA_gene0060 | Plasmid 1 | PCC21 _023220 | PHI:3711 | Pectobacterium carotovorum | reduced virulence |
| pA_gene0064 | Plasmid 1 | aphB | PHI:5548 | Vibrio cholerae | reduced virulence |
| pA_gene0081 | Plasmid 1 | ECA2008 | PHI:7690 | Pectobacterium atrosepticum | increased virulence (hypervirulence) |
| pA_gene0082 | Plasmid 1 | ECA2008 | PHI:7690 | Pectobacterium atrosepticum | increased virulence (hypervirulence) |
| pA_gene0086 | Plasmid 1 | cheM | PHI:6983 | Salmonella enterica | reduced virulence |
| pA_gene0094 | Plasmid 1 | entA | PHI:4077 | Bacillus cereus | reduced virulence |
| pA_gene0097 | Plasmid 1 | vtlR | PHI:5012 | Brucella abortus | reduced virulence |
| pA_gene0098 | Plasmid 1 | TOXA | PHI:60 | Bipolaris zeicola | lethal |
| pA_gene0104 | Plasmid 1 | vtlR | PHI:5012 | Brucella abortus | reduced virulence |
| pA_gene0110 | Plasmid 1 | ovrB (Z0346) | PHI:9508 | Escherichia coli | reduced virulence |
| pA_gene0112 | Plasmid 1 | gnoA | PHI:3277 | Aspergillus fumigatus | unaffected pathogenicity |
| pA_gene0114 | Plasmid 1 | XC_2801 | PHI:3260 | Xanthomonas campestris | reduced virulence |
| pA_gene0126 | Plasmid 1 | NorA (AflE) | PHI:7768 | Aspergillus flavus | reduced virulence |
| pA_gene0140 | Plasmid 1 | fruR | PHI:6503 | Streptococcus pyogenes | unaffected pathogenicity |
| pA_gene0141 | Plasmid 1 | KdsD | PHI:7067 | Francisella tularensis | reduced virulence |
| pA_gene0145 | Plasmid 1 | gnoA | PHI:3277 | Aspergillus fumigatus | unaffected pathogenicity |
| pA_gene0148 | Plasmid 1 | BglA3 | PHI:6501 | Streptococcus pneumoniae | reduced virulence |
| pA_gene0195 | Plasmid 1 | CadA | PHI:7386 | Listeria monocytogenes | unaffected pathogenicity |
| pA_gene0198 | Plasmid 1 | hopA1 | PHI:3165 | Pseudomonas cichorii | unaffected pathogenicity |
| pB_gene0008 | Plasmid 2 | hns | PHI:6369 | Escherichia coli | increased virulence (hypervirulence) |
| pB_gene0009 | Plasmid 2 | ymoA | PHI:3819 | Yersinia pestis | increased virulence (hypervirulence) |
| pB_gene0019 | Plasmid 2 | cagY | PHI:8882 | Helicobacter pylori | reduced virulence |
| pB_gene0026 | Plasmid 2 | virB1 | PHI:7604 | Brucella abortus | reduced virulence |
