# Supplementary figures and images for: Discovery and characterization of a novel pathogen Erwinia pyri sp. nov. associated with pear dieback: taxonomic insights and genomic analysis
Source: Front Microbiol. 2024 May 9;15:1365685. doi: 10.3389/fmicb.2024.1365685 (PMC11111954; doi:10.3389/fmicb.2024.1365685)

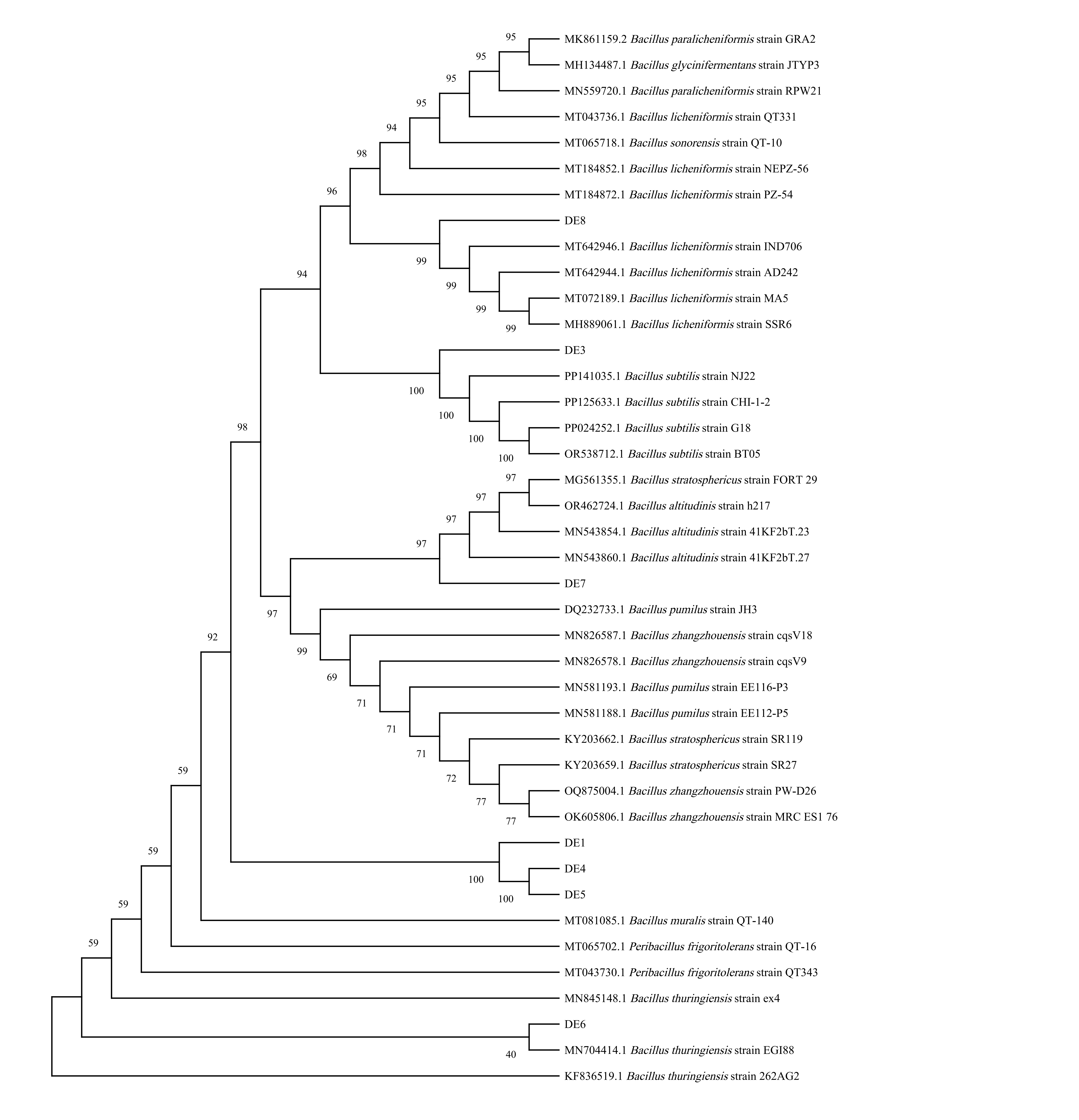

Supplement: Supplementary Figure S1 — Phylogenetic tree of isolate DE1, DE3-DE8 based on the 16S rDNA sequence. [file Image_1.PNG]

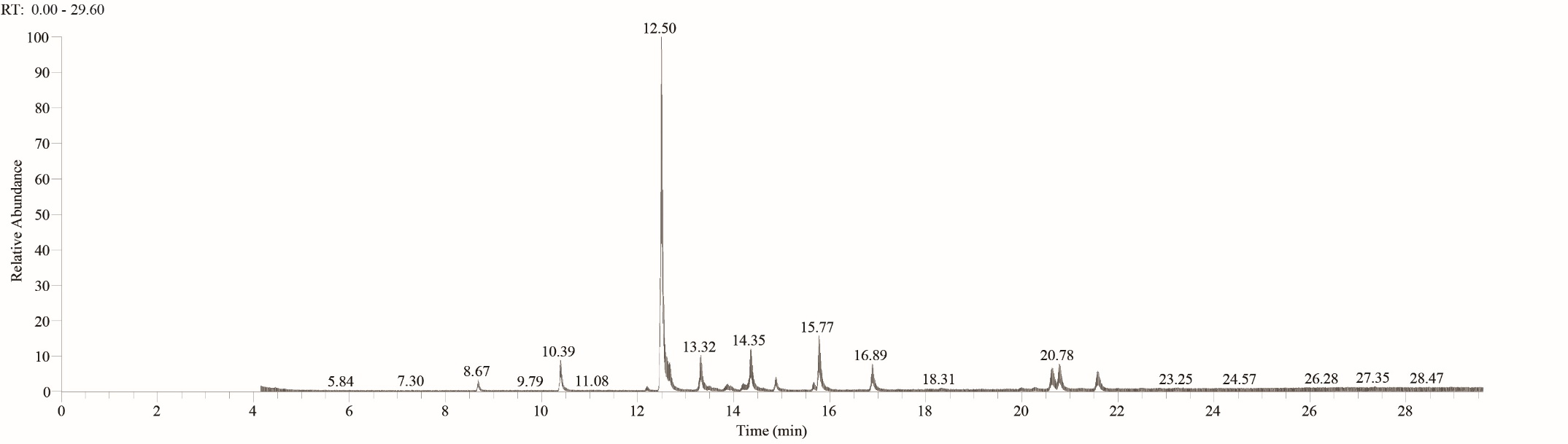

Supplement: Supplementary Figure S2 — Fatty acid profile of strain DE2. [file Image_2.PNG]

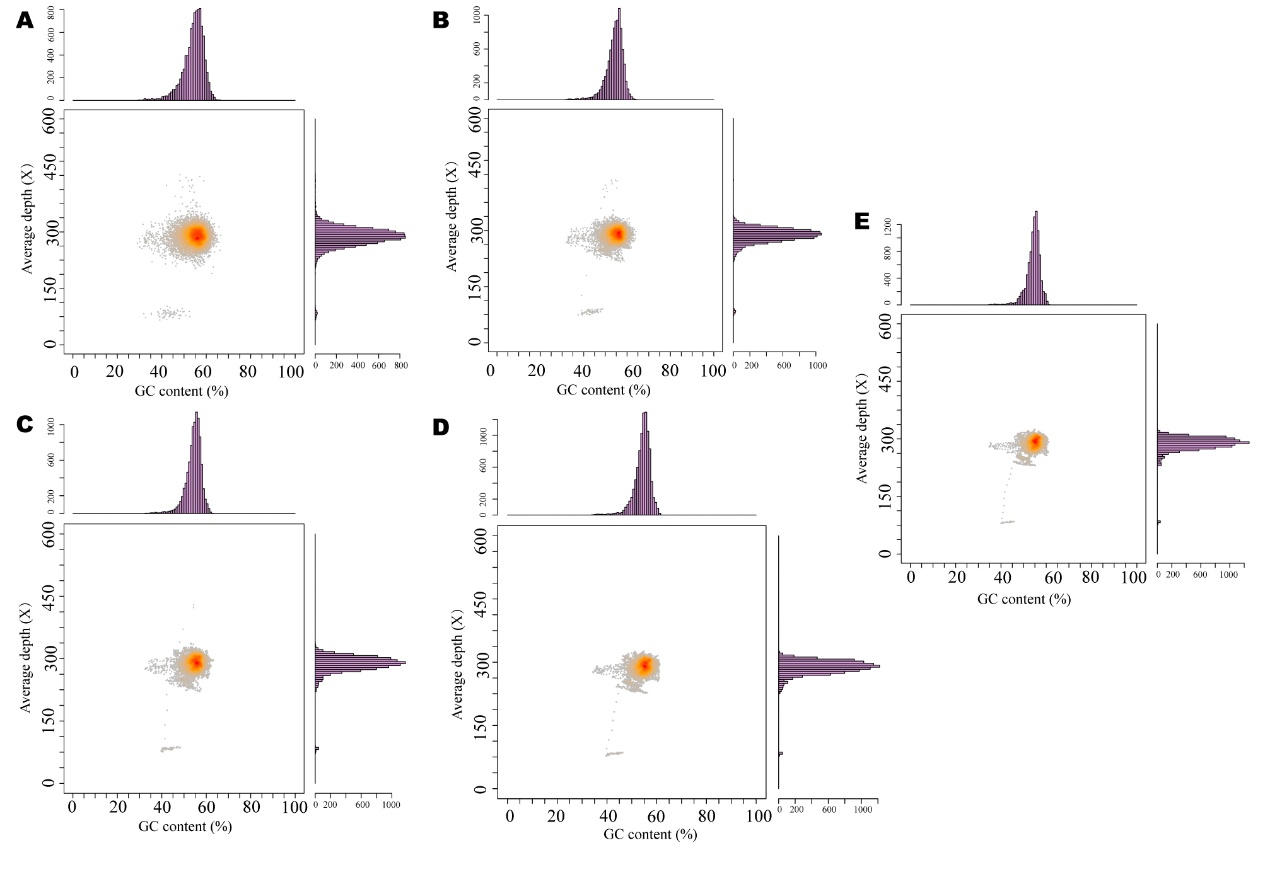

Supplement: Supplementary Figure S3 — GC-depth distribution of strain DE2. GC content and mean depth with a (A) 1-, (B) 3-, (C) 5-, (D) 8-, and (E) 10-kb windows. [file Image_3.PNG]
